# Localized Hydrogen Release Driven by Mg–Pd Microgalvanic Cells from 3D‐Printed Microneedles Reverses Redox Homeostasis for Psoriasis Treatment

**DOI:** 10.1002/advs.78709

**Published:** 2026-11-09

**Authors:** Xinyue Shao, Zongyan He, Ning Yang, Lixuan Yin, Xujie Sun, Jingtong Qi, Xiaolong Feng, Yihui Zhang, Yiran Liu, Zhengwei Huang, Tianqun Lang

**Affiliations:** ^1^ School of Life Science and Technology ShanghaiTech University Shanghai China; ^2^ Lingang Laboratory Shanghai China; ^3^ School of Pharmacy East China Normal University Shanghai China; ^4^ State Key Laboratory of Bioactive Molecules and Druggability Assessment Guangdong Basic Research Center of Excellence for Natural Bioactive Molecules and Discovery of Innovative Drugs College of Pharmacy Jinan University Guangzhou China

**Keywords:** 3D printing microneedle, Hydrogen therapy, Magnesium galvanic cells, Oxidative stress, Psoriasis

## Abstract

Psoriasis therapy faces multiple challenges, including refractory, recurrence, and various comorbidities, attributed to its complex pathogenesis driven by aberrant keratinocyte proliferation, oxidative stress imbalance, and inflammation. These three factors form a vicious cycle, and cycle disruption holds great therapeutic potential for psoriasis. However, current therapies merely focus on symptom relief or anti‐inflammatory effects alone with ignoring the essential cycle. To address the issues, a magnesium (Mg)‐palladium (Pd) micro‐galvanic cell (MP) with spontaneous electrochemical corrosion was constructed. MP released hydrogen (H_2_), inhibiting keratinocyte hyperproliferation, scavenging reactive oxygen species (ROS) and •OH, activating KEAP1‑NRF2 antioxidant signaling pathway, while generated Mg^2+^ exerted anti‑inflammatory effects. Furthermore, hollow microneedles loaded with MP (MP‐MN) were fabricated via 3D printing to penetrate the stratum corneum for deep H_2_ delivery. In the imiquimod‐induced psoriatic mouse model, MP‑MN topical treatment restored skin thickness, ameliorated oxidative stress, decreased splenic Th17/Treg ratio, and lowered both local and systemic pro‑inflammatory cytokine levels. Collectively, MP‑MN presented a comprehensive strategy for anti‐inflammatory, antioxidant, and immune‐regulatory effects in psoriasis, broadening the applications of H_2_ and micro‐galvanic cells in skin disorders.

## Graphical abstract

A 3D printing microneedle (MP‐MN), loading magnesium‐based micro‐galvanic cells (MP), is designed for improving psoriasis. H_2_ generated from MP‐MN scavenges ROS and •OH, actives NRF2‐KEAP1 pathway, and decreases inflammation, thereby effectively restoring the oxidative stress homeostasis of psoriatic skin and suppressing the excessive proliferation of keratinocytes in the psoriasis mouse model. MP‐MN system facilitated the transdermal delivery of H_2_, providing a basis for further investigation of hydrogen‐based therapy for psoriasis.

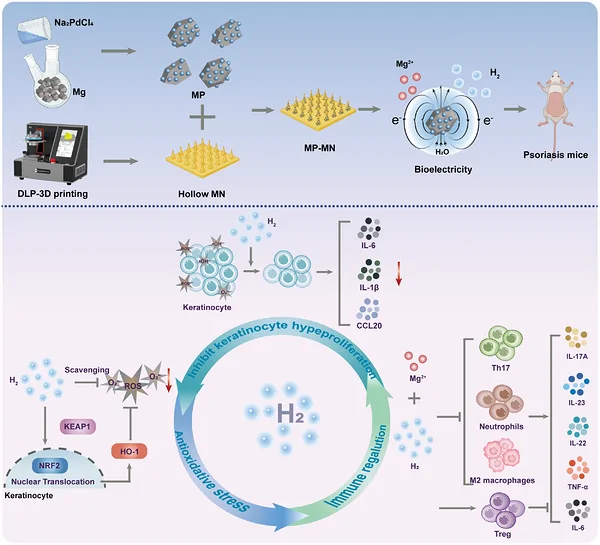

## 1 Introduction

Psoriasis is an immune‐mediated chronic skin disease characterized by erythema, pruritus, desquamation, and inflammatory plaques, affecting approximately 2%–3% of the global population [1, 2]. As a refractory disease, psoriasis results in multiple complications, including psoriatic arthritis [3], psoriatic inflammatory bowel disease [4], and depression [5]. Aberrant keratinocyte proliferation, oxidative stress imbalance, and immune dysregulation serve as key pathogenic drivers [6, 7]. Upregulated reactive oxygen species (ROS) trigger oxidative stress imbalance, which induces keratinocyte proliferation [7], disrupts intracellular antioxidant defense mechanisms, and activates multiple inflammatory signaling pathways such as the nuclear factor‐kappa B (NF‐κB) signaling pathway [8], thereby facilitating the release of chemokines and cytokines [9]. The mediators mentioned above further recruit macrophages, neutrophils, and T cells to lesional sites [9]. Subsequently, antigen‐presenting cells residing in psoriatic lesions and lymphoid organs produce proinflammatory cytokines, including interleukin (IL)‐23, IL‐1β, IL‐6, and tumor necrosis factor (TNF)‐α, which drive the differentiation of naïve CD4^+^ T cells into Th17 cells. Th17 cells subsequently secrete and release IL‐17 and IL‐22 [10, 11]. IL‐17, as a major driver of psoriasis, induces persistent inflammation and excessive keratinocyte hyperproliferation [6]. This process further amplifies ROS production, thereby exacerbating oxidative stress, ultimately forming a self‐perpetuating positive feedback vicious cycle. Therefore, breaking the positive‐feedback loop as mentioned above by alleviating local oxidative stress remains a rational and effective potential therapeutic strategy for psoriasis. However, conventional therapies like corticosteroids and emerging biologic agents primarily focus on suppressing inflammation without fundamentally addressing this pathogenic loop, and these treatments also carry drawbacks such as skin irritation and high cost [12, 13]. Previous antioxidant strategies, such as small‐molecule antioxidants, nanozymes, and natural compounds, have issues of non‐selective ROS scavenging, poor skin penetration, and insufficient local bioavailability.

Recent studies have demonstrated that hydrogen (H_2_) with reduced properties can disrupt the vicious cycle as noted above by preferentially scavenging hydroxyl radicals (•OH) and ROS [14, 15], subsequently contributing to activating nuclear factor erythroid 2‐related factor 2 (NRF2)‐Kelch‐like ECH‐associated protein 1 (KEAP1) anti‐oxidative stress pathway and downregulating levels of pro‐inflammatory cytokines [16]. H_2_ is cost‐effective without the immunosuppressive risk of biologic agents. Owing to antioxidant and anti‐inflammatory properties, molecule H_2_ therapy has attracted considerable attention in biomedicine, leading to extensive investigation into oxidative stress‐related conditions, including osteoarthritis [17], psoriasis [18], thrombosis [19], wound healing [20], and insulin resistance [21]. However, due to the low solubility in aqueous media and high diffusivity, H_2_ fails to reach effective therapeutic concentrations at the lesion site, thereby limiting its therapeutic efficacy. Hydrogen‐storing/generating materials with high encapsulation efficiency provide a promising strategy for achieving H_2_ delivery. Rational design of hydrogen‐generating materials can produce and release H_2_ at lesion sites efficiently. Nevertheless, the application of H_2_ therapy in psoriasis remains largely unexplored, and the hydrogen‐generating materials reported so far are limited to magnesium (Mg) microparticles and MgH_2_ [22, 23]. In addition, conventional Mg powders suffer from several drawbacks, such as surface passivation by a Mg(OH)_2_ layer and insufficient hydrogen generation kinetics. It has been reported that micro‐galvanic cells based on metals such as Mg, Mn, and Zn, fabricated via galvanic replacement reactions serve as a simple, low‐cost, stable, and effective approach for in situ H_2_ generation [21, 24, 25]. For instance, Mg‐Pt galvanic cells have been applied for in situ H_2_ production in the treatment of 4T1 and CT26 murine tumors [24]. An implantable Mn@Pt micro‐galvanic cell was utilized to achieve uric acid reduction, analgesia, and anti‐inflammation in gouty arthritis therapy [25]. Zn‐Fe micro‐nanoparticles released H_2_ in the stomach could remarkably improve insulin resistance [21]. Recent advances in electrotherapy have expanded from external electrical stimulation to engineered electroactive platforms. Engineered electrotherapy platforms integrating electroactive materials for precision cancer therapy [26] and emerging electrotherapeutic technologies as a biomedical engineering paradigm have been summarized [27, 28].

Local and in situ delivery of high‐concentration hydrogen is essential for maximizing its antioxidant efficacy. Microneedles (MNs) loaded with high‐efficiency hydrogen‐producing materials represent an optimal strategy for in situ transdermal delivery of hydrogen in psoriasis treatment [29]. As a minimally invasive, painless transdermal drug delivery system capable of loading drugs and nano formulations, MNs exhibit excellent patient compliance and operability. MNs precisely and reversibly penetrate the epidermal layer of lesional skin to release functional substances, which is highly suitable for localized administration of skin diseases [30]. Recently, digital light processing (DLP)‐based 3D printing technology has been applied to fabricate personalized MNs in the biomedical field due to its flexibility, simplicity, and reproducibility [31, 32]. In addition, it enables the rapid fabrication of complex MN arrays with diverse architectures, including hollow MNs, barbed MNs, and bullet‐shaped MNs [32]. Such a customizable structural design tailored to specific application requirements can maximally meet the demands of local precision therapy. Therefore, MNs fabricated by 3D printing are potential platforms for loading hydrogen‐generating materials for psoriasis therapy.

To address the limitations of H_2_ therapy in psoriasis, this study developed a 3D‐printed hollow microneedle system loaded with Mg–Pd micro‐galvanic cells (designated MP‐MN) for efficient in situ hydrogen generation at psoriatic lesions (Scheme 1). Pd^2+^ was uniformly reduced in situ to Pd microparticles on the Mg powder surface via galvanic replacement, forming Mg–Pd micro‐galvanic cells (MP). The hollow MN structure with pores enabled efficient loading, storage, and transdermal delivery of MP. After insertion into the skin, the interstitial fluid of the skin tissue acted as the electrolyte, triggering spontaneous redox reactions of MP to generate H_2_, Mg^2+^, and bioelectricity. H_2_ reduced intracellular ROS levels in keratinocytes, suppressing overoxidation, hyperproliferation, and pro‐inflammatory cytokine secretion. Moreover, H_2_ activated the KEAP1‐NRF2 pathway, triggering NRF2 nuclear translocation and upregulating the downstream antioxidant enzyme heme oxygenase‐1 (HO‐1). The scavenging of ROS and restoration of oxidative stress homeostasis inhibited the pro‐inflammatory NF‐κB signaling pathway. The reaction product Mg^2+^ further amplified anti‐inflammatory effects. Psoriasis‐like skin repair subsequently alleviated systemic inflammation, as reflected by reduced Th17 cells, neutrophil infiltration, and upregulation of Tregs and M2 macrophages in the spleen. In summary, MP‐MN disrupted the vicious positive‑feedback loop among keratinocyte hyperproliferation, oxidative stress, and inflammation driven by immune dysregulation, ultimately attenuating psoriasis progression. MP‐MN provides preliminary preclinical evidence supporting hydrogen‐based therapy for psoriasis and the transdermal delivery of H_2_.

**SCHEME 1:**
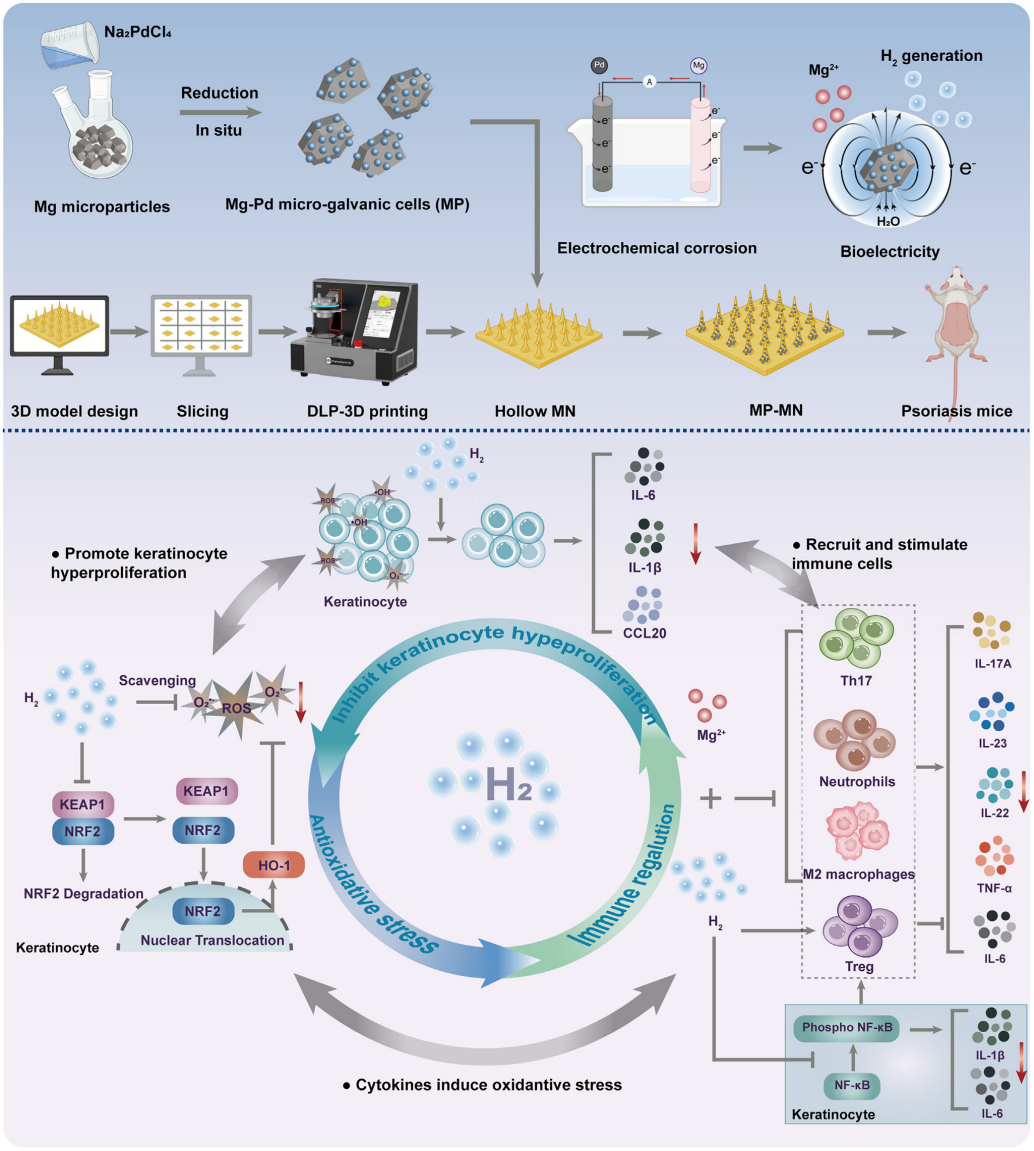
Schematic illustration of the preparation of MP‐MN and its antioxidant stress and anti‐inflammatory mechanisms for psoriasis therapy.

## 2 Results and Discussion

### 2.1 Characterization of the Mg‐Pd Microgalvanic Cell

MP was fabricated via in situ galvanic replacement technology. The standard electrode potential difference between Mg (Mg^2+^/Mg: −2.372 V) and Pd (PdCl_4_^2−^/Pd: 0.951 V) was 3.323 V, providing a sufficient driving force for the replacement reaction. Pd^2+^ from Na_2_PdCl_4_ solution was uniformly reduced in situ on the Mg powder surface, depositing metallic Pd microparticles that attached to the Mg surface and formed micro‐galvanic cells. After insertion into skin tissue, the interstitial fluid acted as an electrolyte, enabling spontaneous redox reactions. Specifically, Mg served as the negative electrode, losing electrons (e^−^) to generate Mg^2+^ (Mg − 2e^−^ = Mg^2+^), while Pd served as the positive electrode, accepting electrons to catalyze H_2_ evolution from H_2_O (2H_2_O + 2e^−^ = 2OH^−^ + H_2_).

The Pd^2+^ concentration and the reaction time directly had an impact on the loading capacity and uniformity of the Pd microparticles on the surface of Mg powder, which affected the efficiency of H_2_ generation. To screen and determine the optimal synthesis conditions, the morphology of MP prepared at various Pd^2+^ concentrations and reaction times was examined by scanning electron microscopy (SEM). Compared with the smooth surface of pristine Mg powder (Figure S1), the surface roughness of the Mg particles increased progressively as the Mg: Pd mass ratio (w/w) increased from 10: 1 to 1: 2 and the reaction time was extended from 1 h to 8 h, indicating continuous Pd reduction and deposition (Figure S2). However, once Mg: Pd ratios ≤ 1: 1 and reaction times exceeded 6 h, excessively rapid reduction resulted in phase non‐uniformity and severe Pd agglomeration, which was confirmed by energy dispersive spectrometry (EDS) mapping (Figures S2 and S3). Based on the above morphological observations, the H_2_ generation performance of MP was further evaluated. At a fixed reaction time of 4 h, Mg: Pd mass ratios of 1: 2, 1: 1, 2: 1, and 4: 1 were screened, and the 2: 1 ratio yielded the most uniform Pd distribution with sustained and stable H_2_ production (Figure S4). At Mg: Pd = 2: 1, varying reaction times (1, 2, and 4 h) were compared, further confirming that 4 h was the optimal reaction duration (Figure S5). Therefore, all subsequent MP syntheses were carried out at a Mg: Pd ratio of 2: 1 for 4 h.

The synthesis illustration of MP was shown in Figure 1A. The SEM images and EDS elemental mapping demonstrated a homogeneous distribution of Pd microparticles across the Mg surface (Figure 1B,C). X‐ray diffraction (XRD) patterns of MP revealed the emergence of Pd peaks, indicating that the Pd was successfully in situ replaced on the surface of Mg (Figure 1D). The principle of spontaneous electrochemical corrosion of MP in water, generating H_2_, Mg^2+^, and bioelectricity, was illustrated in Figure 1E. After exposure to PBS solution, MP rapidly reacted with H_2_O and produced abundant H_2_ bubbles, whereas slight H_2_ bubble formation was observed for Mg powders (Figure 1F). Given that methylene blue (MB) could be rapidly reduced by H_2_ to colorless MBH_2_, MB was employed as a redox probe to further detect H_2_ generation from MP (Figure S6A). Compared with the MB and Mg groups, the MP group exhibited marked fading of the blue color, accompanied by a pronounced decrease in the characteristic absorbance peak of MB (Figure 1G and Figure S6B,C). In contrast, the Mg group showed only a slight decline in the MB peak, indicating that MP possessed a substantially higher capacity to generate H_2_ (Figure 1G and Figure S6B). Furthermore, the quantitative analysis of H_2_ generation was performed by gas chromatography (GC) (Figure 1H). MP could continuously generate H_2_ over a 24 h period, whereas the H_2_ yield from Mg powders was relatively lower than that from MP, highlighting the superior H_2_ production capability of MP. In addition, the X‐ray photoelectron spectroscopy (XPS) patterns of MP suggested that the signal of Mg (II) significantly increased after reaction with PBS, indicating electron loss and oxidation of MP to Mg (II) at the negative electrode (Figure 1I,J). XPS analysis of Mg powders revealed the presence of Mg (0) despite the generation of abundant Mg^2+^ after reaction with PBS (Figure S7). It was speculated that the Mg powder surface became passivated by a Mg(OH)_2_ layer formed during the reaction, which inhibited further corrosion of the underlying Mg. Besides, the electric current of Mg and MP was continuously monitored for 3500 s through an electrochemical workstation. The electric current generated by MP reached approximately 200 nA, whereas Mg produced a current of only about 1.6 nA in the aqueous phase, convincingly demonstrating the bioelectricity‐generating capability of MP (Figure 1K,L). The spontaneous reaction of a metallic micro‐galvanic cell represented a typical electrochemical corrosion process, in which a higher corrosion potential hindered the anodic oxidation reaction and consequently diminished the H_2_ generation capacity [33]. Compared to Mg powders, the Tafel curve revealed the decrease in corrosion potential of the MP due to the existence of the micro‐galvanic cell structure (Figure 1M,N), which elucidated the good H_2_ generation capacity and the enhanced reactivity of MP in the aqueous phase. The Nyquist plots showed a markedly smaller semicircle radius for MP than for Mg through electrochemical impedance spectroscopy (EIS), indicating that the Mg–Pd micro‐galvanic cell structure effectively reduced the impedance of Mg (Figure S8). It also proved that Pd deposition facilitated electron transfer. The reduced impedance and more negative corrosion potential of MP together supported its enhanced H_2_ generation efficiency.

**FIGURE 1:**
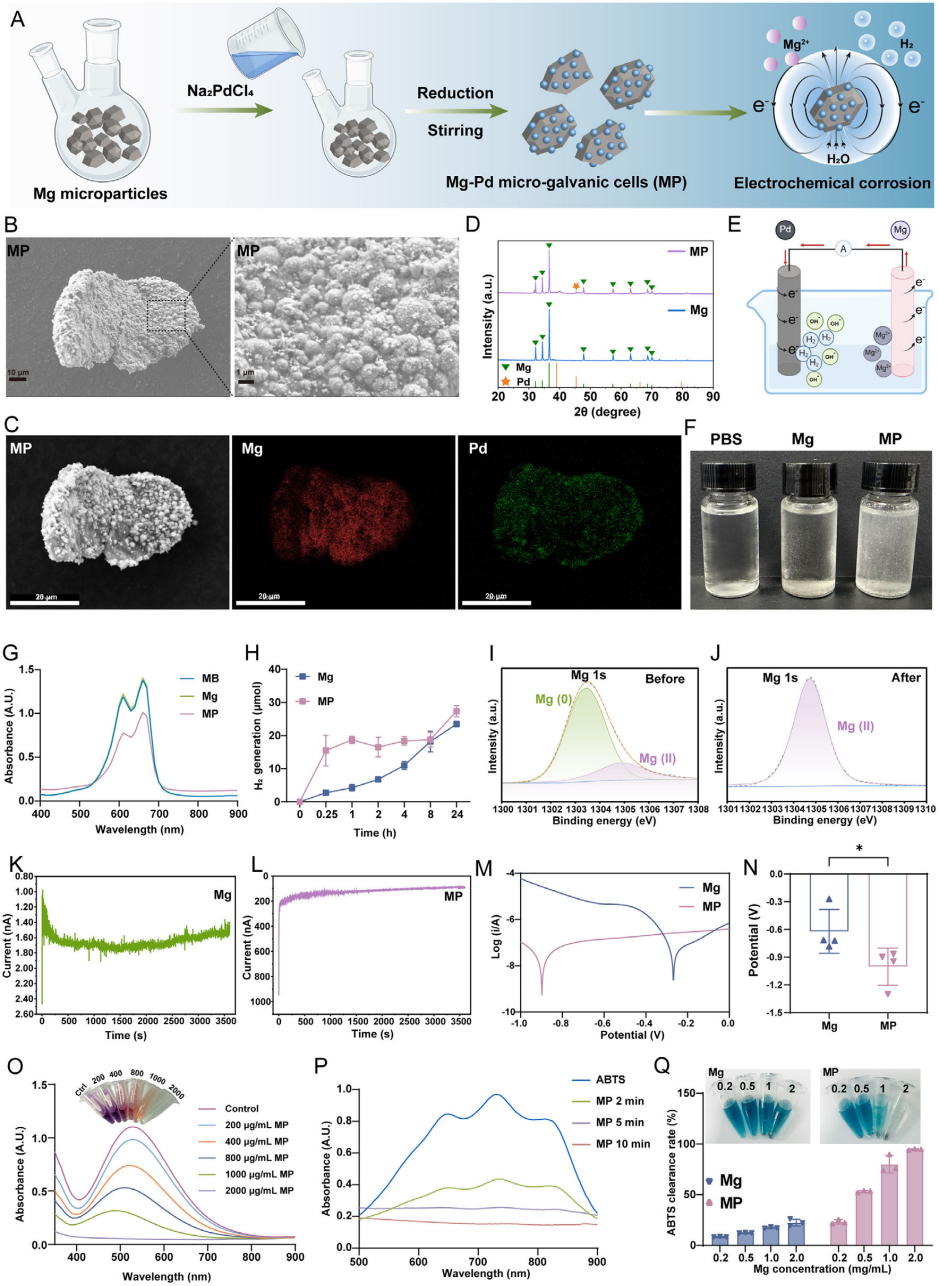
Preparation and characterization of MP. (A) Schematic diagram of the synthesis process and electrochemical corrosion of MP. (B) The representative SEM images of MP (Scale bar: 10 µm; Scale bar: 1 µm). (C) The representative EDS mapping of MP (Scale bar: 20 µm). (D) The XRD patterns of Mg and MP. (E) Schematic illustration of H_2_ production mechanism. (F) Photograph displaying H_2_ generation in PBS from Mg and MP. (G) Absorption spectra of the MB and MB solution with Mg or MP added after 20 min. (H) H_2_ release from Mg and MP in PBS. (I‐J) XPS spectra of MP before (I) and after (J) reaction with PBS. (K,L) Electric current variation of Mg (K) and MP (L) in 3500 s. (M) The Tafel curve of Mg and MP. (N) The corrosion potentials of Mg and MP. (O) Absorption spectra and color changes of •OH in assay solution incubated with different concentrations of MP. (P) Time‐dependent absorption spectra of ABTS^•+^ after incubation with MP. (Q) Free radical scavenging ratio and color changes of Mg and MP with different concentrations against ABTS^•+^. Data were shown as the mean ± SD. For each group, *n* = 3 independent samples in (H, Q); *n* = 4 independent samples in (N). ^*^ *p* < 0.05.

Subsequently, the antioxidant properties of the H_2_‐producing MP were evaluated. MP concentration‑dependently reduced the 412 nm absorbance, demonstrating efficient scavenging of the oxidative metabolite H_2_O_2_ and potent antioxidant action of H_2_ (Figure S9). The scavenging of •OH by H_2_ released from MP increased progressively with MP concentration, with gradual fading of the detection solution from purple to lighter yellow and a concentration‐dependent decline in the characteristic •OH peak intensity at 510 nm (Figure 1O). Mg powders showed weak •OH scavenging, with limited decline in the 510 nm peak versus MP at equal concentration (Figure S10). Similarly, as MP concentration increased and the reaction time was extended, H_2_ released from MP reduced the ABTS^•^^+^ detection solution from blue to colorless, while the ABTS^•^^+^ scavenging ability of Mg was weak (Figure 1P,Q and Figure S11A). UV–vis spectra showed a corresponding decrease in the characteristic ABTS^•^^+^ peak at 734 nm, with the scavenging efficiency approaching 95% at an MP concentration of 2 mg/mL (Figure 1P,Q and Figure S11B), a conclusion further supported by electron spin resonance (ESR). As shown in Figure S12, the Control group distinctly detected the characteristic signals of TEMP–^1^O_2_ (singlet oxygen), DMPO–•OH, and DMPO–O_2_^−^ (superoxide anion). Mg powders generated a small amount of H_2_, which scavenged ROS (^1^O_2_, •OH, and O_2_^−^) and reduced all three signals. MP further decreased the ROS‐associated signal intensities, consistent with more effective ROS scavenging by the larger amount of H_2_ it generated. In summary, MP was successfully constructed via an in situ galvanic replacement strategy and exhibited excellent hydrogen‐generating and antioxidant activities, laying a solid foundation for subsequent in vitro and in vivo psoriasis therapy studies.

### 2.2 The In Vitro Antioxidant and Anti‐Inflammatory Capabilities of MP

Considering that aberrant activation and excessive proliferation of keratinocytes were hallmark features of psoriatic skin, the cell viability of Mg powders and MP at different concentrations toward human immortalized keratinocytes (HaCaT) was assessed with the CCK‐8 assay. The cell viability of HaCaT cells gradually diminished, accompanied by increases in MP concentrations, whereas the Mg group maintained favorable cell viability (Figure 2A). To mimic the oxidative and inflammatory state of keratinocytes in psoriatic skin, HaCaT cells were stimulated with TNF‐α. Cell proliferation of HaCaT cells was evaluated under various TNF‐α concentrations and incubation times. CCK‐8 assay results indicated that at all tested concentrations, TNF‐α stimulation for 24 h led to greater cell proliferation than stimulation for 12 h (Figure S13). Moreover, flow cytometry analysis showed that the TNF‐α group with 40 ng/mL exhibited the highest intracellular ROS levels (Figure S14). Accordingly, the TNF‐α concentration of 40 ng/mL was selected for subsequent in vitro experiments to induce cell proliferation.

**FIGURE 2:**
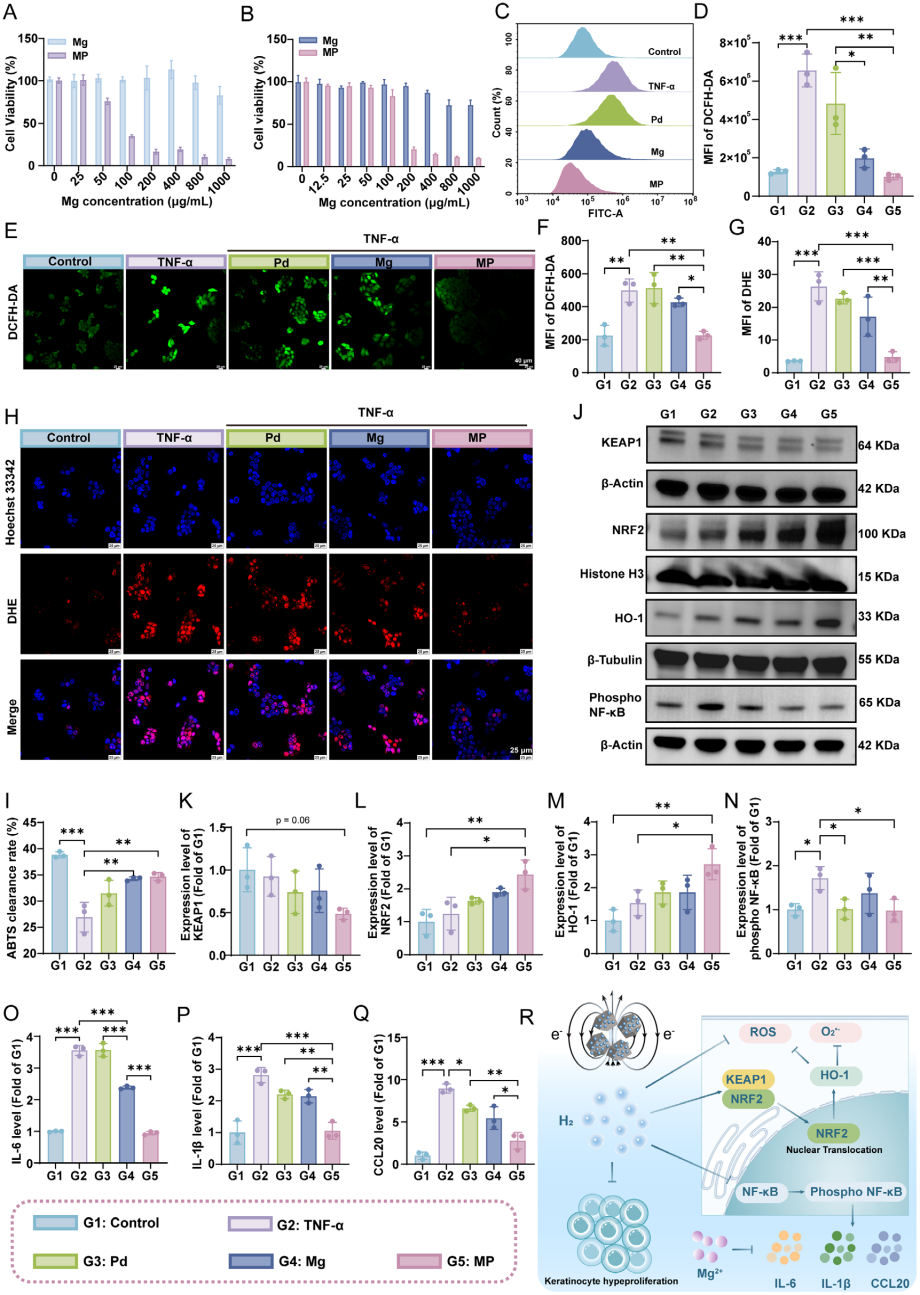
In vitro hydrogen therapy with MP. (A) Cell viabilities of HaCaT cells after Mg and MP treatments. (B) Cell viability of TNF‐α‐induced HaCaT cells after Mg and MP treatments. (C‐D) Flow cytometric examination (C) and quantitative analysis (D) of ROS in TNF‐α‐induced HaCaT cells after different treatments. (E,F) Confocal images (E) and statistical analysis (F) of ROS in TNF‐α‐induced HaCaT cells after various treatments. (G,H) Confocal images (G) and statistical analysis (H) of O_2_^−^ levels in TNF‐α‐induced HaCaT cells after various treatments. (I) ABTS clearance rate in TNF‐α‐induced HaCaT cells after different treatments. (J) Western blotting analysis of NRF2, KEAP1, HO‐1, and phospho‐NF‐κB. (K–N) Quantitative analysis of expression levels of KEAP1 (K), NRF2 (L), HO‐1 (M), and phosphor‐NF‐κB (N). (O‐Q) Relative expression of IL‐6 (O), IL‐1β (P), and CCL20 (Q). (R) Schematic illustration showing the mechanism of H_2_ therapy with MP for keratinocyte proliferation, antioxidation, and anti‐inflammation. Data were shown as the mean ± SD. For each group, *n* = 4 independent samples in (A,B); *n* = 3 independent samples in (D, F‐G, I, K‐Q). ^*^ *p* < 0.05, ^**^ *p* < 0.01, ^***^ *p* < 0.001.

As shown in Figure 2B, the cell viability of TNF‐α‐induced HaCaT cells was inhibited by approximately 79.4% under 200 µg/mL MP treatment, while there was no obvious inhibition in the Mg group. MP effectively suppressed keratinocyte hyperproliferation, attributed to the abundant H_2_ generated. Moreover, the Pd loaded on MP did not compromise the viability of TNF‐α‐induced HaCaT cells (Figure S15). Notably, high concentrations of MP interfered with the CCK‐8 assay, whereas Mg and Pd alone exhibited negligible interference (Figure S16). This interference was markedly reduced by PBS washing before CCK‐8 addition. Annexin V/PI staining showed that MP at 50–800 µg/mL did not enhance apoptosis compared with untreated controls (Figure S17), suggesting that the reduced CCK‐8 signal might primarily reflect inhibition of cell proliferation rather than induction of cell death. It was found that elevated oxidative stress was closely associated with keratinocyte hyperproliferation and the progression of inflammation [7]. Since the ability of MP to generate a substantial amount of H_2_ was confirmed, the in vitro scavenging capacity of MP toward ROS was further investigated. The DCFH‐DA probe was employed to detect intracellular ROS levels in TNF‐α‐induced HaCaT cells. Quantitative flow cytometry analysis revealed that MP treatment reduced ROS generation by approximately 84.7% compared with the TNF‐α group (Figure 2C,D), indicating a pronounced advantage of H_2_ produced from MP in scavenging ROS levels. The weak green signal in the confocal images of the MP group also confirmed this result (Figure 2E,F). Meanwhile, a slight decrease in ROS content was also observed in the Mg group compared with the TNF‐α group (Figure 2E), which might be partly attributed to a small amount of H_2_ generated by Mg. Besides, the confocal images indicated that, compared to the TNF‐α group and Pd group, the red signal intensity representing intracellular O_2_^−^ content in TNF‐α‐induced HaCaT cells was decreased between Mg group and MP group (Figure 2G,H). The signal intensity of the MP group was reduced by a factor of 3.57 compared with the Mg group (Figure 2G). The levels of O_2_^−^ in the mitochondria after treatment with the MP group were also obviously reduced (Figure S18). The obvious upregulation of ABTS clearance rate in the MP group also provided evidence for the antioxidant capacity of H_2_ therapy (Figure 2I). MP treatment reduced the malondialdehyde (MDA) level in HaCaT cells and simultaneously elevated catalase (CAT) activity, preventing H_2_O_2_ accumulation and thus protecting cells from oxidative stress (Figure S19). These results collectively underscored that MP could effectively inhibit hyperproliferation of keratinocytes and restore oxidative stress balance by releasing H_2_.

The underlying mechanisms of H_2_ therapy in antioxidant regulation were further assessed. The KEAP1–NRF2 pathway is a pivotal regulatory mechanism governing cellular responses to oxidative stress [34]. Under normal conditions, NRF2 is sequestered in the cytoplasm by its inhibitor KEAP1, which targets NRF2 for ubiquitination and proteasomal degradation [35]. In contrast, under oxidative stress, KEAP1‐mediated inhibition is relieved, allowing NRF2 to translocate into the nucleus, where it activates the transcription of downstream antioxidant enzymes such as HO‐1 for counteracting ROS [36, 37]. Western blot assay results displayed that the expression level of KEAP1 in the Mg and MP groups was obviously downregulated (Figure 2J,K and Figure S20). Compared to the TNF‐α group, the nuclear localization of NRF2 showed a 1.96‐fold enhancement in MP‐treated TNF‐α‐induced HaCaT cells (Figure 2J,L and Figure S21). Meanwhile, the immunofluorescence staining of HaCaT cells showed that MP promoted nuclear translocation of NRF2 (Figure S22). Consistent with the results mentioned above, a marked increase in HO‐1 expression under MP treatment was also observed, approximately 1.77‐fold higher than that in the TNF‐α group, whereas Pd and Mg treatment failed to activate the pathway (Figure 2J,M and Figure S23).

Additionally, compared with the TNF‐α group, the levels of phosphorylated NF‐κB in the Mg and MP groups were also sharply reduced by 19.9% and 43.2%, respectively (Figure 2J,N and Figure S24). The suppression of phosphorylated NF‐κB signaling pathway would inhibit the secretion of various pro‐inflammatory cytokines [38]. To verify the hypothesis, the ELISA of key pro‐inflammatory cytokines, including IL‐6, IL‐1β, and CCL20, was conducted. The results suggested that MP treatment restrained the secretion of pro‐inflammatory cytokines in HaCaT cells under TNF‐α stimulation (Figure 2O–Q), elucidating the potent anti‐inflammatory capacity of MP. The Mg group exhibited lower expression of the pro‐inflammatory mediators IL‐6, IL‐1β, and CCL20 than the TNF‐α group, suggesting that the Mg component contributed to the anti‐inflammatory effect of MP. H_2_ treatment alone also effectively reduced intracellular ROS levels, indicating that H_2_ therapy contributes independently to the observed antioxidant effect (Figure S25). MP‐After, the products remaining after MP depletion, showed negligible intracellular ROS‐scavenging activity compared with MP (Figure S25). Together, these findings indicated that MP‐generated H_2_ contributed to the reduction of oxidative stress, whereas the Mg^2+^ contributed to the suppression of inflammation (Figure 2R), although potential contributions from other reaction‐associated processes could not be fully excluded.

In TNF‐α‐stimulated HaCaT cells, the NRF2 inhibitor ML385 partially reversed the MP‐mediated reduction in intracellular ROS levels (Figure S26). Western blot analysis revealed that ML385 attenuated the MP‐induced increase in HO‐1 expression and partially restored NF‐κB nuclear translocation, while reducing the preservation of IκBα by MP (Figure S27). The partial reversal of MP's antioxidant effect by ML385, together with the reduction in ROS observed with H_2_ alone, indicated that MP‐generated H_2_ might integrate the individual benefits of direct free‐radical scavenging and indirect activation of the NRF2 pathway, thereby exerting an enhanced antioxidant effect.

### 2.3 Characterization of 3D‐Printed Hollow Microneedle

To achieve effective MP delivery into the deeper layers of the stratum corneum, a DLP‐based 3D printing technique was employed to fabricate MP‐loaded hollow microneedles (MP‐MN) for transdermal delivery. Gelatin methacryloyl (GelMA) 90 was selected as the printing matrix to enhance the stiffness and hardness of the microneedle tips, while poly (ethylene glycol) diacrylate (PEGDA) 600 acted as a “shaping agent” to improve printing resolution. The fabrication process comprised six steps: model design, slicing, photopolymerization‐based printing, washing, drying, and material backfilling (Figure 3A). The microneedle patch featured a square substrate with 23 tips and two square openings on the base for MP loading (Figure 3B). The overview image of MP‐MN indicates that the microneedle sealing is relatively intact (Figure S28A), with a microneedle substrate width of 7.383 ± 0.061 mm. The distribution of microneedle elements was relatively uniform (Figure S28B). SEM imaging revealed that the MP‐MN tips exhibited a typical quadrangular pyramid morphology, with a base length of 0.924 ± 0.010 mm, a tip height of 1.111 ± 0.015 mm, and an inter‐tip spacing of 0.828 ± 0.017 mm (Figure 3C and Table S1). The coefficients of variation (CV) for all parameters were below 3%, demonstrating excellent batch‐to‐batch consistency (Table S1). Pores were designed on the pyramid surface to allow interstitial fluid to enter the cavity and react with MP (Figure 3C). The morphology of the blank MN was similar to that of MP‐MN (Figure S29). The microscope images visually showed that MP‐MN generated relatively uniform H_2_ bubbles when it was immersed in an aqueous solution and underwent spontaneous electrochemical corrosion (Figure 3D). Thus, the release of H_2_ from MP‐MN was evaluated. The MP loading amount was 2.88 ± 0.27 mg in all tips and 11.92 ± 0.80 mg per whole microneedle patch. GC quantification further demonstrated that MP‐MN produced approximately 114.57 µmol of H_2_ within 1 h (Figure 3E). The sum spectrum showed characteristic Mg and Pd peaks, and Mg content was 89.1 wt% (Figure S30). This corresponds to a theoretical maximum H_2_ yield of approximately 437 µmol from 11.92 mg MP, confirming that the measured H_2_ generated from MP‐MN in 1 h is within the stoichiometric limit. Although the local H_2_ concentration in the skin was limited by the finite volume of interstitial fluid and the slow diffusion of H_2_ through tissue, the H_2_ content in mouse skin after MP‐MN application reached 45.63 nmol/g at 48 h, indicating that MP‐MN still generated detectable H_2_ in skin tissue (Figure S31). To assess whether MP‐MN generated local potential changes in skin, skin potential was monitored for 25 min using an electrochemical workstation, both with and without MP‐MN application. The potential–time profiles showed a higher local potential in MP‐MN‐treated skin than in untreated skin (Figure S32), suggesting that MP‐MN might provide sustained local electrical stimulation over the measurement period. Meanwhile, inductively coupled plasma (ICP) analysis confirmed the continuous release of Mg^2+^, a byproduct of the galvanic reaction, providing a basis for Mg^2+^ involvement in anti‐inflammatory effects (Figure 3F).

**FIGURE 3:**
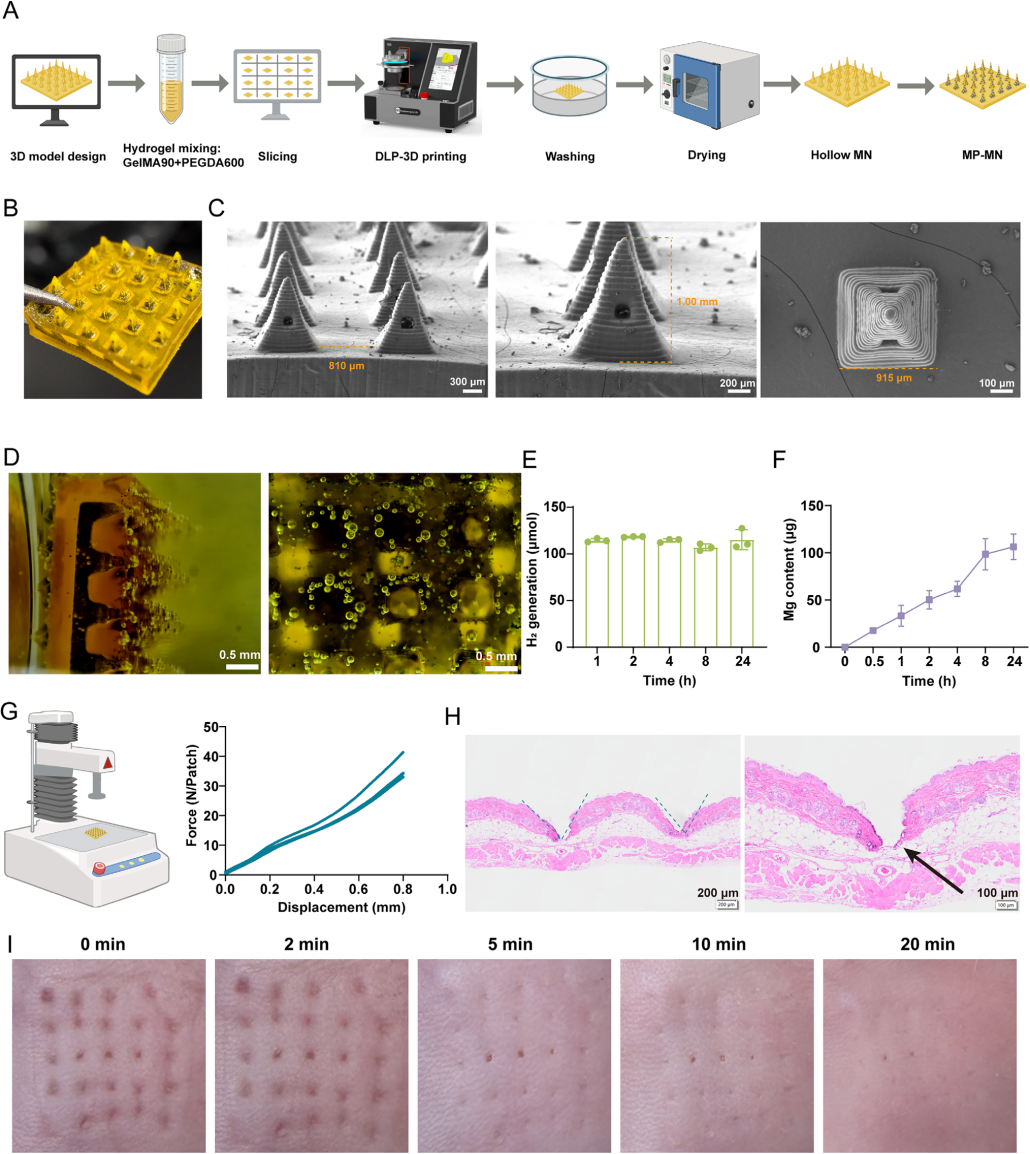
Fabrication process and characterization of MP‐MN. (A) Design and fabrication of MP‐MN via DLP‐based 3D printing technology. (B) Optical images of MP‐MN. (C) SEM images of MP‐MN from different angles (Scale bar: 300 µm; Scale bar: 200 µm; Scale bar: 100 µm). (D) Optical images of H_2_ bubble generation from MP‐MN in PBS (Scale bar: 0.5 mm). (E) H_2_ generation from MP‐MN in PBS. (F) Mg^2+^ content from MP‐MN in PBS. (G) Stress–displacement curve for MP‐MN. (H) Histological images of the skin after the application of MP‐MN (Scale bar: 200 µm; Scale bar: 100 µm). (I) Images showing the recovery of skin pores at various time points after MP‐MN treatment. Data were shown as the mean ± SD. For each group, *n* = 3 independent samples in (E‐G).

The mechanical properties of MP‐MN were evaluated by a texture analyzer. At a displacement of 800 µm, the force curve showed that the fracture force exceeded 36.31 ± 4.54 N per patch (Figure 3G) and the load on a single tip of MP‐MN was 13.81 ± 0.27 N (Figure S33), signifying sufficient mechanical characteristics to penetrate the skin barrier without breakage. The skin tissue following insertion of the MP‐MN patch was immediately fixed for histological examination. Tissue sections confirmed that the microneedles possessed a sharp conical structure with an appropriate height for penetrating the stratum corneum (Figure 3H). The recovery of skin micropores after insertion was subsequently monitored. An array of micropores was clearly visible on the murine dorsal skin following removal of the MP‐MN patch. In addition, the majority of these micropores disappeared within 20 min, with no erythema, bleeding, or other signs of skin irritation observed in the surrounding area (Figure 3I). Following three consecutive applications of MP‐MN, the MP‐MN‐induced skin punctures were no longer visible within 20 min (Figure S34). No visible erythema, swelling, bleeding, or skin erosion was observed (Table S2), supporting the favorable skin tolerability of MP‐MN under the tested conditions. As a result, the MP‐MN system with favorable H_2_‐generating performance and skin penetration capability was successfully constructed, and its dermal safety was preliminarily confirmed.

### 2.4 MP Therapy Alleviated Psoriasis Progression and Recurrence in Psoriatic Mouse Model

To explore the therapeutic effects of H_2_ from MP on psoriasis, MP‐MN was topically applied to the imiquimod (IMQ)‐induced psoriatic mouse model. The complete animal experiment process was shown in Figure 4A. The BALB/c mice were randomized into eight groups (*n* = 6), including Control, Model, Tapinarof (TAP), MP‐Cream, MN (Blank‐MN), Pd‐MN, Mg‐MN, and MP‐MN. The Control group received Vaseline as a blank control, while the remaining seven groups underwent psoriasis‐like skin induction via daily application of 5% IMQ cream. The Model group receiving no treatment and TAP group under Tapinarof administration served as negative and positive controls, respectively.

**FIGURE 4:**
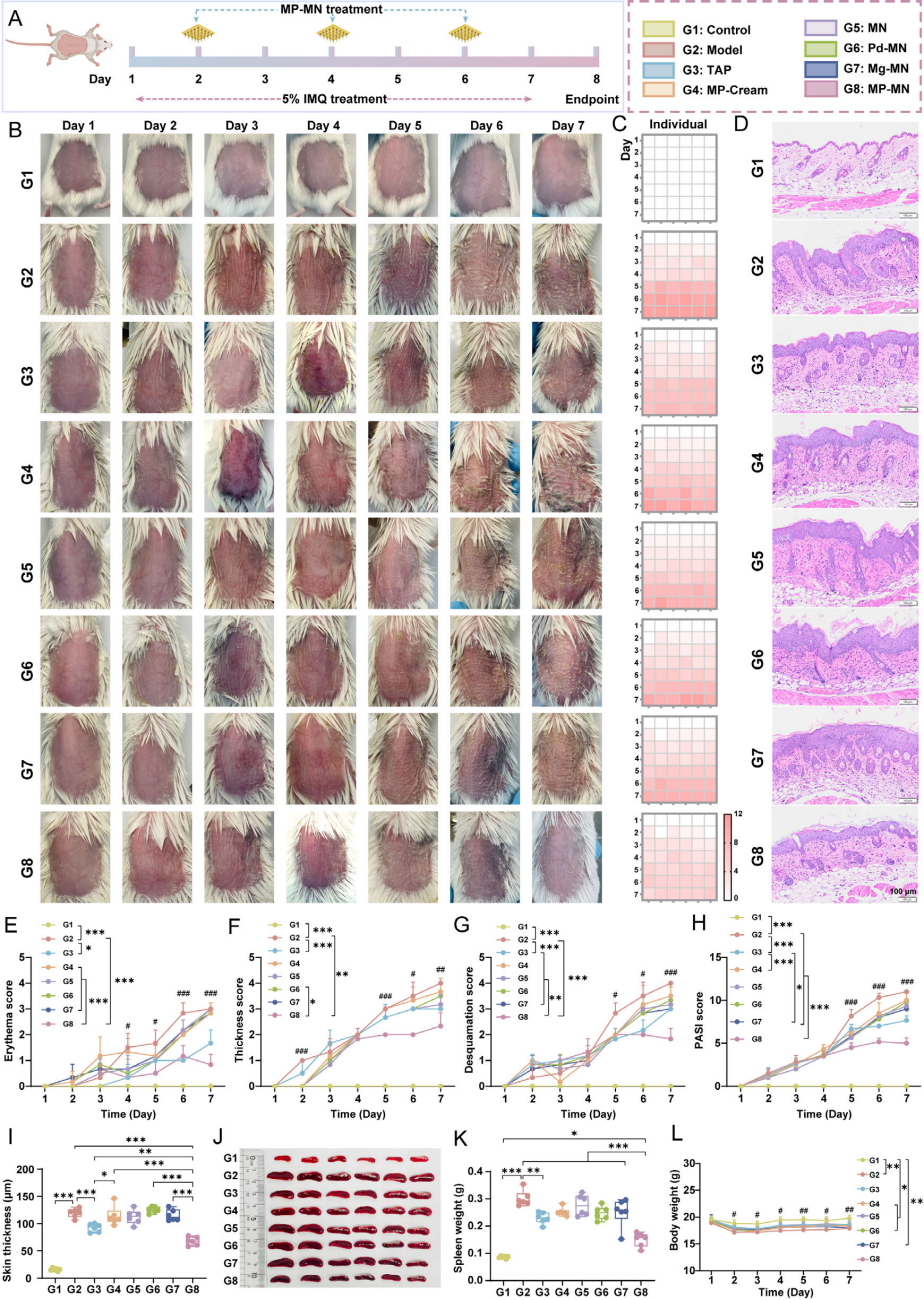
Therapeutic evaluation of MP‐MN in IMQ‐induced psoriatic mouse model. (A) Schematic diagram of MP‐MN therapeutic procedure in psoriasis mice. (B) Representative images of mouse dorsal skin of various groups from days 1–7. (C) Heatmap of the total PASI score of each individual every day. (D) H&E staining images of mouse dorsal skin tissues (Scale bar: 100 µm). (E–H) PASI score including erythema (E), skin thickness (F), desquamation (G), and total score (H). (I) Statistical analysis of skin thickness after different treatments. (J‐K) Spleen size (J) and weight (K) in different groups at the endpoint. (L) Body weight changes in each group during the experiment. Data were shown as the mean ± SD. For each group, n = 6 independent samples in (E‐L). (I, K) ^*^ *p* < 0.05, ^**^ *p* < 0.01, ^***^ *p* < 0.001. (E–H) ^*^ *p* < 0.05 among all groups on Day 7; ^#^ *p* < 0.05, ^##^ *p* < 0.01, and ^###^ *p* < 0.01 between Model group and MP‐MN group. (L) ^*^ *p* < 0.05 among all groups on Day 7; ^#^ *p* < 0.05 and ^##^ *p* < 0.001 between Control group and Model group.

In the Model group, typical psoriatic characteristics, such as erythema, skin thickening, and desquamation on the dorsal skin of mice, were observed on day 2 after IMQ application and were greatly worsened by the end of day 5 (Figure 4B). At the experimental endpoint, pronounced skin wrinkling and scaling were observed in the Model group (Figure 4B). The dorsal skin of mice in the MN, MP‐Cream, Mg‐MN, and Pd‐MN groups also exhibited notable wrinkle formation and scaling. Compared with the Model group, the MP‐MN group displayed only mild scaling and substantially reduced skin thickness, indicating alleviation of psoriatic symptoms (Figure 4B). To more accurately assess the progression of psoriasis‐like lesions, the psoriasis area and severity index (PASI) was scored. As shown in Figure 4C, the PASI scores in the Model group increased during the experimental period. There were no significant differences in total PASI scores among all groups during days 1–4 (Figure 4C). After treatment initiation, the MP‐MN group exhibited a marked decrease in PASI score compared with the Model group on day 5 (Figure 4E–H). During days 6–7, the PASI scores in the Model group reached 10–11, while those in the MN, Mg‐MN, Pd‐MN, and MP‐Cream groups rose to 8–9, with the TAP group scoring slightly lower (Figure 4E–H). Notably, the PASI score in the MP‐MN group began to decline on day 5, showed a slight rebound on day 6, and decreased again on day 7 (the total PASI scores: 4–6), revealing an overall reversal of disease progression (Figure 4H). MP‐MN demonstrated superior efficacy compared with MP‐Cream, which could be attributed to the ability of the microneedle system to effectively penetrate the thickened stratum corneum and deliver H_2_ deep into the skin. The PASI results showed that MN and Pd‐MN treatments hardly had a therapeutic effect on psoriatic‐like skin lesions in mice, as neither formulation was capable of generating H_2_. Mg‐MN treatment also presented a similarly ineffective outcome, presumably due to the passivation of the Mg powder surface, which resulted in insufficient H_2_ generation kinetics. Hematoxylin and eosin (H&E) staining of skin sections revealed that MP‐MN treatment remarkably reduced epidermal thickening on the dorsal skin of mice in comparison with the Model group (Figure 4D,I). In addition, whereas IMQ‐induced mice developed obvious splenomegaly, the spleen size and weight in the MP‐MN group were notably smaller and lighter (Figure 4J,K), preliminarily confirming that MP‐MN not only alleviated psoriatic skin progression but also mitigated the associated systemic immune response. The specific immune response and related mechanism caused by MP‐MN were worth evaluating in the following part.

Body weight in all treated groups remained stable with a normal fluctuation curve throughout the study, as preliminary evidence of the biosafety of the formulations (Figure 4L). To further evaluate the biosafety of MP‑MN and the other formulations, serum and whole blood were collected from healthy mice after administration. Serum biochemical markers, including alanine aminotransferase (ALT), aspartate aminotransferase (AST), creatinine (CREA), blood urea nitrogen (BUN), and magnesium ion levels, remained within normal ranges (Figure S35A–E). These markers indicated that hepatic and renal function remained normal and that systemic magnesium homeostasis was well maintained under MP‑MN treatment. Consistently, the absence of abnormalities in blood routine parameters confirmed the favorable biocompatibility of MP‑MN (Figure S35F–M). Histological examination of major organs displayed intact functional structures, including cardiomyocytes in the heart, hepatic sinusoids in the liver, alveoli in the lung, splenic red and white pulp in the spleen, and glomeruli in the kidney (Figure S36). Given the potential safety concerns associated with metallic materials, Mg and Pd levels in mouse skin and major organs were quantified using ICP and ICP‐MS after topical application of MP‐MN. There were no significant differences of Mg and Pd levels in these tissues between the control group and the treatment group (Figure S37), consistent with the absence of tissue damage observed by H&E staining. Collectively, topical application of MP‑MN to the skin did not induce obvious systemic toxicity in mice, underscoring a key advantage of the microneedle delivery system.

To investigate the long‐term therapeutic efficacy of MP‐MN, a mouse model of recurrent psoriasis was established (Figure S38A). Mice underwent 7 days of initial IMQ induction and treatment, followed by a 7‐day rest period and a 7‐day rechallenge with a lower dose of IMQ. During the initial stimulation, the PASI score in the IMQ (Model) group increased to approximately 11, accompanied by pronounced erythema, skin thickening, and scaling (Figure S38B–D). In contrast, the MP‐MN group had a PASI score of approximately 5 and milder skin lesions (Figure S38B–D). Skin symptoms gradually subsided in all groups during the rest period (Figure S38B). Following re‐induction, erythema, skin thickening, and PASI scores increased in the IMQ and TAP groups, whereas relapse was markedly attenuated in the MP‐MN group (Figure S38B–D). At the peak of relapse (day 21), the PASI score in the MP‐MN group was 1.17 ± 0.41, 84.09% lower than that in the IMQ group (7.33 ± 0.52) (Figure S38D). Body weight remained stable across all groups throughout the experiment (Figure S38E). Moreover, MP‐MN reduced spleen enlargement, as reflected by a lower spleen weight than that in the IMQ group (Figure S38F,G). Together, these findings indicated that MP‐MN effectively attenuated psoriasis‐like relapse.

Although the MP‐MN system showed promising efficacy in mice, several challenges remain for clinical translation. Mouse and human skin differ in thickness, mechanical properties, and lesion characteristics. Human psoriatic plaques are thicker and stiffer, and their distinct dermal composition and interstitial fluid dynamics may alter the diffusion depth and coverage area of H_2_, potentially requiring longer microneedles and higher insertion forces. The DLP‐based microneedles fabrication enables rapid customization of needle geometry. Nevertheless, further validation using skin organoids, ex vivo human skin, large animal models, and eventually clinical trials are necessary to assess penetration efficiency, H_2_ dosing and distribution, long‐term safety, and controllable production of MP‐MN. These studies will be essential to translate the current proof‐of‐concept into a clinically viable therapy.

### 2.5 Local H_2_ Evoked Antioxidant Defense and Broke the Inflammatory Loop

Based on the excellent in vitro and in vivo therapeutic efficiency of MP‐MN, skin tissues from psoriatic mice were subjected to a series of analyses for further elucidating the underlying mechanism. Ki67, a marker of keratinocyte hyperproliferation, was assessed by immunofluorescence staining. The Model group exhibited a marked increase in Ki67 expression, whereas MP‐MN treatment substantially reduced the Ki67 signal, as reflected by diminished green fluorescence (Figure 5A,B). Thus, MP‐MN effectively suppressed epidermal hyperproliferation, consistent with the observed improvement in PASI scores.

**FIGURE 5:**
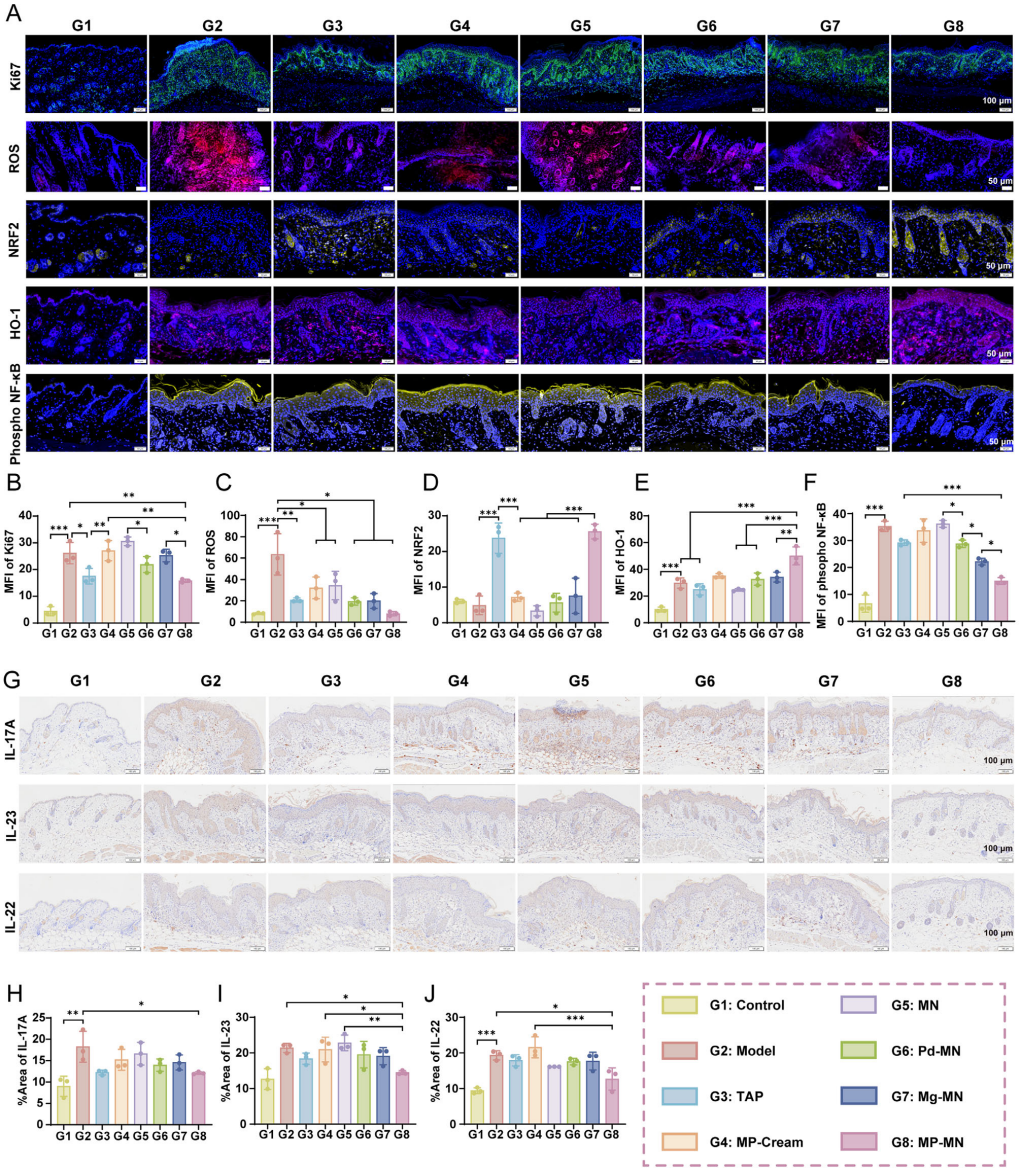
Mechanism of MP‑MN‑mediated H_2_ therapy in ameliorating psoriasis progression. (A) Immunofluorescence staining images for Ki67, ROS, NRF2, HO‐1, and phosphorylated (phospho) NF‐κB of psoriatic skin in mice (Scale bar: 100 µm; Scale bar: 50 µm). (B‐F) Statistical analysis of mean fluorescence intensity (MFI) for Ki67 (B), ROS (C), NRF2 (D), HO‐1 (E), and phospho NF‐κB (F). (G) Immunohistochemical staining images of IL‐17A, IL‐23, and IL‐22 of psoriatic skin in mice (Scale bar: 100 µm). (I‐J) Statistical analysis of signal for IL‐17A (H), IL‐23 (I), and IL‐22 (J). Data were shown as the mean ± SD. For each group, *n* = 3 independent samples in (B‐F, H‐J). ^*^ *p* < 0.05, ^**^ *p* < 0.01, ^***^ *p* < 0.001.

Immunofluorescence staining of ROS in lesional skin revealed that, compared with normal mouse skin, the Model group exhibited a pronounced increase in ROS expression, with remarkably enhanced intensity and expanded distribution of the red fluorescence signal (Figure 5A,C). MP‐MN treatment significantly attenuated oxidative stress in the lesional skin, with the mean fluorescence intensity (MFI) of ROS in the MP‐MN group being reduced by a factor of 7.95 relative to the Model group (Figure 5A,C). In contrast, although some improvement was observed, the TAP, MP‐Cream, blank MN, Pd‐MN, and Mg‐MN groups did not achieve complete restoration of oxidative stress homeostasis (Figure 5A,C). As evidence for the activation of the KEAP1–NRF2 antioxidant signaling pathway, MP‐MN treatment obviously upregulated NRF2 expression in the skin, which was increased 5.24‐fold compared with the Model group (Figure 5A,D). Besides, the HO‐1 fluorescence signal intensity in the MP‐MN group was elevated 1.68‐fold, 2.01‐fold, and 1.46‐fold relative to the Model, TAP, and Mg‐MN groups, respectively (Figure 5A,E). Collectively, through local delivery of sufficient H_2_, MP‐MN effectively contributed to diminished ROS levels and activated the endogenous antioxidant pathway.

In chronic inflammation, persistent phosphorylation of NF‐κB at the S536 site drives the continuous secretion of pro‐inflammatory cytokines, perpetuating a severe inflammatory microenvironment [39, 40]. The fluorescence intensity of phosphorylated NF‐κB (yellow signal) in the Mg‐MN group was reduced to approximately 62.9% of that in the Model group (Figure 5A,F), suggesting that Mg^2+^ potentially contributed to a slight anti‐inflammatory effect in ameliorating skin lesions. Notably, the phosphorylated NF‐κB level in the MP‐MN group was markedly decreased, with signal intensity reduced by a factor of 2.35 relative to the Model group (Figure 5A,F). Furthermore, MP‐MN treatment suppressed the secretion of IL‐17A, IL‐23, IL‐22, TNF‐α, and IL‐6 in the skin tissue of psoriatic mice (Figure 5G–J and Figure S39). These findings elucidated that local application of MP‐MN alleviated psoriatic symptoms by disrupting the oxidative stress–inflammation feedback loop, resulting in reduced systemic inflammation.

### 2.6 The Immunological Mechanism of MP‐MN

It was reported that IMQ stimulation could evoke spleen enlargement, which was associated with the release of pro‐inflammatory cytokines [41]. Considering the differences in spleen weight among the various groups, the immune cell composition of the spleen should be analyzed. In addition, as a hallmark of psoriasis, immune dysregulation was characterized by an imbalance between Th17 and Treg cells, with upregulation of the Th17 subset and a concomitant reduction in the proportion of immunologic tolerance Treg cells [42]. Representative flow cytometry images showed that the Treg subset displayed a downward trend in the Model group, while the proportion of the Th17 subset exhibited a significant upward trend (Figure 6A,B). This suggested that there was an evident imbalance in immune cells within the spleen under IMQ stimulation. After treatment, the Treg subset (FoxP3^+^) in CD4^+^ cells was 9.83% in the Model group versus 19.64% in the MP‐MN group (Figure 6A,E and Figure S40). The immune regulation of the MP‐MN group was accompanied by a significant reduction in the Th17 subset (Figure 6B,F and Figure S41). After treatment, MP‐MN groups presented a downregulation of the Th17/Treg ratio in the spleen tissue, with a reduction of approximately 78.0% compared with the Model group (Figure 6G). With respect to innate immunity, the spleen of MP‐MN‐treated mice also showed a distinct decrease in neutrophil proportion, which dropped to 4.41%, compared with 12.11% in the Model group (Figure 6C,H and Figure S42). Concurrently, the proportion of anti‐inflammatory M2 macrophages in the MP‐MN group was remarkably elevated, reaching 3.44 times that of the Model group (Figure 6D,I and Figure S43), along with a decreased M1/M2 ratio (Figure 6J and Figure S44). In summary, these discoveries highlighted the pivotal role of MP‐MN in restoring immune cell homeostasis in psoriasis.

**FIGURE 6:**
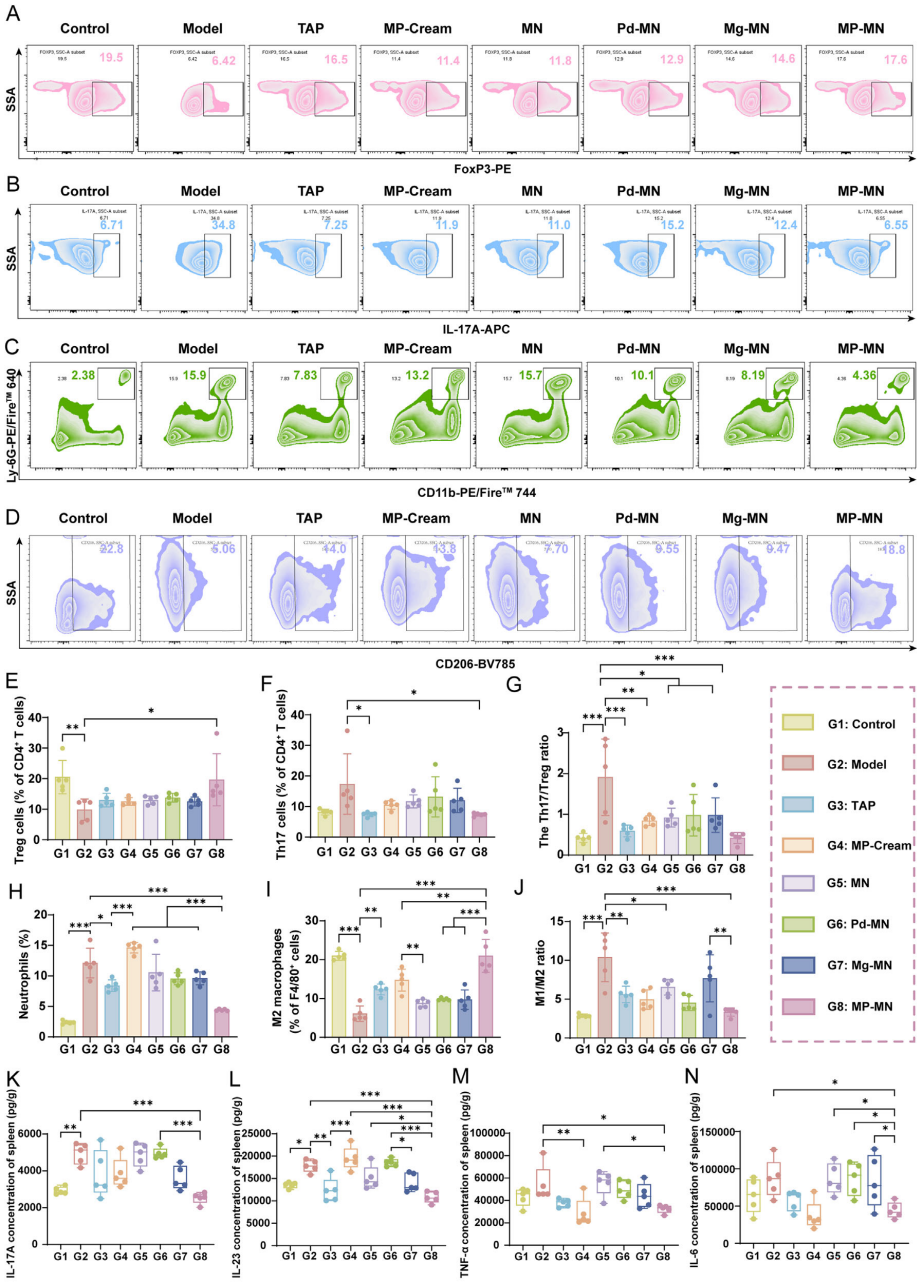
Analysis of immune regulation of MP‐MN in the spleen. (A‐D) Representative flow cytometry of Treg (A), Th17 cells (B), neutrophils (C), and M2 macrophages (D) in various groups. (E‐J) Quantification of Treg (E), Th17 cells (F), Th17 cells /Treg ratio (G), neutrophils (H), M2 macrophages (I), and M1/M2 ratio (J) in the spleens by flow cytometry. The expression levels of IL‐17A (K), IL‐23 (L), TNF‐α (M), and IL‐6 (N) in the spleen tissue. Data were shown as the mean ± SD. For each group, *n* = 5 independent samples in (E‐N). ^*^ *p* < 0.05, ^**^ *p* < 0.01, ^***^ *p* < 0.001.

IL‐23 and IL‐17A are essential pro‐inflammatory cytokines in the pathogenesis of psoriasis. IL‐23 drives the differentiation, activation, and expansion of Th17 cells, which in turn produce large amounts of IL‐17A [11]. Subsequently, IL‐17A acts on innate immune cells to trigger the release of TNF‐α and IL‐6 [10, 43]. ELISA analysis revealed that the levels of IL‐17A, IL‐23, TNF‐α, and IL‐6 in the spleen tissue of MP‐MN‐treated mice were obviously reduced (Figure 6K–N), further validating that MP‐MN effectively attenuated systemic inflammation associated with psoriasis.

### 2.7 Transcriptomic Analysis

To further explore how MP‐MN modulated the psoriatic transcriptome, transcriptomic profiling of psoriatic‐like skin tissues was performed and analyzed through differentially expressed genes (DEGs) between the MP‐MN treatment and Model groups. The volcano map of differential analysis displayed that 2916 DEGs were identified between the MP‐MN and Model groups, including 1435 upregulated and 1481 downregulated genes (Figure 7A). As a key chemokine in the inflammatory network of psoriasis, *cxcl13* binds to its receptor CXCR5 to mediate immune cell chemotaxis, recruiting immune cells to the skin and exacerbating local inflammation [44]. The downregulation of *cxcl13* by MP‐MN treatment indicated that MP‐MN participated in reversing psoriasis progression. The heatmap revealed distinct gene expression differences, reflecting extensive transcriptional alterations upon treatment (Figure 7B). Reactome analysis revealed enrichment of DEGs in immune system, metabolism, and signal transduction pathways (Figure 7C).

**FIGURE 7:**
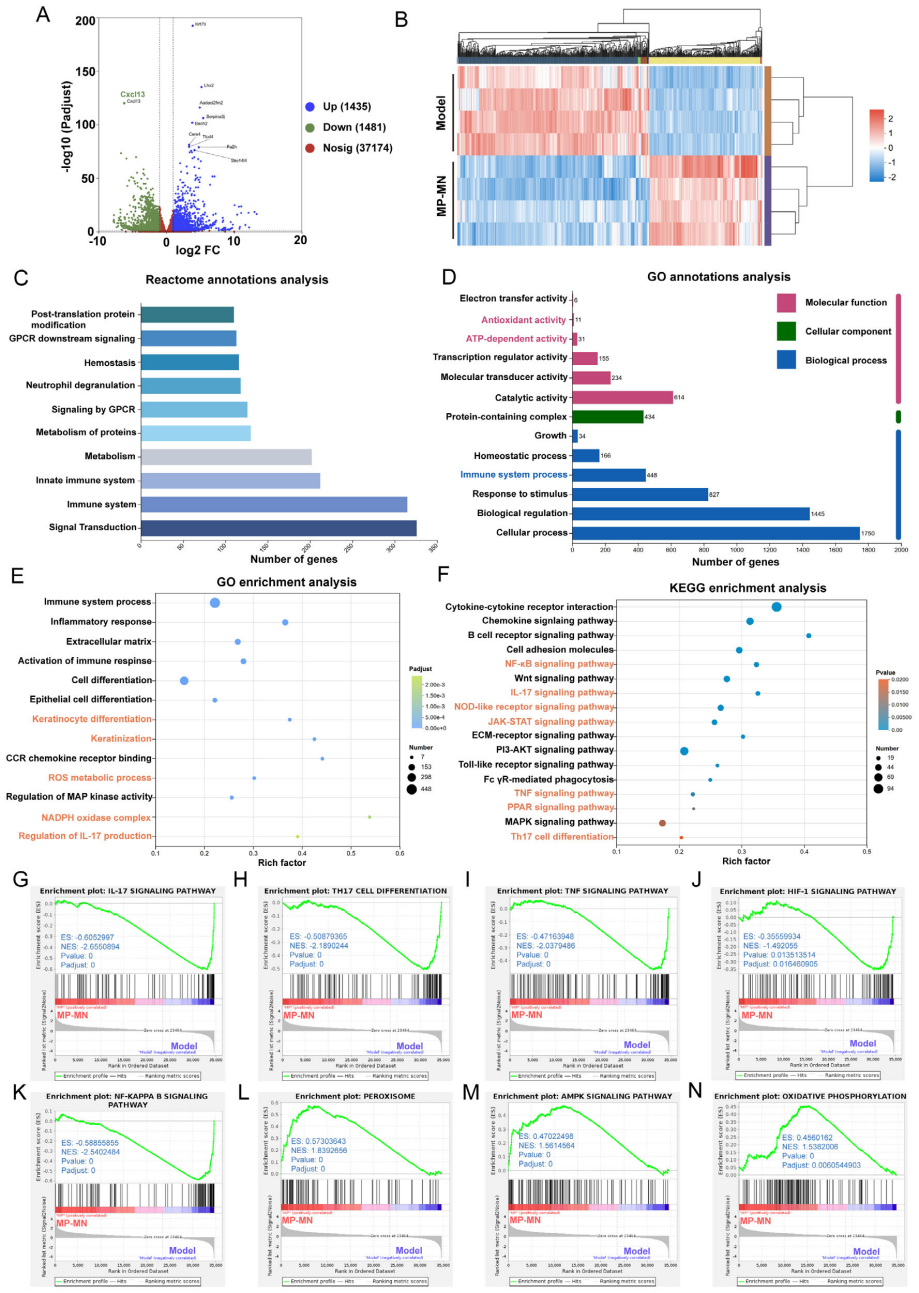
Transcriptomic analysis of psoriasis skin after H_2_ therapy treatment. (A) Volcano plot displaying DEGs between the Model and MP‐MN groups. (B) Heatmap showing DEGs between the Model and MP‐MN groups. (C) Reactome annotation analysis after MP‐MN therapy. (D) GO annotation analysis of the DEGs in the Model and MP‐MN groups. (E) GO enrichment analysis of DEGs between the Model and MP‐MN groups. (F) KEGG enrichment analysis of the DEGs displays enriched pathways. (G‐N) GSEA analysis of the IL‐17 signaling pathway (G), Th17 cell differentiation (H), TNF signaling pathway (I), HIF‐1 signaling pathway (J), NF‐κB signaling pathway (K), peroxisome (L), AMPK signaling pathway (M), and oxidative phosphorylation (N). For each group, *n* = 4 independent samples.

To determine the effects of MP‐MN on the biological functions and signaling pathways of oxidative stress‐inflammation‐immunity in psoriasis, Gene Ontology (GO) analysis and Kyoto Encyclopedia of Genes and Genomes (KEGG) analysis were conducted. The GO annotation analysis showed that DEGs were mainly involved in antioxidant activity, immune system process, and ATP‐dependent activity (Figure 7D). The GO enrichment analysis demonstrated that DEGs were enriched in biological processes such as keratinocyte differentiation, keratinization, regulation of IL‐17 production, ROS metabolic process, and nicotinamide adenine dinucleotide phosphate (NADPH) oxidase complex (Figure 7E). These results suggested that the therapeutic effect caused by MP‐MN was associated with reversing the aberrant proliferation and differentiation state of keratinocytes, while preliminarily demonstrating that MP‐MN affected inflammation and oxidative stress‐related genes.

KEGG pathway analysis revealed that DEGs were significantly enriched in the Th17 cell differentiation, IL‐17, TNF, NF‐κB, Janus kinase‐signal transducer and activator of transcription (JAK‐STAT), peroxisome proliferator‐activated receptor (PPAR), and nucleotide‐binding oligomerization domain (NOD)‐like receptor signaling pathways (Figure 7F). At the immune level, both KEGG and Gene Set Enrichment Analysis (GSEA) consistently demonstrated the enrichment and downregulation of Th17 cell differentiation, IL‐17, and TNF signaling pathways in the MP‐MN group (Figure 7F–I). Notably, MP‐MN treatment was associated with downregulation of HIF‐1 signaling, which is consistent with a reduced glycolytic energy supply on which Th17 cells depend, as HIF‐1α acts as a co‐activator of RORγt‐driven Th17 differentiation [43] (Figure 7J). These transcriptional signatures suggested that MP‐MN might block Th17 cell differentiation and effector function at the transcriptomic level. NF‐κB, JAK‐STAT, and NOD‐like receptor signaling pathways have been reported to engage in reciprocal crosstalk with IL‐17, Th17, and TNF signaling, forming an upstream–downstream regulatory loop [8, 45, 46, 47]. Both KEGG and GSEA results showed that NF‐κB, JAK‐STAT, and NOD‐like receptor signaling pathways were significantly enriched and suppressed, thereby intercepting core inflammatory cytokine signaling (Figure 7F,K and Figure S45A,B). Concurrently, since PPAR signaling pathway was upregulated, MP‐MN inhibited NF‐κB phosphorylation in keratinocytes (Figure S45C), which might reduce neutrophil recruitment and ameliorate the psoriatic inflammatory microenvironment [48].

At the oxidative stress and metabolic level, GSEA presented upregulation of peroxisome, AMPK signaling pathway, and oxidative phosphorylation in the MP‐MN group (Figure 7L–N). These changes, together with the downregulation of HIF‐1 signaling and the enrichment of ROS metabolic process and NADPH oxidase complex identified in GO analysis, suggested that MP‐MN might not only suppress inflammation‐driven ROS production but also enhance the intrinsic oxidative clearance capacity of cells. Collectively, these transcriptomic signatures were consistent with a shift from inflammatory aerobic glycolysis toward oxidative phosphorylation in psoriatic skin tissue. In summary, MP‐MN treatment disrupted the vicious cycle of keratinocyte hyperproliferation, oxidative stress, and immune cell‐driven inflammation at the transcriptomic level, offering promise for psoriasis therapy.

## 3 Conclusion

In summary, a magnesium‐based micro‐galvanic cell (MP) was developed for psoriasis treatment. MP approached and reacted with the interstitial fluid of the skin through spontaneous electrochemical corrosion to release H_2_. H_2_ selectively scavenged ROS and •OH, drove the dissociation of NRF2 and KEAP1, thereby promoting nuclear translocation of NRF2 and activating the downstream antioxidant enzyme HO‐1. The dual outcomes effectively restored the oxidative stress homeostasis of psoriatic skin and suppressed the excessive proliferation of keratinocytes, leading to diminished inflammatory factors. For transdermal delivery of H_2_, DLP‐based 3D printing technology with high controllability and consistency was utilized to develop MP‐MN. In vivo results revealed that MP‐MN ensured local delivery of H_2_ to skin lesions in the IMQ‐induced psoriasis mouse model, which effectively decreased the PASI score and restored skin thickness. In addition, MP‐MN reduced the level of oxidative stress, decreased pro‐inflammatory factors in skin and spleen, and reversed Th17/Treg imbalance. In conclusion, 3D printing‐assisted microneedle fabrication facilitated the preservation of the H_2_‐generating MP and the transdermal delivery of H_2_, providing a basis for further investigation of hydrogen‐based therapy for psoriasis.

Redox‐based therapies for psoriasis include both antioxidant and pro‐oxidant strategies. Cold atmospheric plasma (CAP)‐based therapies deliver exogenous RONS to induce keratinocyte apoptosis, representing an oxidative stress‐inducing strategy [49, 50]. CAP RONS have shown efficacy by inducing keratinocyte apoptosis and restoring differentiation [51, 52]. In contrast, MP‐MN scavenges cytotoxic ROS via H_2_, preserving physiological redox signaling and activating the NRF2–KEAP1–HO‐1 axis. Different from CAP, which relies on dose‐dependent oxidative stress and spatial targeting, H_2_ offers intrinsic molecular selectivity and a wide safety therapeutic window. The MP‐MN platform further enables sustained, minimally invasive H_2_ delivery, overcoming the delivery bottleneck of H_2_ therapy. Thus, these two strategies are complementary, with H_2_‐based antioxidant therapy may suit for long‐term, safe management of psoriasis.

## 4 Materials and Methods

### 4.1 Materials and Regents

Magnesium (Mg with average particle size of 40–50 µm) was purchased from China New Metal Materials Technology Co. Ltd. (Beijing, China). Sodium tetrachloropalladate (Na_2_PdCl_4_), Salicylic acid, and FeSO_4_⚫7H_2_O were acquired from Shanghai Aladdin Biochemical Technology Co., Ltd. Isopropanol (IPA) and imiquimod (IMQ) were obtained from Shanghai Adamas Reagent Co., Ltd. Gelatin methacryloyl 90 (GelMA 90), Polyethylene glycol diacrylate (PEGDA‐600) hydrogel, lithium phenyl (2,4,6‐trimethylbenzoyl) phosphinate (LAP), and photoabsorber were purchased from Engineering for Life (Suzhou, China). DMEM medium and fetal bovine serum (FBS) were obtained from Gibco Life Sciences (Grand Island, USA). Penicillin‐streptomycin solution and CCK‐8 assay kits were acquired from Meilunbio Technology Co., Ltd. (Dalian, China). Hoechst 33342, ROS assay kit, ABTS assay kit, DHE assay kit, apoptosis kit, MDA kit, CAT kit, nuclear protein extraction kit, and anti‐rabbit IgG HRP‐linked antibody were acquired from Beyotime Biotechnology Co., Ltd. (Shanghai, China). Recombinant human TNF‐α and IL‐17a polyclonal antibodies were purchased from Proteintech Group, Inc. (Wuhan, China). NRF2 rabbit monoclonal antibody, phospho‐NF‐κB p65 (Ser536) antibody, NF‐kB p65 rabbit monoclonal antibody, IκBα rabbit antibody, and KEAP1 rabbit monoclonal antibody were obtained from Cell Signaling Technology (CST, Massachusetts, USA). TNF‐α rabbit polyclonal antibody, β‐actin polyclonal antibody, IL‐6 rabbit polyclonal antibody, IL‐22 rabbit polyclonal antibody, Histone H3 rabbit monoclonal antibody was acquired from ABclonal Technology Co., Ltd. (Wuhan, China). β‐tubulin polyclonal antibody was purchased from Invitrogen Co., Ltd. (California, USA). Human IL‐6 precoated ELISA kit and human IL‐1β precoated ELISA kit were acquired from Dakewe Biotech Co., Ltd. (Shenzhen, China). ML385 was purchased from MedChemExpress (Monmouth Junction, NJ, USA). The human CCL20 ELISA kit was obtained from Shanghai Acmec Biochemical Technology Co., Ltd. The mouse IL‐17A ELISA kits, the mouse IL‐23 ELISA kits, the mouse IL‐6 ELISA kits, and mouse TNF‐α ELISA kits were purchased from Jianglai Biotechnology Co., Ltd. (Shanghai, China). 70 µm cell strainers and methylene blue (MB) were purchased from Solarbio Science &Technology Co., Ltd. (Beijing, China). Red blood cell lysis buffer and IL‐23 rabbit polyclonal antibody were obtained from Absin Bioscience Inc. (Shanghai, China). Fixation/permeabilization concentrate, FVD455UV, permeabilization buffer, anti‐mouse CD16/32, BV510 anti‐mouse CD45, Spark Blue^TM^ 574 anti‐mouse CD3, Spark red 718 anti‐mouse CD86, BV570 anti‐mouse CD4, PE/Fire^TM^744 anti‐mouse CD11b, APC/Fire^TM^810 anti‐mouse F4/80, APC anti‐mouse IL‐17A, PE/Fire^TM^640 anti‐mouse Ly‐6G, PE anti‐mouse FoxP3, BV785 anti‐mouse CD206, and cell activation cocktail (with Brefeldin A) were purchased from BioLegend, Inc. (San Diego, USA).

### 4.2 Cell Culture

Human immortalized keratinocytes (HaCaT) cell line was purchased from Shanghai Jiwei Biological Technology Co., Ltd. The cell line was cultured in DMEM medium supplemented with 10% FBS and 1% penicillin‐streptomycin solution. HaCaT cells were cultured with 5% CO_2_ at 37°C.

### 4.3 Animals

Female BALB/c mice (8 weeks old) were purchased from Zhejiang Vital River Laboratory Animal Technology Co., Ltd. All animal experiments were conducted in accordance with protocols approved by the Institutional Animal Care and Use Committee (IACUC) of Lingang Laboratory (LGLSP‐2023‐010‐2). Mice were maintained under specific pathogen‐free (SPF) conditions on a 12 h light/dark cycle.

### 4.4 Synthesis of Mg‐Pd Microgalvanic Cells (MP)

MP were synthesized based on a displacement reaction. To exclude moisture and oxygen, 10 mg of Mg powder was subjected to evacuation/N_2_ refilling cycles. Subsequently, 1 mL of IPA was added, and the mixture was stirred at 1000 rpm for 5 min. Na_2_PdCl_4_ solution in IPA (2 mg/mL) was added to the Mg suspension under stirring at 1000 rpm and at room temperature. To optimize the MP parameters, reactions were carried out at various Mg: Pd mass ratios (10:1, 8:1, 4:1, 2:1, 1:1, and 1:2) and for different reaction times (1, 2, 4, 6, 8 h). The precipitate was collected and washed twice with IPA. Finally, the product was dried under vacuum at 40°C for 4 h to obtain the MP.

The morphology and elemental mapping of MP synthesized under different conditions were observed by scanning electron microscope (SEM) and energy dispersive spectrometer (EDS) (Gemini SEM 360, Zeiss, Germany). The H_2_ generation content of MP powders prepared under different immersion time and reaction ratio (Mg: Pd, w/w) conditions was monitored by a dissolved hydrogen meter (DH30L, Clean Instrument, China).

### 4.5 Characterization of MP

The morphology and elemental mapping of the MP were characterized by SEM and EDS (Gemini SEM 360, Zeiss, Germany). The chemical valence of MP was examined by X‐ray photoelectron spectroscopy (XPS, ESCALAB Xi+, Thermo Scientific, USA). The chemical composition and phase analysis of MP were detected by X‐ray diffractometer (XRD, D8 DaVinci diffractometer, Bruker, Germany). Electric current, Tafel curves, corrosion potential, and electrochemical impedance spectroscopy (EIS) of MP or Mg powders were detected by an electrochemical workstation (CHI660E, CH Instruments).

### 4.6 Measurement of H_2_ Generation In Vitro

Since the blue MB solution could be reduced to colorless by hydrogen, the MB probe was used to qualitatively measure the H_2_ generation of MP or Mg powders in PBS solution. The UV absorbance spectra from 400–900 nm of samples were scanned by a multimode microplate reader (Spark, Tecan, Switzerland). The decreasing trend at 664 nm of samples at different times (1, 5, 10, and 20 min) was monitored. Meanwhile, absorption spectra of the MB and MB solution with Mg or MP added after 20 min were compared. In addition, quantitative analysis of H_2_ generation from the Mg and MP in PBS was conducted by gas chromatography (GC 8860, Agilent, USA).

### 4.7 In Vitro Free Radical Scavenging Assay

ABTS radical scavenging assay: The ABTS assay solution was obtained by reacting with an equal volume of ABTS solution and oxidant solution in the dark at room temperature for 12 h and was diluted 35‐fold with PBS before use. The ABTS assay solution (1 mg/mL) containing MP or Mg powders was reacted for 2, 5, and 10 min, respectively, and the wavelength of UV absorption was scanned (500–900 nm). The color change of the solution was photographed. Moreover, MP or Mg powders were added to ABTS assay solution for 5 min to achieve different mass‐to‐volume ratios (200, 500, 1000, and 2000 µg/mL). The UV absorbance of the above solution at 734 nm was measured by a multimode microplate reader (Spark, Tecan, Switzerland).

•OH scavenging test: FeSO_4_·7H_2_O (33.36 mg) was dissolved in pure water, mixed with 136 µL of 3% H_2_O_2_, and then diluted to 20 mL with pure water. Salicylic acid (16.57 mg) was dissolved in 20 mL of absolute ethanol. The two solutions were immediately combined, turning dark purple, and subsequently diluted with an equal volume of pure water to obtain the •OH assay solution (FeSO_4_·7H_2_O, salicylic acid, and H_2_O_2_ were all 1.5 mM). Then, Mg powders or MP were added to achieve different mass‐to‐volume ratios (0, 200, 400, 800, 1000, and 2000 µg/mL). After 10 min of reaction, the MP and Mg powders were removed by centrifugation (8000 rpm, 5 min). The absorbance at 510 nm was detected, and the spectrum was scanned at the wavelength of 400–900 nm.

Briefly, different MP concentrations (100, 200, 400, 800, 1000, and 2000 µg/mL) was incubated with 4 mM H_2_O_2_ for 10 min. After removal of the residual MP powders, the remaining supernatant was mixed with Ti(SO_4_)_2_ (10 mg/mL) to precipitate the yellowish peroxotitanium complex. After 15 min, the precipitate was collected by centrifugation at 12000 g for 15 min and subsequently dissolved in 2 M H_2_SO_4_. Finally, the H_2_O_2_ scavenging capacity of MP was evaluated by measuring the absorbance at 412 nm using a multimode microplate reader (Spark, Tecan, Switzerland).

For •OH and ^1^O_2_ detection, TiO_2_ (10 mg/mL) was used to generate reactive oxygen species in a 1 mL aqueous reaction mixture. DMPO and TEMP were used to trap •OH and ^1^O_2_, respectively, each at a concentration of 400 mM. For O_2_^−^ detection, C_3_N_4_ (10 mg/mL) was used in a 1 mL methanolic reaction mixture containing 400 mM DMPO. All reaction mixtures were subjected to photocatalysis for 5 min. Subsequently, 2 mg of Mg or MP was added to each ROS‐containing control reaction mixture. After 20 min of reaction, the supernatants were collected for electron spin resonance (ESR, Bruker EMXnano, Germany) analysis.

### 4.8 Fabrication of 3D‐Printed Microneedle Patches

The preparation of MP powder‐loaded microneedles involved six steps: model design, slicing, photopolymerization‐based printing, washing, drying, and material backfilling. First, a hollow microneedle (HMN) model with pores in tips was designed using 3D‐Max software, featuring quadrangular pyramid‐shaped tips arranged in a 5 × 5 array. The square substrate incorporated two openings for material filling, yielding 23 tips per patch. Subsequently, a composite bioink was prepared by mixing 8% (w/v) GelMA‐90, 2% (w/v) PEGDA‐600, 0.25% (w/v) photoinitiator (LAP), and 0.05% (w/v) water‐soluble photoabsorber. The HMN was fabricated using a DLP‐based projection stereolithography 3D bioprinter (EFL‐BP8601Pro, EFL, China). Before printing, the bioink was equilibrated at 50 °C to maintain flowability. The designed 3D model was imported into the printer software and sliced at a layer thickness of 100 µm. Printing parameters were set as follows: light intensity of 9 mW/cm^2^, peeling speed of 25 mm/min, and a temperature of 40°C for both the platform and resin vat. Meanwhile, UV sterilization mode was activated throughout the printing process. After printing, the patches were immediately rinsed with pre‐warmed 75% ethanol (50°C) to remove residual gel from the cavities, followed by drying at 40°C for 24 h. The resulting blank MNs (denoted as MN) exhibited flat bases, intact cavities, and sharp, well‐defined quadrangular pyramid tips. For drug‐loaded MNs, the hollow cavities were filled with Mg or MP powders, and the base openings were sealed via GelMA gelation, followed by photocuring of the hydrogel at both openings using a portable UV light source, yielding Mg‐MN and MP‐MN. Pd‐MN was prepared by mixing the acid‐dissolution residue of MP with the bioink, followed by DLP‐based printing, photocuring, and drying.

### 4.9 Characterization of 3D‐Printed Microneedle Patches

The real morphology of MP‐MN was recorded using a stereo microscope (Zeiss). The SEM and EDS images (Gemini SEM 360, Zeiss, Germany) was utilized to image the microneedle microgeometry. To investigate the mechanical properties of MP‐MN, force‐displacement measurements were performed using a texture analyzer (Shanghai TengBa Instrument Technology Co., LTD) with a 5 kN load cell. The test parameters were set as follows: the load was zeroed, and the probe moved downward at 0.5 mm/s until a pre‐load of 0.049 N was reached. Subsequently, the displacement was zeroed, and the probe advanced downward at 0.05 mm/s to a relative displacement of 0.8 mm. Individual needle tips were photopolymerized, dried, and then compression‐tested using a texture analyzer to evaluate their mechanical properties at a displacement of 0.8 mm. The Mg content in the supernatant following the reaction of MP‐MN in PBS were determined by inductively coupled plasma (ICP, iCAP7600, Thermo, USA).

The concentration of H_2_ generated from MP‐MN was detected by GC (GC 8860, Agilent, USA). Apply MP‐MN to the mouse skin of mice for 1, 4, 24 and 48 h. The hydrogen content in skin samples was determined by gas chromatography (GC 8860, Agilent, USA) with an injection volume of 1 mL. There were three parallel samples at each time point. To determine whether MP‐MN induced changes in local skin potential, an electrochemical workstation (CS300M, Corrtest, China) was used to monitor the skin potential of anesthetized mice in real time. The dorsal skin was exposed and connected to electrodes, and potential changes were recorded over 25 min with or without application of an MP‐MN patch. The insertion capability of the microneedle patch was evaluated on mouse skin. The status of the micropores on the skin surface was photographed at various time points using a digital device. Furthermore, histological analysis of the skin following patch application confirmed the formation of microchannels through a tissue slice scanner (VS200, Olympus, Japan).

### 4.10 Induction of Cellular Inflammation Model

To establish an inflammatory model of oxidative stress imbalance in HaCaT cells, different concentrations of TNF‐α (0, 5, 10, 20, 40, 50, 80, 100, and 200 ng/mL) were utilized to induce oxidative stress in HaCaT cells. After 12 h or 24 h, DMEM medium containing TNF‐α was removed. HaCaT cells were cultured with fresh DMEM medium with 10% CCK8 for 1.5 h. HaCaT cell viability was measured by UV absorption spectrometer (Infinite 200 PRO, Tecan, Switzerland) at 450 nm.

After the TNF‐α incubation time was determined, the ROS index was used to determine the TNF‐α concentration. The ROS level of HaCaT cells induced by 0, 20, 40, 50, and 80 ng/mL TNF‐α was detected by DCFH‐DA probe. Flow cytometry was utilized to detect the green fluorescence signal representing ROS (NovoCyte D2060R, Agilent, USA).

### 4.11 Cell Viability Assay

To assess the in vitro cell viability in the inflammatory model of HaCaT cells, cells were seeded in 96‐well plates at 5 × 10^3^ cells/well and incubated at 37°C for 8 h. Cells were induced with 40 ng/mL TNF‐α for 24 h. Subsequently, HaCaT cells were incubated with Mg or MP powders at different concentrations of Mg (0, 12.5, 25, 50, 100, 200, 400, 800, and 1000 µg/mL) for 24 h. Additionally, HaCaT cells were incubated with the Pd residues obtained from acid‐digested MP, where the MP corresponded to Mg concentrations of 0, 12.5, 25, 50, 100, 200, 400, 800, and 1000 µg/mL. Then cells were washed twice with PBS before adding CCK‐8 reagent. Cells were incubated with fresh DMEM medium containing 10% CCK‐8 for 1.5 h. The cell viabilities were determined by UV absorption spectrometer (Infinite 200 PRO, Tecan, Switzerland) at 450 nm.

For normal HaCaT cells, cells were seeded in 96‐well plates and cultured overnight. Then, normal HaCaT cells were incubated with Mg powders or MP powders at different concentrations of Mg (0, 25, 50, 100, 200, 400, 800, and 1000 µg/mL), for 24 h. Other procedures were the same as described above.

For the cell‐free control, Mg, Pd, and MP at the indicated concentrations were incubated with 10% CCK‐8 reagent in the absence of cells for 1.5 h at 37°C, and absorbance at 450 nm was measured.

### 4.12 In Vitro Cell Experiments

HaCaT cells were seeded in a 24‐well glass plate (5 × 10^4^ cells/well) and in a 12‐well plate (1 × 10^5^ cells/well) at 37°C for 8 h. All groups (Control, TNF‐α, Pd, Mg, and MP) except the control group were induced by 40 ng/mL TNF‐α for 24 h. The concentration of Mg in the DMEM medium was 100 µg/mL for treatment groups, including Mg powders, Pd (the residue of MP after acid dissolution), or MP powders.

For ROS detection, the cellular supernatant was removed, followed by adding 10 µM DCFH‐DA medium in the dark. After 30 min, the stained HaCaT cells were detected by flow cytometry (NovoCyte D2060R, Agilent, USA) and were observed by confocal laser scanning microscopy (CLSM, Stellaris 5, Leica, Germany).

DHE probe was utilized to measure O_2_^−^ levels. The cellular supernatant was removed, and the cells were washed twice with PBS. DHE staining assay solution (0.5 mL) was added to each well. The cells were incubated at 37°C in the dark for 20 min. The staining solution was discarded, washed twice with PBS, then 0.5 mL 1 × Hoechst 33342 solution was added, and the cells were incubated at 37°C in the dark for 25 min. The cells were washed twice with PBS before detection by CLSM.

To analyze O_2_^−^ levels in mitochondria, the culture medium was removed, followed by adding 200 nM Mito‐Tracker Green to label mitochondria. Subsequently, MitoSO^TM^ Red staining working solution was added and incubated with HaCaT cells at 37°C in the dark for 30 min. Finally, 1 × Hoechst 33342 solution was applied for nuclear staining of HaCaT cells, and the cells were further analyzed by CLSM.

For the antioxidant test, the intracellular total antioxidant levels were also detected by ABTS method. 10 µL of PBS or sample was added to the blank control well and sample detection well, respectively. Then, 200 µL of ABTS assay solution was added to each detection well. The wavelength at 734 nm was determined after 4 min incubation at room temperature.

Intracellular MDA levels were determined using a colorimetric method based on its reaction with thiobarbituric acid (TBA) to yield a red product. Cells were lysed, and the lysates were collected and normalized for protein content via the BCA assay. Then, 200 µL of MDA working solution (containing TBA and an antioxidant mixture) was added to each tube containing 100 µL of samples. The mixtures were heated in a boiling water bath (100 °C) for 15 min, cooled to room temperature, and centrifuged at 1000 g for 10 min. Finally, the absorbance of the supernatant (200 µL) was measured at 532 nm.

For the CAT enzyme activity, HaCaT cells were lysed, and the resulting lysates were collected for protein quantification. The lysates were then mixed with 250 mM H_2_O_2_ and incubated at 25°C for 5 min, after which a catalase stop solution was added to terminate the reaction. The stopped mixture was diluted 5‑fold with catalase assay buffer. Subsequently, 10 µL of the above diluted solution was combined with 200 µL of chromogenic working solution, incubated at 25°C for 25 min, and the absorbance was recorded at 520 nm.

In addition, the cellular supernatant was collected to measure the content of IL‐6, IL‐1β, and CCL20 secreted by HaCaT cells according to ELISA detection kits. A cell apoptosis assay was performed according to an apoptosis kit.

### 4.13 Western Blot Assay

HaCaT cells were seeded in a 6‐well glass plate at 5 × 10^5^ cells/well at 37°C for 8 h. All groups (Control, TNF‐α, Pd, Mg, and MP) except the control group were induced with 40 ng/mL TNF‐α for 24 h. The concentration of Mg in the DMEM medium was 100 µg/mL for treatment groups, including Mg powders, Pd (the residue of MP after acid dissolution), or MP powders. The total cellular protein from HaCaT cells was extracted with lysis buffer and was quantified by the BSA method. Nuclear protein was extracted using a nuclear protein extraction kit to observe the translocation and expression of NRF2.

Protein samples were separated via 4%–20% SDS–PAGE gel and transferred onto PVDF membranes (0.45 µm). The membranes were blocked for 10 min and then incubated for 16 h at 4°C with primary antibodies against KEAP1, phospho‐NF‐κB (Ser536), NRF2, HO‐1, Histone H3, β‐Tubulin, and β‐Actin. After washing with TBST, the membranes were incubated with rabbit HRP‐conjugated secondary antibodies for 40 min at room temperature. Protein bands were visualized by chemiluminescence with Touch Imager Pro imaging system (e‐Blot, China).

### 4.14 Evaluation of the Individual Effects of H_2_ and Mg^2+^

To assess the individual effects of H_2_ and Mg^2+^, TNF‐α‐induced HaCaT cells were treated with MgCl_2_ (100 µg/mL) or the products remaining after MP depletion (MP‐After) for 6 h. Cells in the H_2_ group were cultured in a hydrogen‐containing incubator for 6 h. Intracellular ROS levels were then assessed by DCFH‐DA probe, confocal microscopy, and flow cytometry. Refer to Section 4.12 for experimental procedures.

### 4.15 The Role of NRF2 in Antioxidant Activity

To determine the role of NRF2 activation in MP's modulation of antioxidant activity, ROS levels were also measured after treatment with ML385, an NRF2 inhibitor. The experimental groups were Control, ML385, TNF‐α, TNF‐α + ML385, MP + TNF‐α, and MP + TNF‐α + ML385. The concentrations of ML385, MP, and TNF‐α were 10 µM, 100 µg/mL, and 40 ng/mL, respectively. After 6 h of treatment, intracellular ROS levels were measured using DCFH‐DA; refer to Section 4.12 for experimental procedures. Western blotting was used to assess NF‐κB, IκBα, and HO‐1 expression in total cellular protein extracts, as well as nuclear NF‐κB levels. Refer to Section 4.13 for experimental procedures.

### 4.16 Construction of Psoriasis‐Like Mouse Model

A psoriasis‐like mouse model was established by daily topical application of 62.5 mg of 5% imiquimod (IMQ) cream to the shaved dorsal skin from Day 1 to Day 7. Baseline PASI was 0 on Day 1. Initial psoriatic lesions (skin thickening, erythema, and desquamation) appeared on Day 2, at which point drug administration was initiated while IMQ application continued, constituting an early‐intervention model.

### 4.17 Psoriasis‐Like Lesion Scoring and Treatment

A mouse model of psoriasis was established using 5% IMQ cream, as described in *4.16*. Mice were allocated to groups to ensure comparable baseline body weight and PASI scores across groups. Briefly, the BALB/c mice (8 weeks) were randomly classified into eight groups (*n* = 6): control group (normal mice), Model group (IMQ‐induced mice), TAP group (Tapinarof cream‐treated mice), MP‐cream group, MN group (blank MNs‐treated mice), Pd‐MN group (the residue of the same weight MP after acid dissolution), Mg‐MN group, MP‐MN group. The Mg content of MP‐cream, Mg‐MN, and MP‐MN was 3 mg. The whole experimental period was 7 days. Except for the control group, all experimental groups were topically administered with 62.5 mg IMQ cream on the dorsal skin from day 1 to day 6. Psoriatic symptoms developed in the mice on Day 2. Therefore, administration was planned from day 2 onward. For preparation of MP‐Cream, MP powders were uniformly dispersed in a cream base at a concentration of 3 mg MP per 50 mg cream. A commercially available 1% Tapinarof cream (Xinbike, China) was used. Each mouse received either 50 mg of 1% Tapinarof cream or 50 mg of MP cream applied to the dorsal skin every other day. For MN treatment groups, MNs were applied to the dorsal skin of mice once every two days. To eliminate potential interactive effects between IMQ and treatments, a 6 h interval was maintained between IMQ cream application and MN treatment. Body weight was recorded daily throughout the experimental period. The severity of psoriasis lesions was evaluated every day using the PASI score, involving in erythema, desquamation, and thickness. Psoriatic lesion severity was scored daily from 0 to 4. Both PASI scoring and body weight were performed in a blinded manner. Meanwhile, photographs of the psoriasis lesion were taken every day. Predefined exclusion criteria were weight loss > 20%, severe ulceration/infection, or death. No mice were excluded.

At the endpoint, after the mice were sacrificed, psoriasis lesion skin was collected and immersed in 4% paraformaldehyde for histopathological, immunohistochemical, and immunofluorescence analyses. Besides, fresh psoriatic skin tissue was immediately snap‐frozen in liquid nitrogen and prepared for further eukaryotic transcriptome RNA sequence analysis and ROS staining. Furthermore, mouse spleen dissociation samples were collected to analyze the expression levels of inflammatory cytokines according to ELISA kits protocols.

### 4.18 Immunohistochemistry and Immunofluorescence Analysis

After being immersed in 4% paraformaldehyde, skin tissues were harvested, fixed, embedded, and sectioned. For skin thickness evaluation, the sections were stained with hematoxylin and eosin (H&E). The expression of IL‐17A, IL‐23, IL‐22, IL‐6, and TNF‐α was assessed by immunohistochemical staining. In addition, the expression levels of NRF2, HO‐1, phosphorylated NF‐κB, and Ki67 in psoriatic skin lesions were analyzed by immunofluorescence staining.

### 4.19 Flow Cytometry of Spleen

To assess the immune cell subsets, after the end of in vivo pharmacodynamics evaluation, 100 mg of spleen tissue from mice was weighed and ground into a single‐cell suspension via an automated tissue dissociator (Shanghai Jingxin Industrial Development Co., Ltd, China). Following the elimination of erythrocytes by erythrocyte lysis buffer, the digestion was stopped with DPBS containing 2% FBS, and a 70 µm filter was utilized to receive the single‐cell suspension of spleen. Subsequently, single‐cell suspension was washed with precooled DPBS and counted to obtain a cell density of 1 × 10^7^ cells/mL. For intracellular IL‐17A staining, single‐cell suspensions were stimulated with a cell activation cocktail (with Brefeldin A, Cat. No. 423303) for 6 h at 37 °C before surface and intracellular staining. Cell pellets from 100 µL of single cell suspension were dealt with anti‐mouse CD16/32 antibody for 30 min to block Fcγ3 peptide. After washing, cells were stained with FVD455UV, BV510 anti‐mouse CD45, APC/Fire ^TM^ 810 anti‐mouse F4/80, PE/Fire ^TM^ 640 anti‐mouse Ly‐6G, Spark Blue ^TM^ 574 anti‐mouse CD3, BV570 anti‐mouse CD4, and PE/Fire ^TM^ 744 anti‐mouse CD11b antibodies diluted in DPBS buffer for 30 min on ice in the dark. The cell suspension was centrifuged at 2000 rpm for 5 min, and cells were incubated with the fixation/permeabilization concentrate for 25 min on ice. Then, cells were mixed with 1 × permeabilization buffer for 30 min to open the cell membrane. Following incubation with APC anti‐mouse IL‐17A, PE anti‐mouse FoxP3, Spark red 718 anti‐mouse CD86, and BV785 anti‐mouse CD206 antibodies for 30 min, the stained cells were acquired and analyzed by a full spectral flow cytometer (Aurora, Cytek Biosciences, USA). Single‐stain controls were used for compensation. All samples were processed with identical gating strategies.

### 4.20 Eukaryotic Transcriptome RNA Sequencing Analysis Method

Total RNA was extracted using TRIzol reagent. To ensure RNA quality, RNA purity and concentration were measured using a NanoDrop 2000, with OD260/280 between 1.8 and 2.2 for all RNA samples. RNA integrity was assessed by determining the RQN value with an Agilent 5300 Bioanalyzer. The RNA samples were free from pigment contamination and showed no obvious contamination by proteins, polysaccharides, or other impurities. All samples had an RQN ≥ 7.0, and the total amount met the requirement for one standard library preparation, making them suitable for subsequent experiments. Libraries were sequenced on an Illumina NovaSeq platform. Each sample generated at least 5.53 Gb of clean data, corresponding to a sequencing depth sufficient for transcriptome analysis. Clean reads were aligned to the mouse reference genome (GRCm38/mm10) using HISAT2 with default parameters. The alignment rates ranged from 96.9% to 97.43% across all samples. DEGs were identified using DESeq2. Genes with |log2 fold change| > 1 and adjusted P adjust < 0.05 were considered significantly differentially expressed. For FDR correction, P values were adjusted for multiple testing using the Benjamini–Hochberg method. GSEA was performed using the GSEA software with KEGG gene sets from Molecular Signatures Database (MSigDB). The Signal2Noise ranking metric was applied, and gene sets with sizes between 15 and 500 were included. Pathways with adjusted P adjust< 0.25 were considered significantly enriched.

### 4.21 Establishment of A Mouse Model for Psoriasis Recurrence

A mouse model of psoriasis‐like relapse was established by two rounds of IMQ challenge. On days 1–7, 62.5 mg of 5% IMQ cream was applied to the shaved dorsal skin of each mouse. Tapinarof cream or MP‐MN was applied to the dorsal skin every other day during this initial treatment period. From day 8, IMQ application was discontinued for a 7‐day recovery period. On days 15–21, the dorsal skin was rechallenged with a lower dose of 5% IMQ cream (20.8 mg). Body weight and PASI scores were monitored every two days, and skin lesions were photographed during the whole experiment. On day 22, the mice were euthanized, and their spleens were collected, weighed, and photographed.

### 4.22 Biosafety Analysis

To evaluate biosafety of MNs and creams, histopathological analysis, blood routine indexes, and serum biochemical indexes in healthy mice administered three times were measured. The harvested major organs were fixed, dehydrated, and embedded in paraffin. Organ tissue was cut into sections, accompanied by H&E staining and scanning via a high‐throughput tissue slice scanner (VS200, Olympus, Japan). Furthermore, mouse serum from each group was obtained and tested for the values of aspartate aminotransferase (AST), alanine transaminase (ALT), Mg content, blood urea nitrogen (BUN), and creatinine (CREA). Whole blood samples were tested for blood routine indexes such as white blood cell (WBC), lymphocyte (Lymph), monocyte, neutrophil, red blood cell (RBC), red cell distribution width (RDW), mean corpuscular volume (MCV), and platelet (PLT).

After pressing MP‐MN to the mouse skin for 15 s, the MP‐MN patch was removed, and the skin condition was recorded using an electronic device at various time points (0, 10, and 20 min). The procedure was repeated three times at 1 h intervals.

To evaluate the accumulation of Mg and Pd, BALB/C mice was divided into two groups: the Control group (normal mice) and the MP‐MN group. For mice in the MP‐MN group, MP‐MN was applied to the skin for 24 h and then removed. After 24 h, the mice were euthanized, and the skin and major organs (heart, liver, spleen, lungs, and kidneys) were collected, weighed, acid‐digested, and centrifuged to collect the supernatant. The Mg and Pd contents in the supernatant were determined by ICP (iCAP 7600, Thermo, USA) and ICP‐MS (iCAP Q, Thermo, USA), respectively.

### 4.23 Statistical Analysis

All experiments were performed by at least three independent samples. Data was presented as mean ± SD and was analyzed using GraphPad Prism 9.0 software. Statistical comparisons were performed by two‐tailed t‐test or one‐way analysis of variance (ANOVA). PASI score and body weight were analyzed by using a repeated‐measures two‐way ANOVA with an interaction term, followed by Tukey's multiple comparison test to correct for multiple comparisons. Sphericity was assessed using Mauchly's test; when sphericity was violated, the Geisser‐Greenhouse correction was applied. Image J software was used for quantitative analysis of immunohistochemistry, immunofluorescence, and western blot. Statistical significance was indicated as ^*^ *p* < 0.05, ^**^ *p* < 0.01, and ^***^ *p* < 0.001.

## Author Contributions

**Xinyue Shao**: Writing – original draft, Investigation, Visualization, Validation, Conceptualization, Methodology, Formal analysis, Data curation. **Zongyan He**: Validation, Methodology, Formal analysis. **Ning Yang**: Visualization, Validation, Methodology. **Lixuan Yin**: Validation, Methodology. **Xujie Sun**: Validation, Methodology. **Jingtong Qi**: Validation, Methodology. **Xiaolong Feng**: Validation, Methodology. **Yihui Zhang**: Validation, Methodology. **Yiran Liu**: Validation, Methodology. **Zhengwei Huang**: Validation, Methodology, Funding acquisition. **Tianqun Lang**: Writing – review & editing, Supervision, Investigation, Funding acquisition, Project administration, Data curation, Conceptualization.

## Conflicts of Interest

The authors declare no conflicts of interest.

## Supporting information




**Supporting File**: advs78709‐sup‐0001‐SuppMat.pdf.

## Data Availability

The data that support the findings of this study are available from the corresponding author upon reasonable request.
