# Supplementary material for: Localized Hydrogen Release Driven by Mg–Pd Microgalvanic Cells from 3D‐Printed Microneedles Reverses Redox Homeostasis for Psoriasis Treatment
Source: Adv Sci (Weinh). 2026 Nov 9:e78709. Online ahead of print. doi: 10.1002/advs.78709 (PMC13748887; doi:10.1002/advs.78709)
Supplement: Supplementary file 1 [file ADVS-9999-e78709-s001.pdf]

Supporting Information

Localized Hydrogen Release Driven by Mg–Pd Microgalvanic Cells from 3D-Printed Microneedles Reverses Redox Homeostasis for Psoriasis Treatment

*Xinyue Shao [a, b], Zongyan He [a, b], Ning Yang [b], Lixuan Yin [a, b], Xujie Sun [a, b], Jingtong Qi [b], Xiaolong Feng [b, c], Yihui Zhang [b], Yiran Liu [b], Zhengwei Huang [d], Tianqun Lang [a, b, c] **

[a] *School of Life Science and Technology, ShanghaiTech University, Shanghai 201210, China*

[b] *Lingang Laboratory, Shanghai 200031, China*

[c] *School of Pharmacy, East China Normal University, Shanghai 200062, China*

[d] *State Key Laboratory of Bioactive Molecules and Druggability Assessment, Guangdong Basic Research Center of Excellence for Natural Bioactive Molecules and Discovery of Innovative Drugs, College of Pharmacy, Jinan University, Guangzhou 511443, China*

*** Corresponding author:**

Tianqun Lang, Ph.D., Prof

E-mail addresses: langtq@lglab.ac.cn (T. Lang)

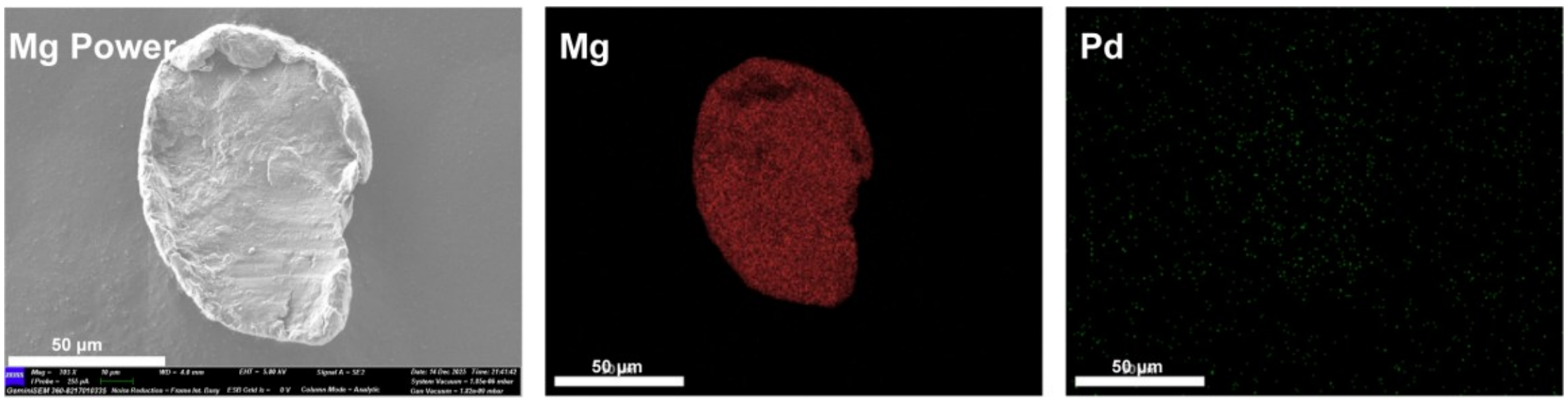

Figure S1. SEM image and EDS mapping of Mg microparticles (Scale bar: 50 μm).

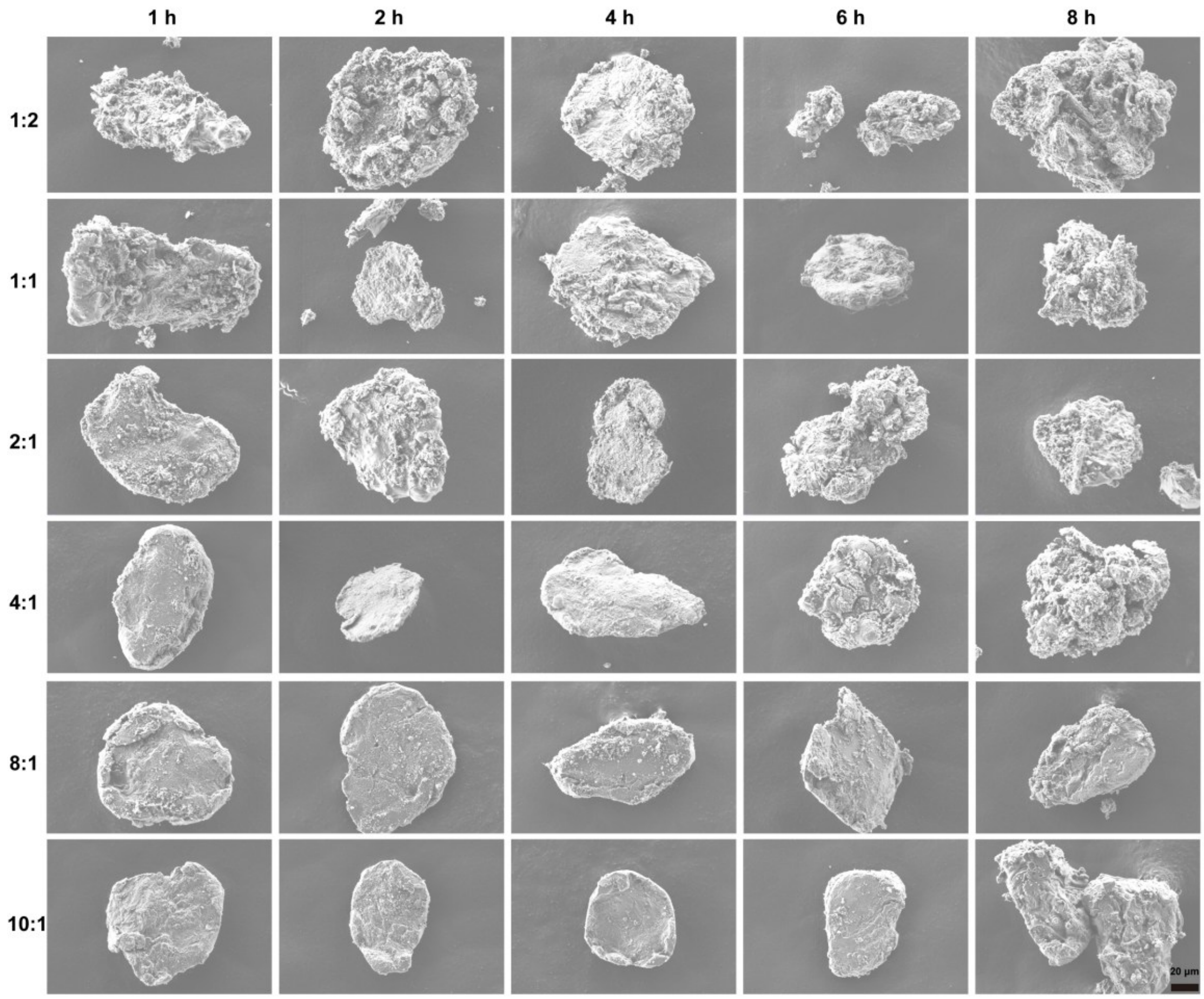

Figure S2. SEM image of MP under different ratios (Mg: Pd, w/w) and reaction times (Scale bar: 20 μm).

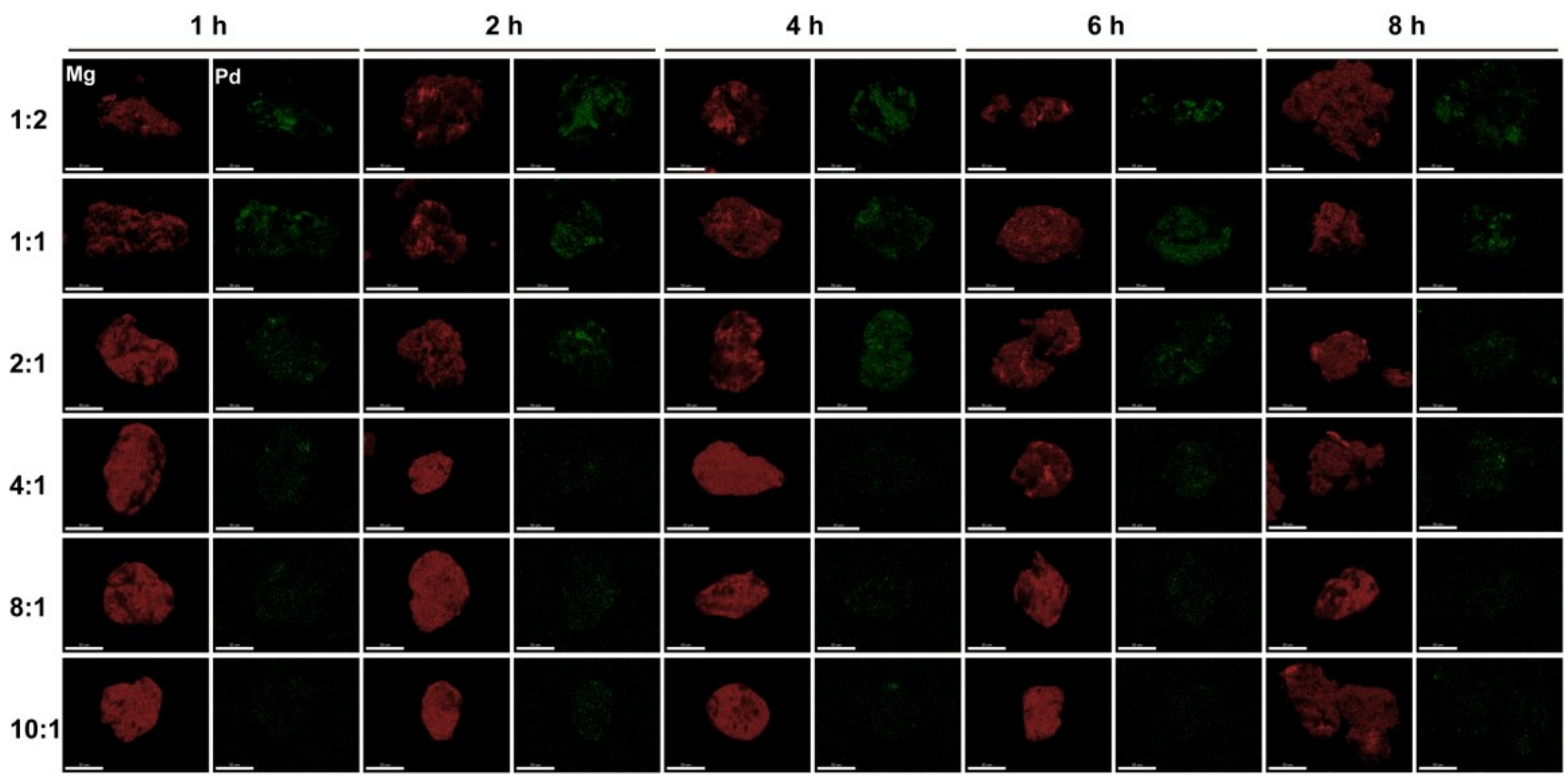

Figure S3. EDS mapping of MP under different ratios (Mg: Pd, w/w) and reaction times (Scale bar: 50 μm).

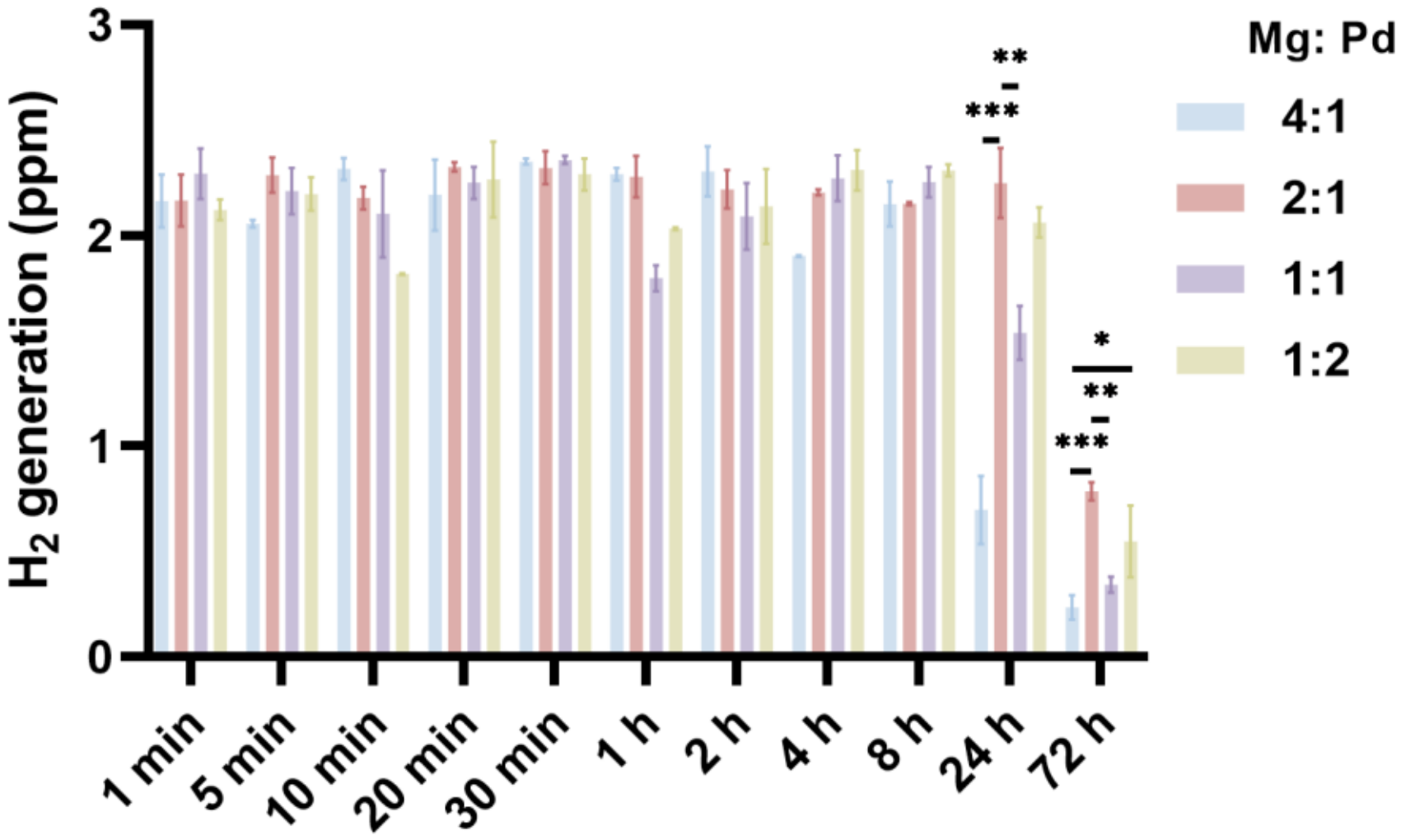

Figure S4. Time-dependent H_2 generation from MP under different ratios (Mg: Pd, w/w). Data were shown as the mean ± SD. For each group, n = 3 independent samples. * $p < 0.05$, ** $p < 0.01$, *** $p < 0.001$.

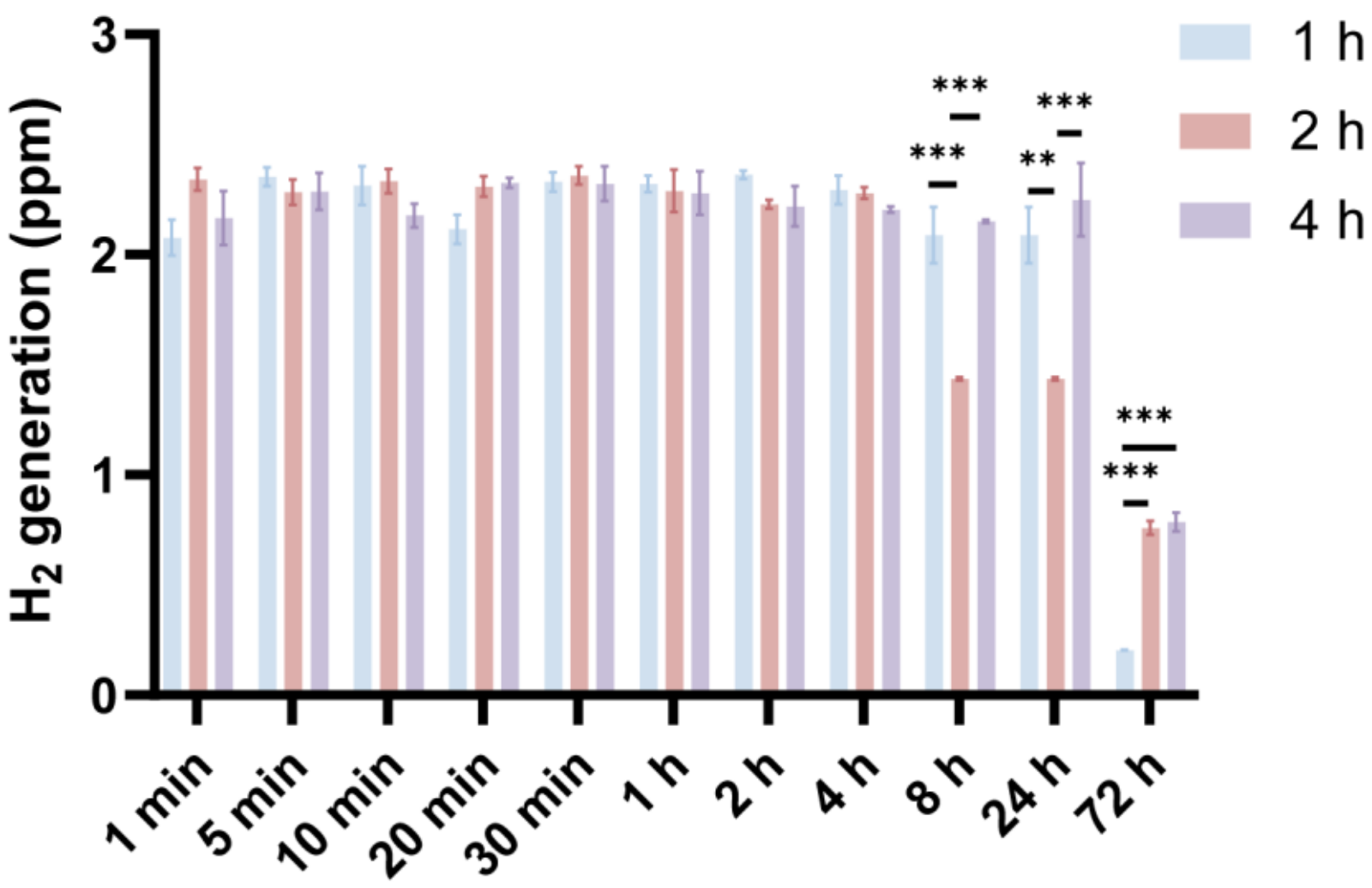

Figure S5. Time-dependent H_2 generation from MP under different reaction times. Data were shown as the mean ± SD. For each group, n = 3 independent samples. ** $p < 0.01$, *** $p < 0.001$.

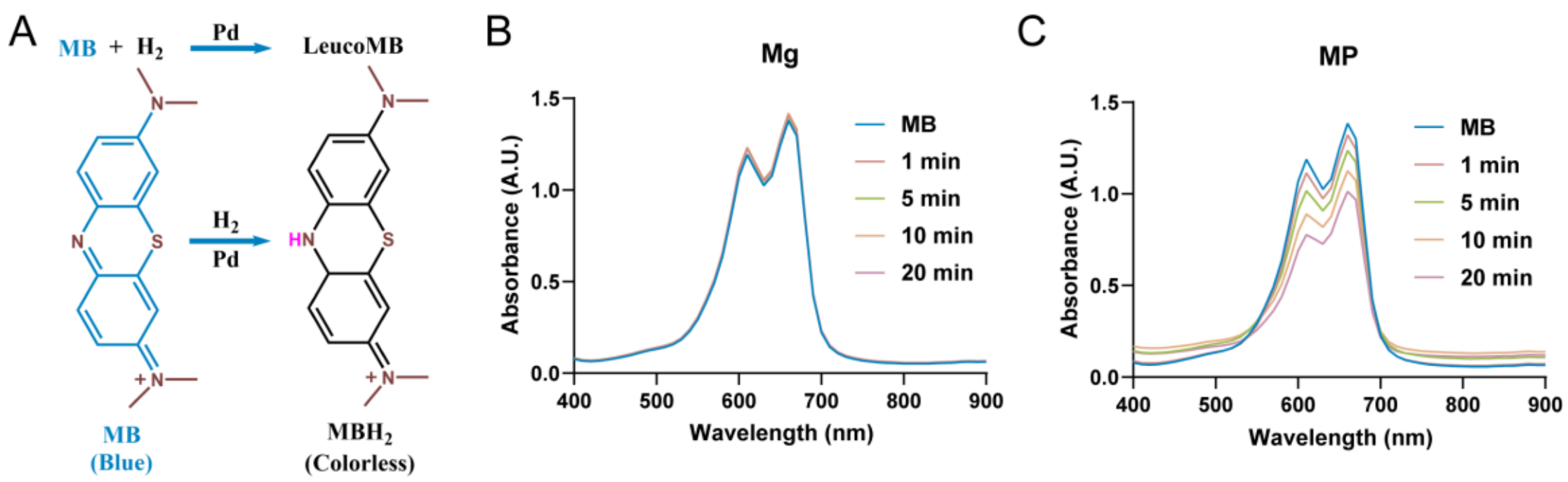

Figure S6. The absorption spectra of the MB solution with Mg or MP added. (A) Schematic diagram of H_2 production detected by the MB probe. (B) Time-dependent absorption spectra of the MB solution with Mg powders added. (C) Time-dependent absorption spectra of the MB solution with MP added.

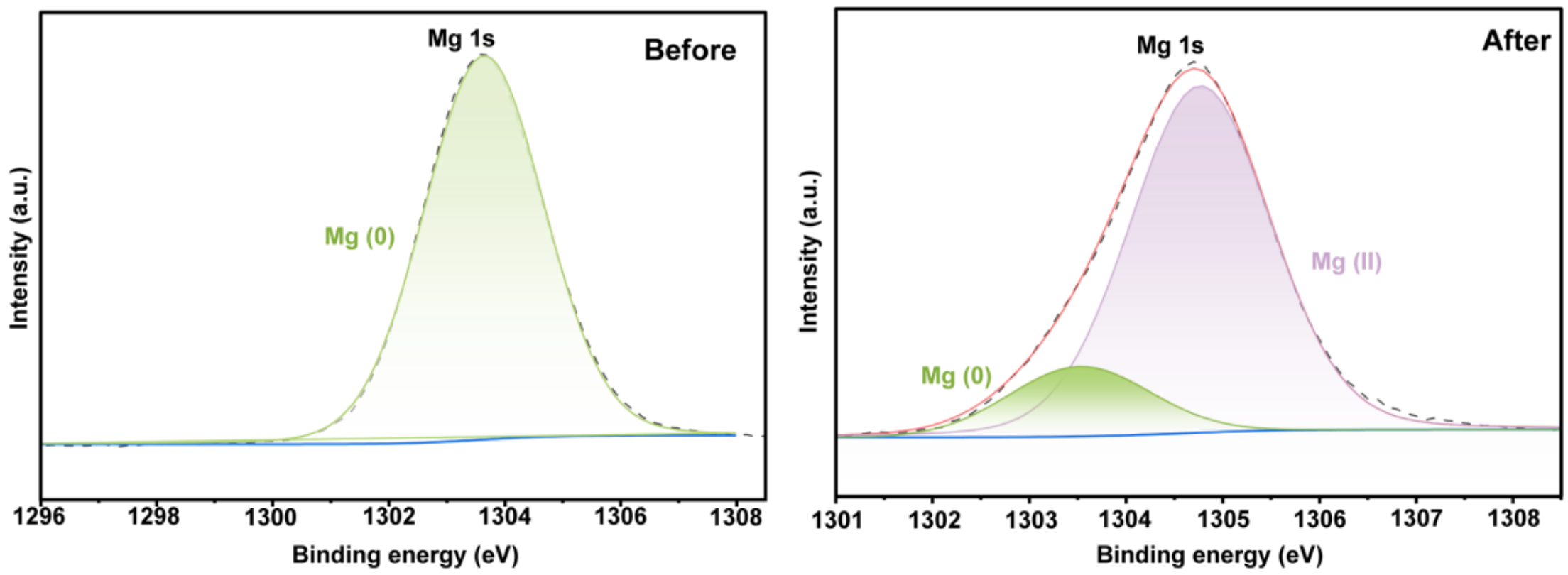

Figure S7. XPS spectra of Mg powders before and after reaction with PBS.

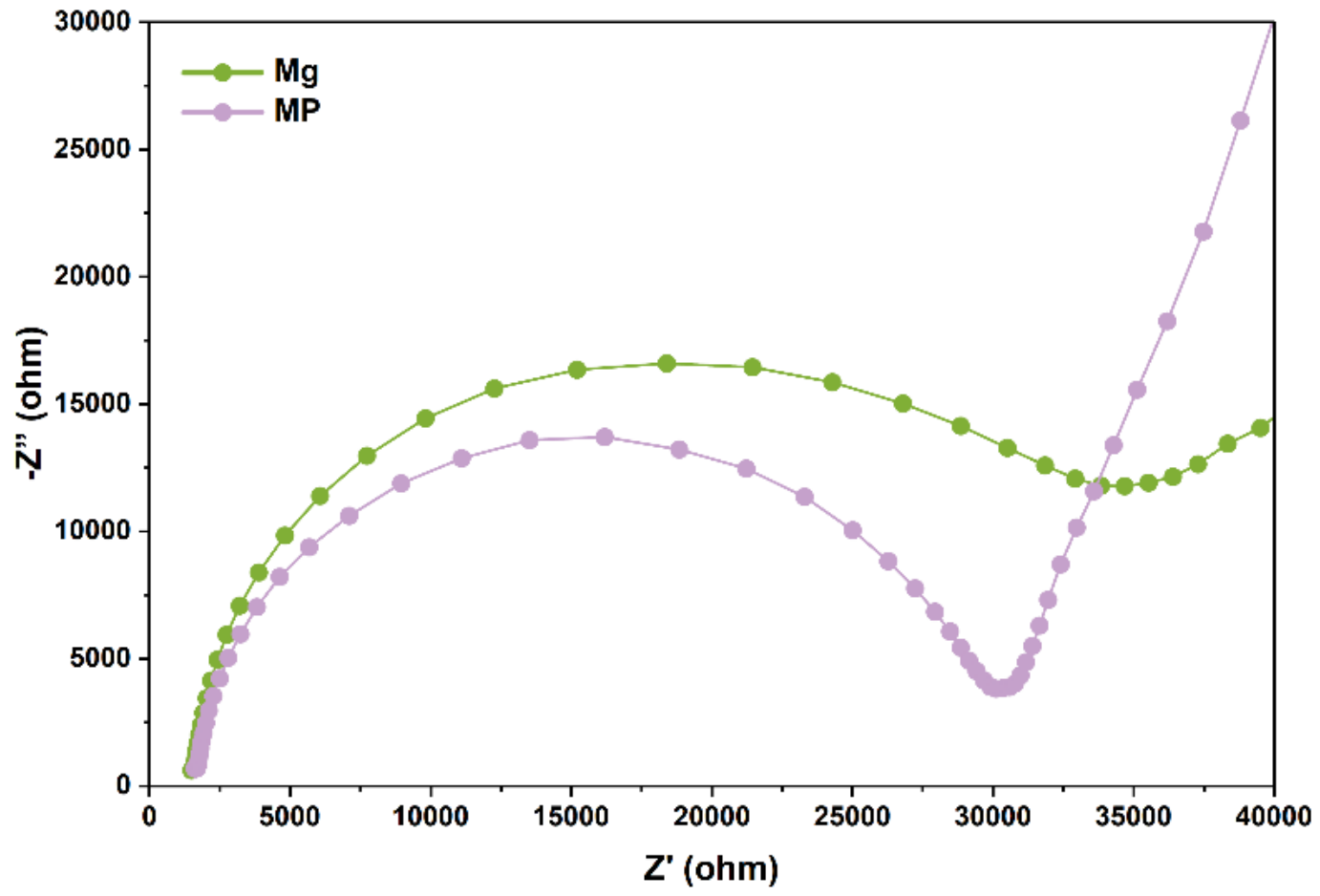

Figure S8. The electrochemical impedance spectroscopy (EIS) mapping of Mg and MP.

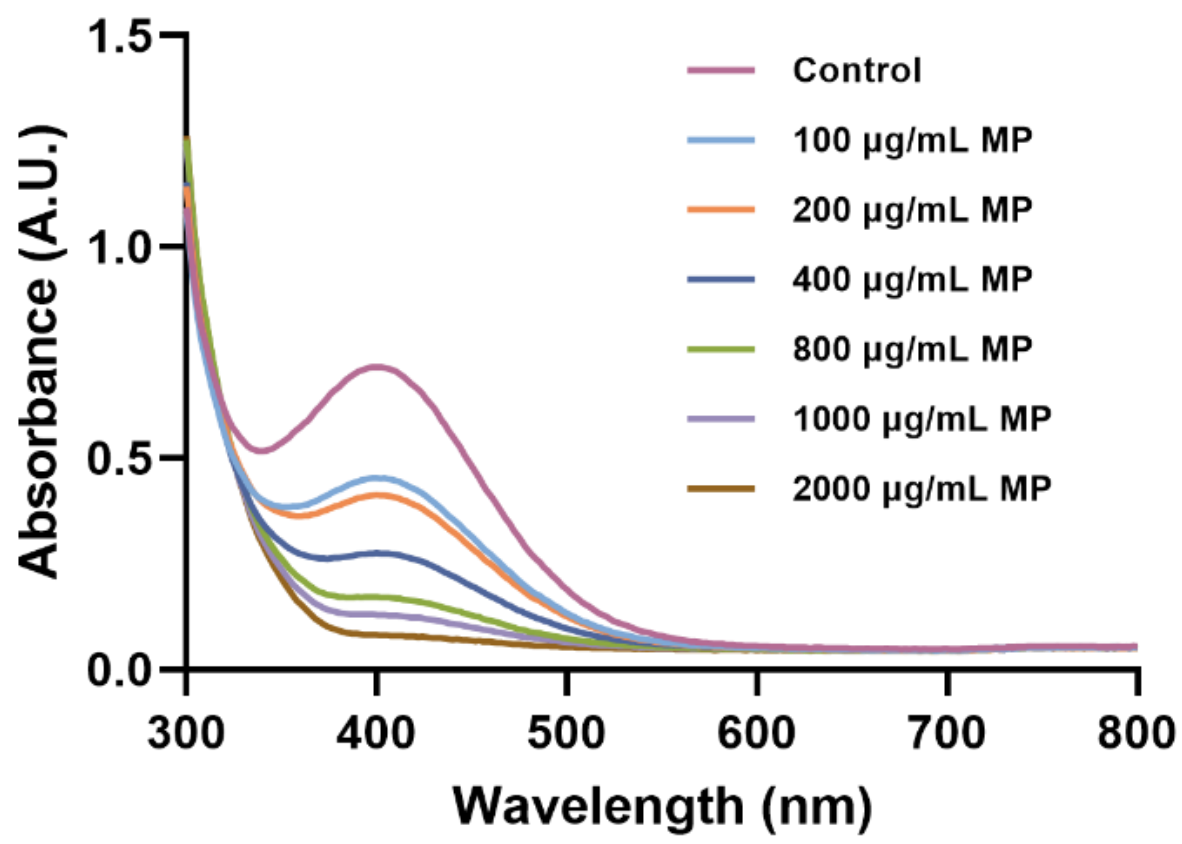

Figure S9. The scavenging assay of H_2O_2 of MP powders.

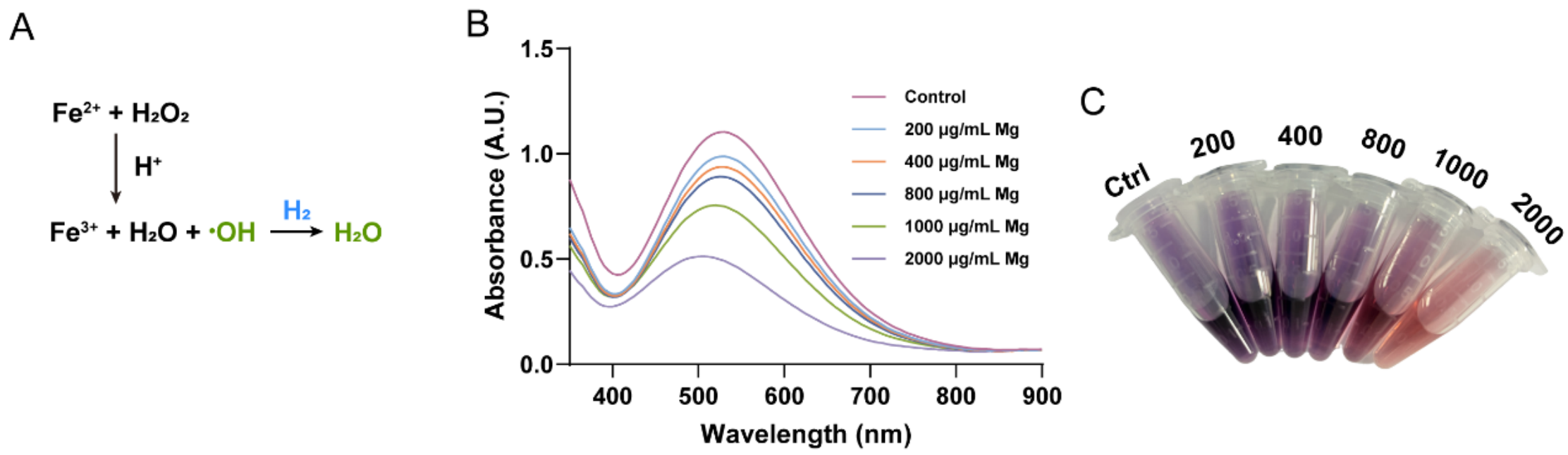

Figure S10. The scavenging assay of •OH of Mg powders. (A) Schematic diagram of H_2 scavenging •OH. (B) Absorption spectra of •OH after incubation with different concentrations of Mg powders. (C) The color changes of •OH in assay solution incubated with different concentrations of Mg powders.

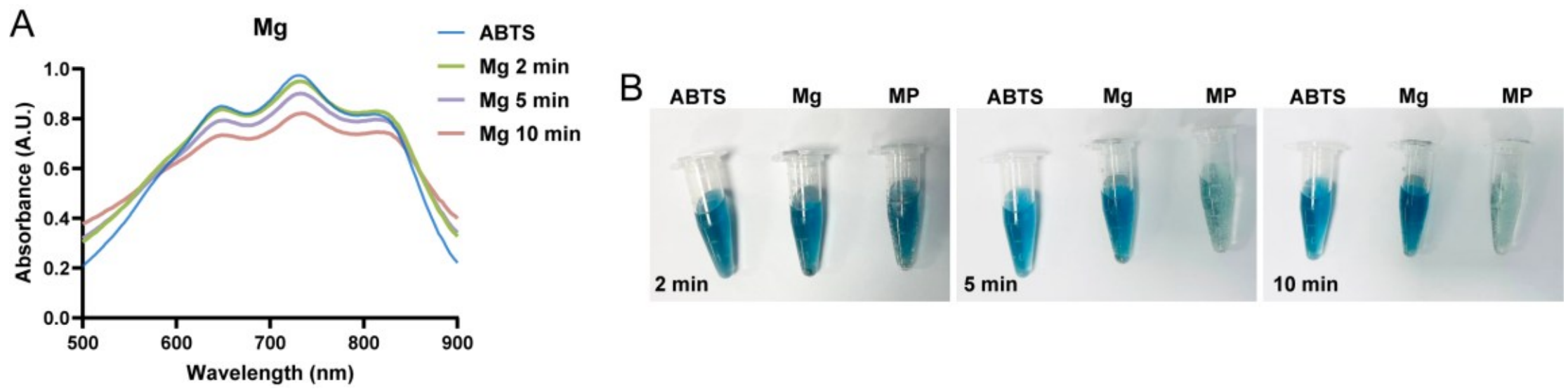

Figure S11. The scavenging assay of $ABTS^{\bullet+}$. (A) Time-dependent absorption spectra of $ABTS^{\bullet+}$ after incubation with Mg powders. (B) The color changes of ABTS assay solution with Mg or MP at different time points.

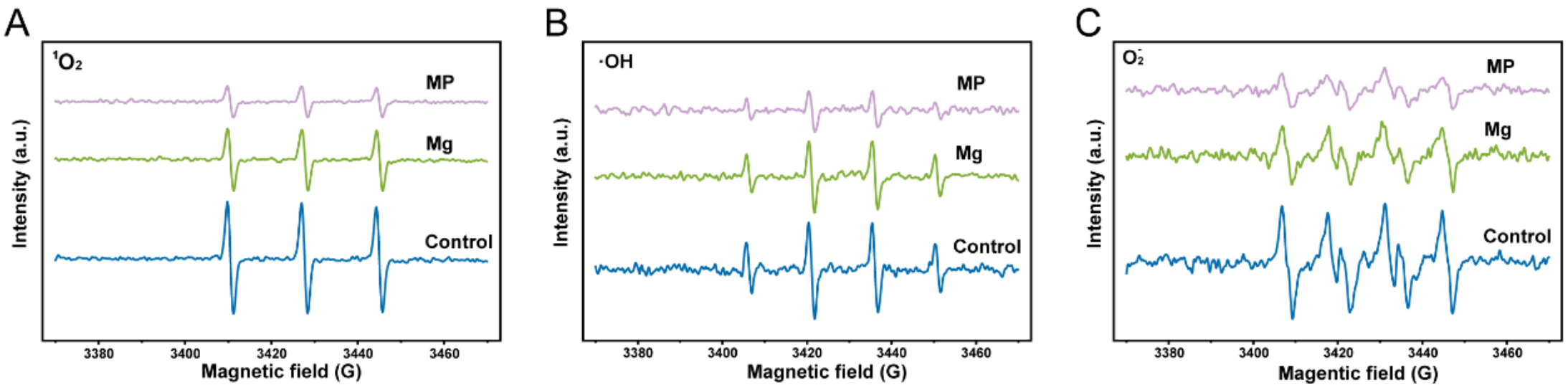

Figure S12. Electron spin resonance (ESR) spectra of (A) $^1O_2$, (B) •OH, and (C) O_2^- generated by Mg or MP powders.

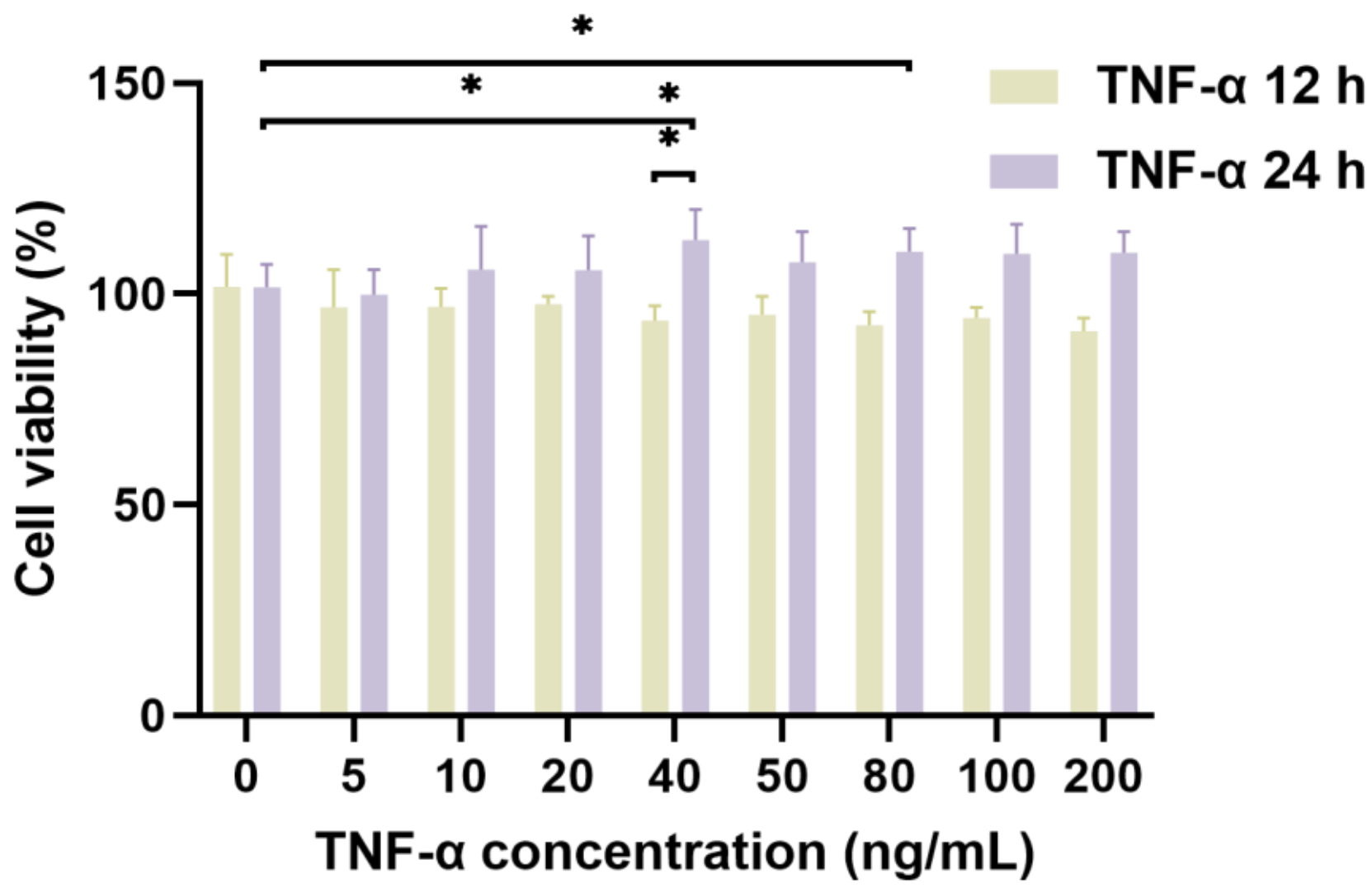

Figure S13. Cell viabilities of HaCaT cells under different TNF-α concentration treatment. Data were shown as the mean ± SD. For each group, n = 3 independent samples. * $p < 0.05$.

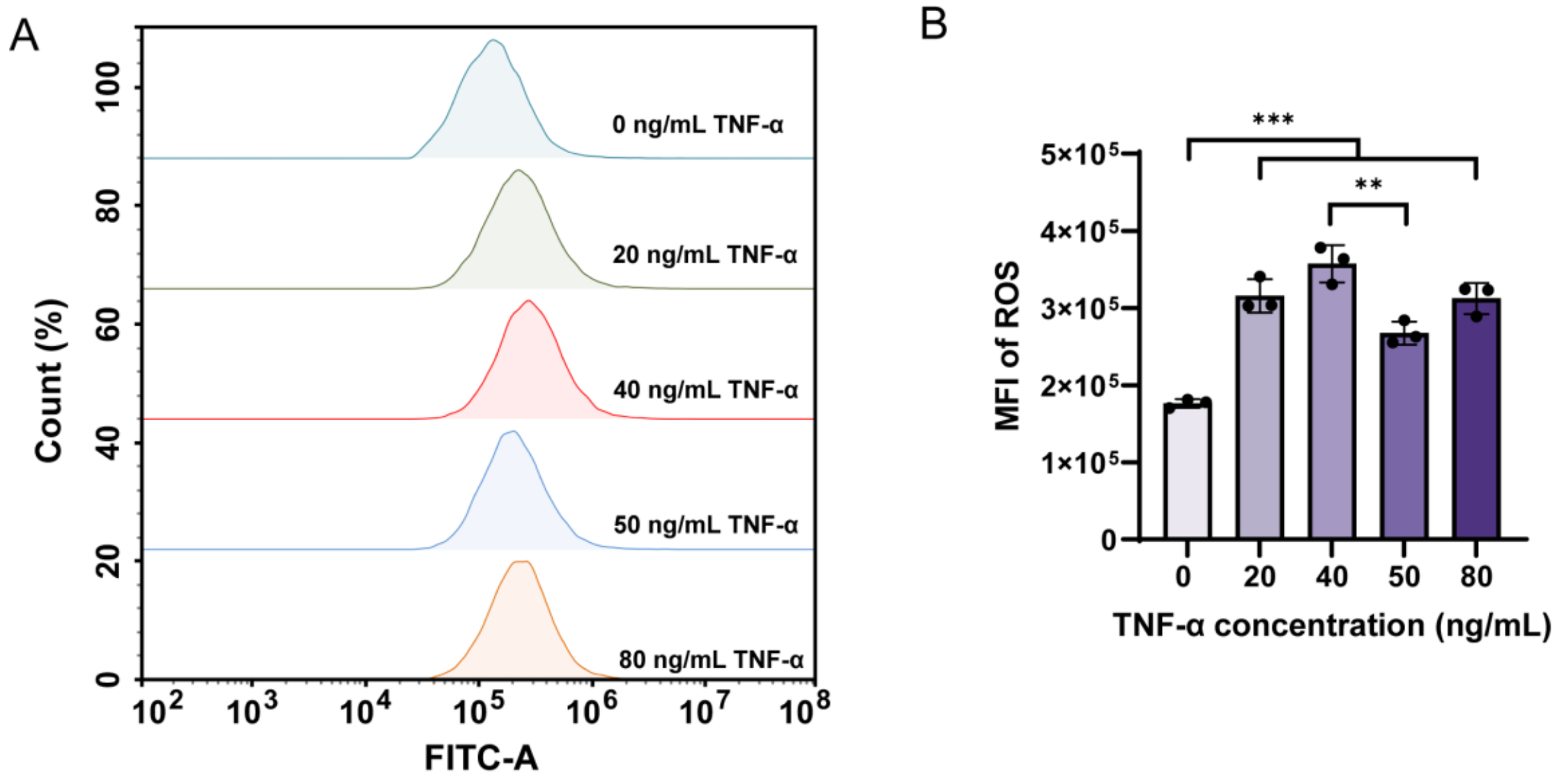

Figure S14. Flow cytometry of ROS in HaCaT cells under different TNF-α concentration treatment. (A) Flow cytometric images of ROS. (B) The quantitative analysis of ROS. Data were shown as the mean ± SD. For each group, n = 3 independent samples in (B). ** $p < 0.01$, *** $p < 0.001$.

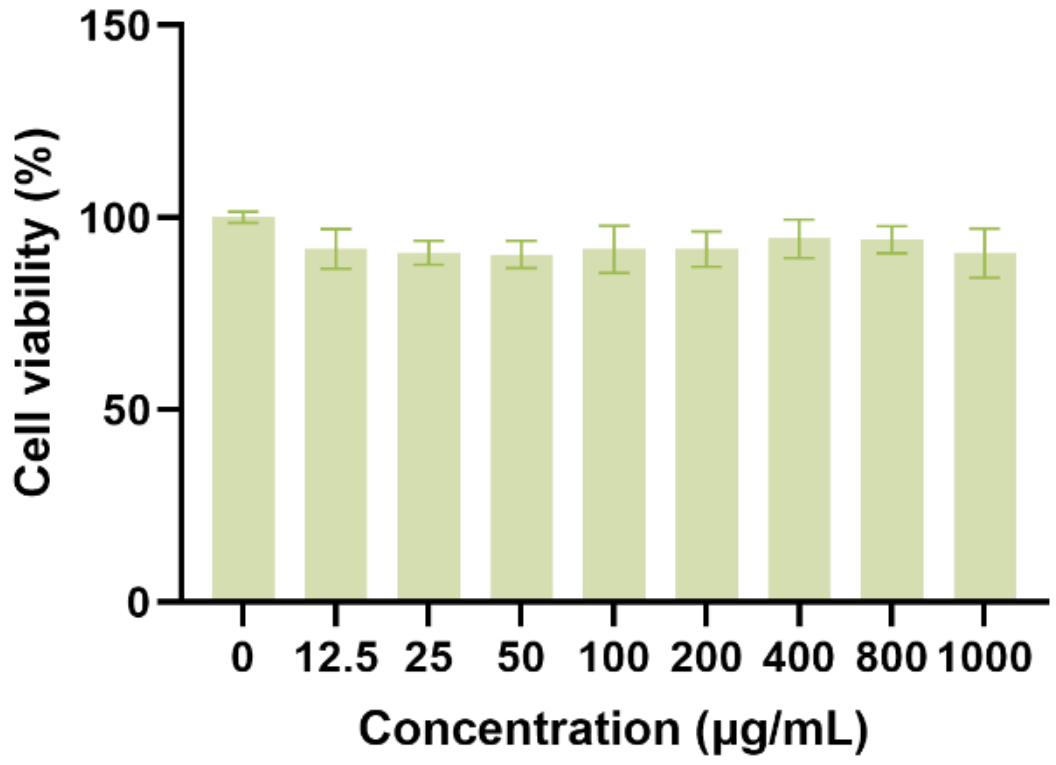

Figure S15. CCK-8 assay of HaCaT cell viability following exposure to Pd residues recovered from MP acid digestion (at concentrations equivalent to 0–1000 μg/mL Mg) for 24 h. Data were shown as the mean ± SD. For each group, n = 4 independent samples.

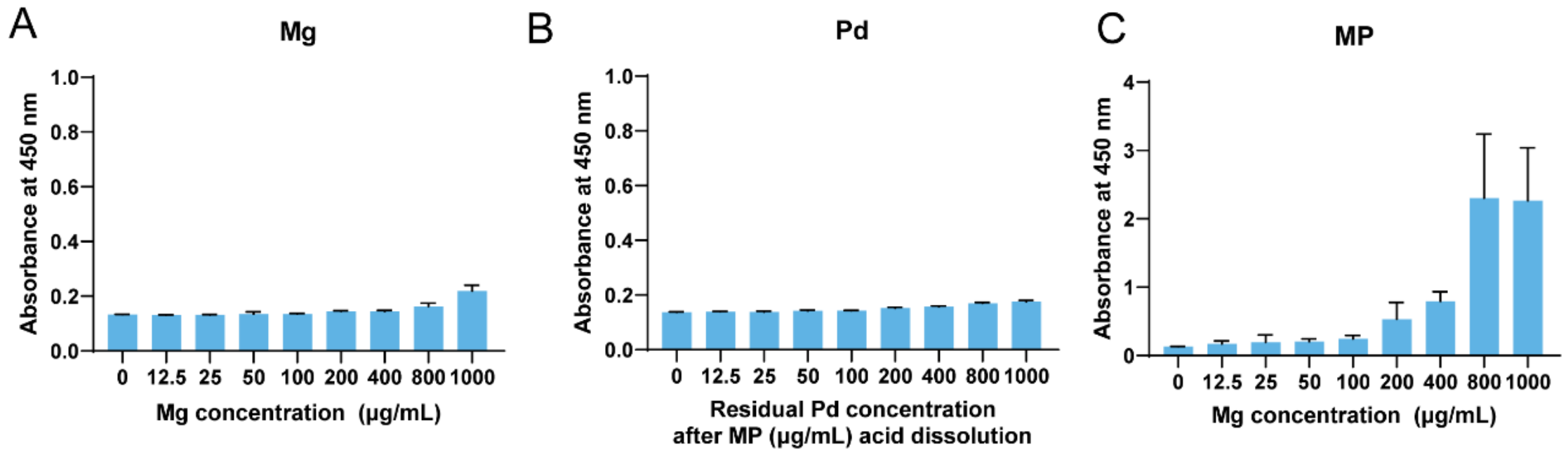

Figure S16. Cell-free control for CCK-8 interference. Absorbance at 450 nm of CCK8 with different concentration Mg, Pd, and MP. Data were shown as the mean ± SD. For each group, n = 3 independent samples.

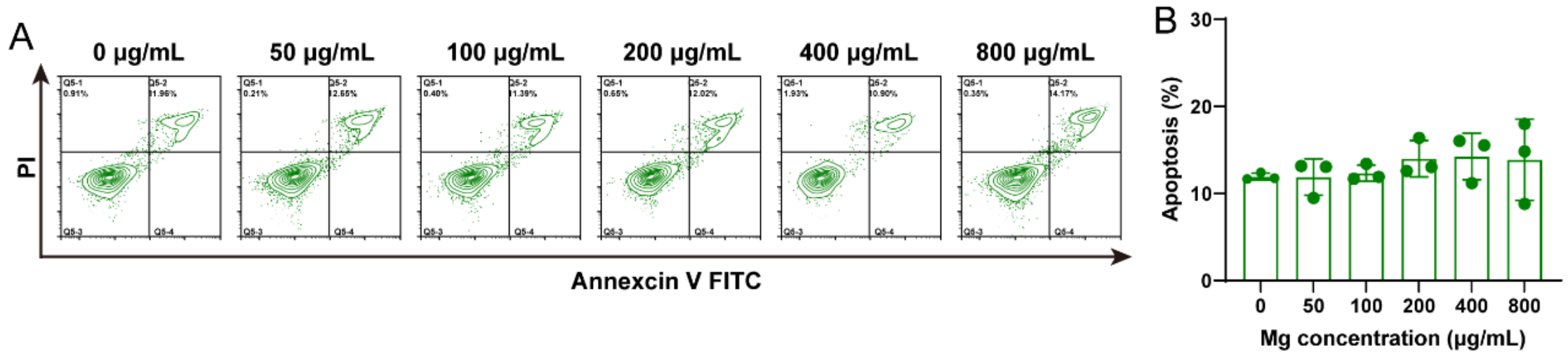

Figure S17. Apoptosis analysis of different concentration MP on TNF-α- induced HaCaT cells. Represent flow images (A) and quantitative analysis (B) of TNF-α-induced HaCaT cells under different concentration MP treatments. Data were shown as the mean ± SD. For each group, n = 3 independent samples in (B).

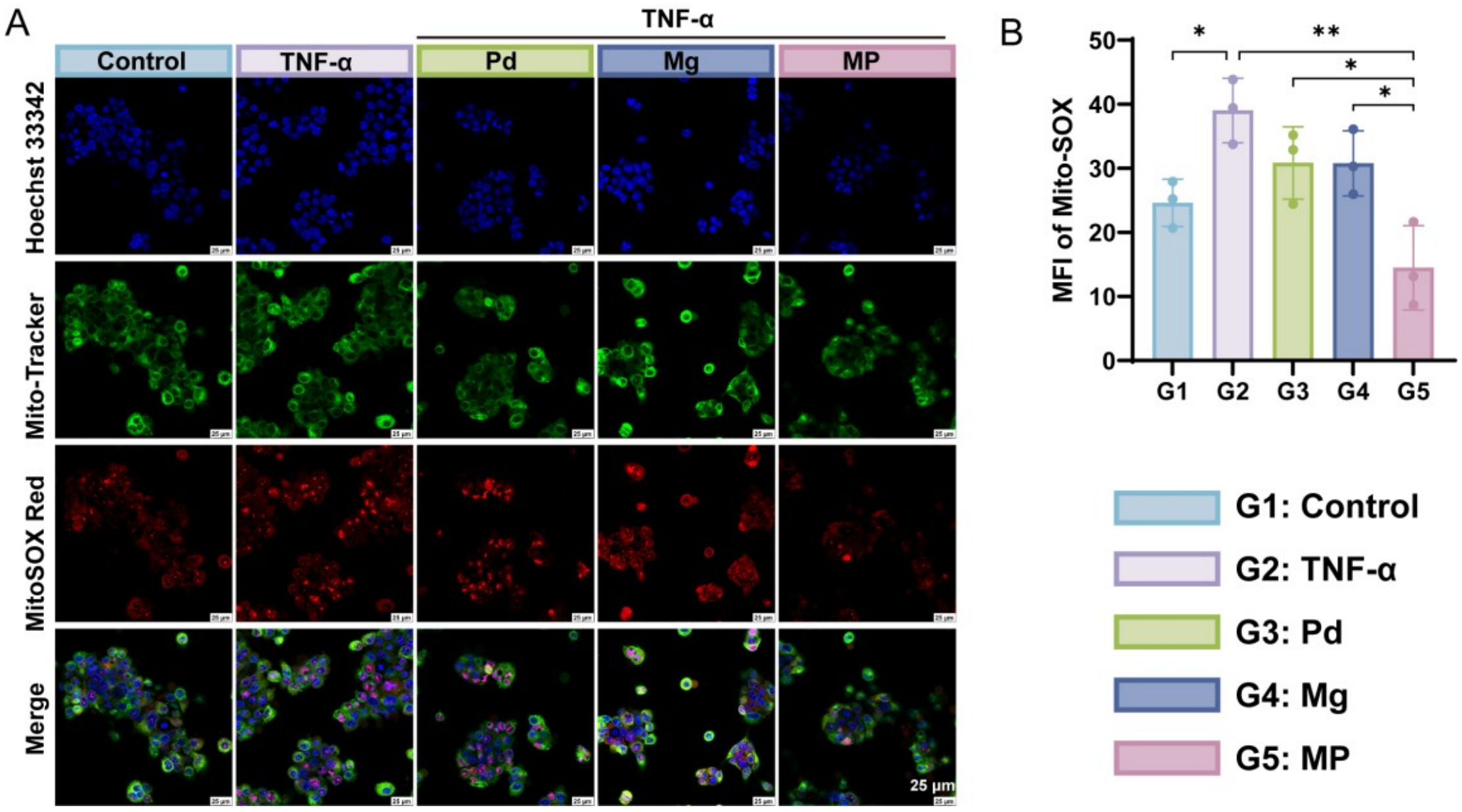

Figure S18. The O_2^- levels in the mitochondria of TNF-α induced HaCaT cells after various treatments. (A) Confocal fluorescence images of O_2^- levels. (Scale bar: 25 μm) (B) The mean fluorescence intensity (MFI) of O_2^- levels. Data were shown as the mean ± SD. For each group, n = 3 independent samples in (B). * $p < 0.05$, ** $p < 0.01$.

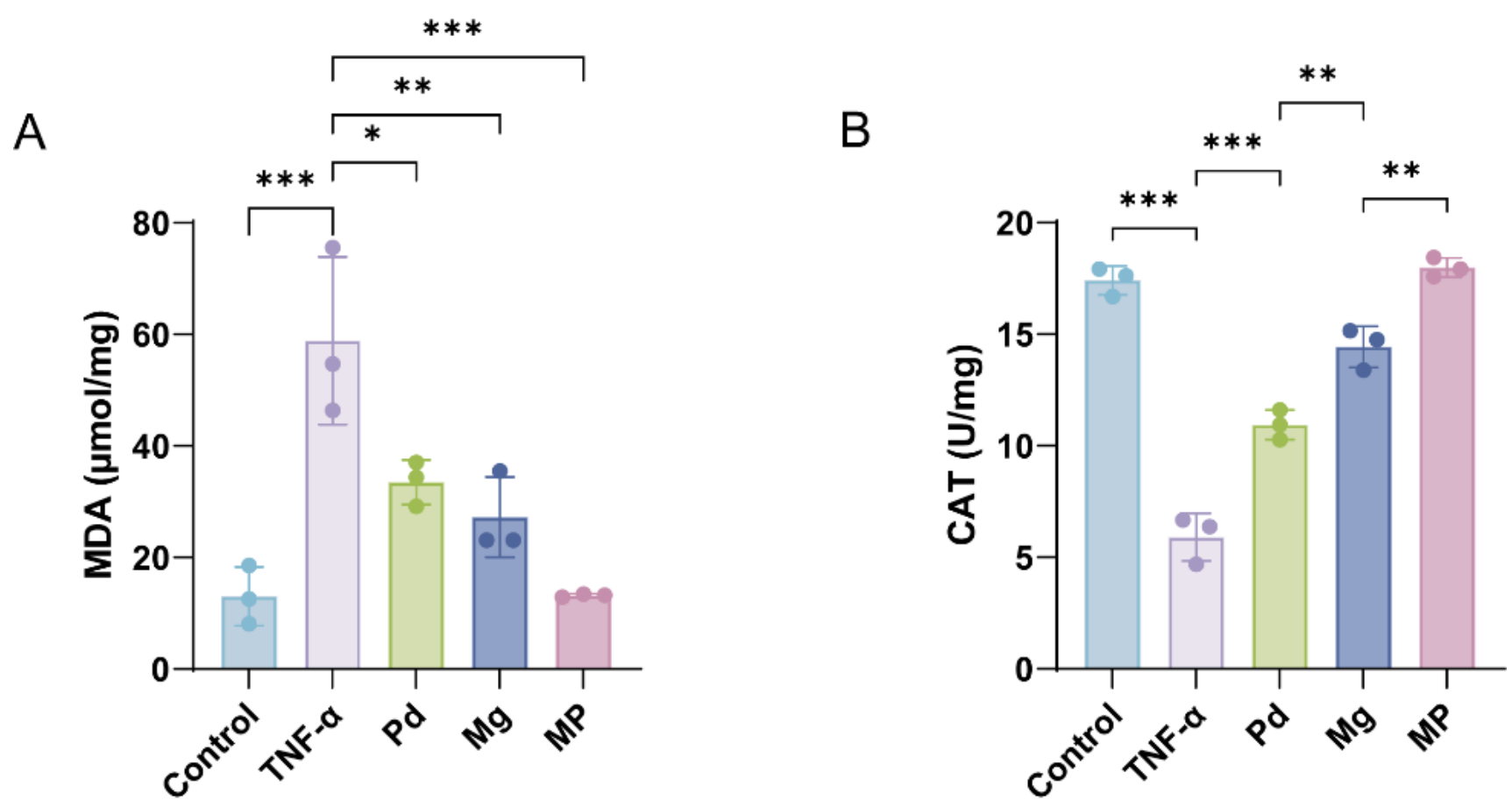

Figure S19. (A) MDA levels in TNF-α-induced HaCaT cells after different treatments. (B) CAT activity in TNF-α-induced HaCaT cells after different treatments. Data were shown as the mean ± SD. For each group, n = 3 independent samples in (A-B). * $p < 0.05$, ** $p < 0.01$, *** $p < 0.001$.

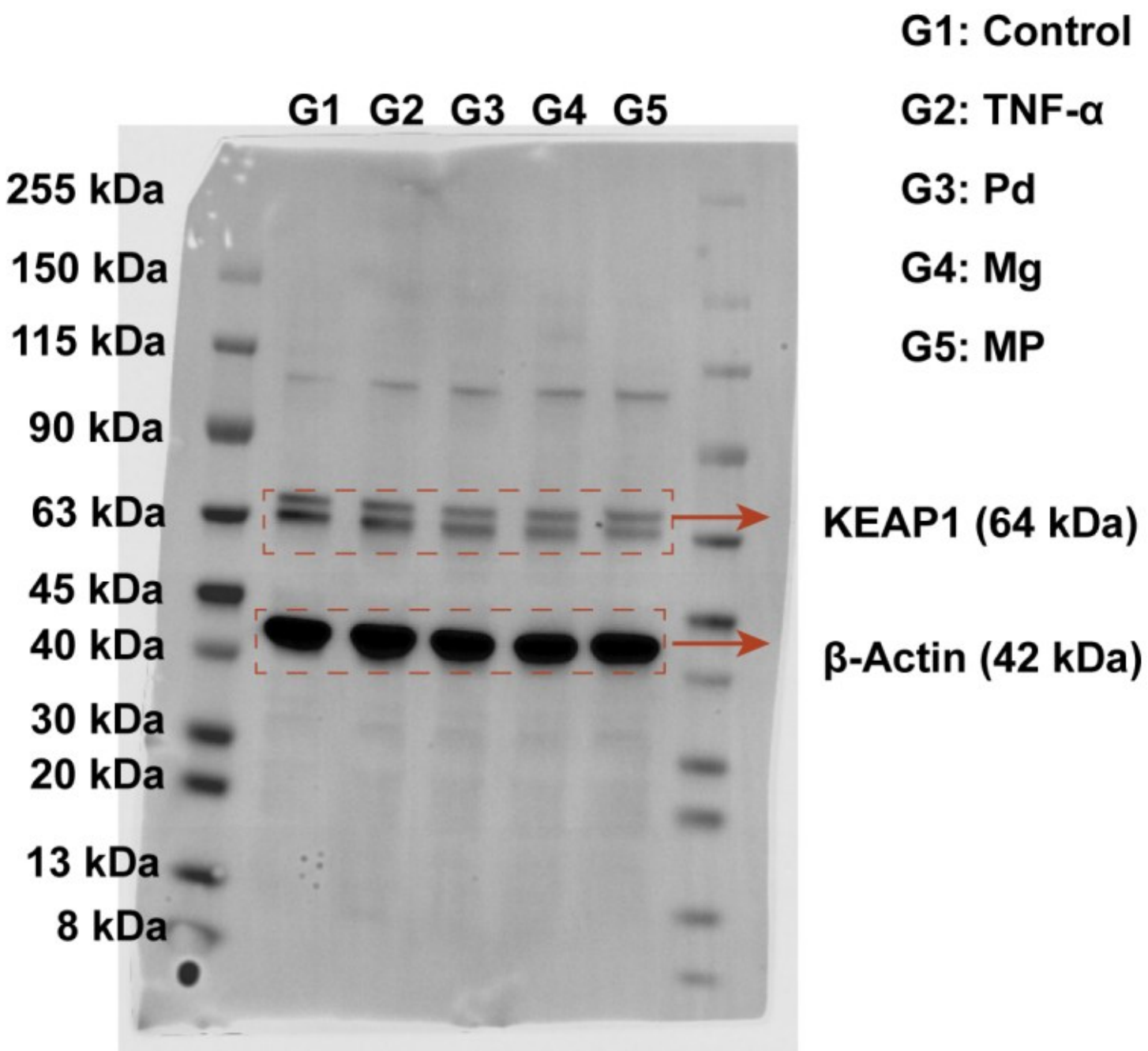

Figure S20. The complete results of western blotting of KEAP1 expression in TNF-α induced HaCaT cells after different treatments.

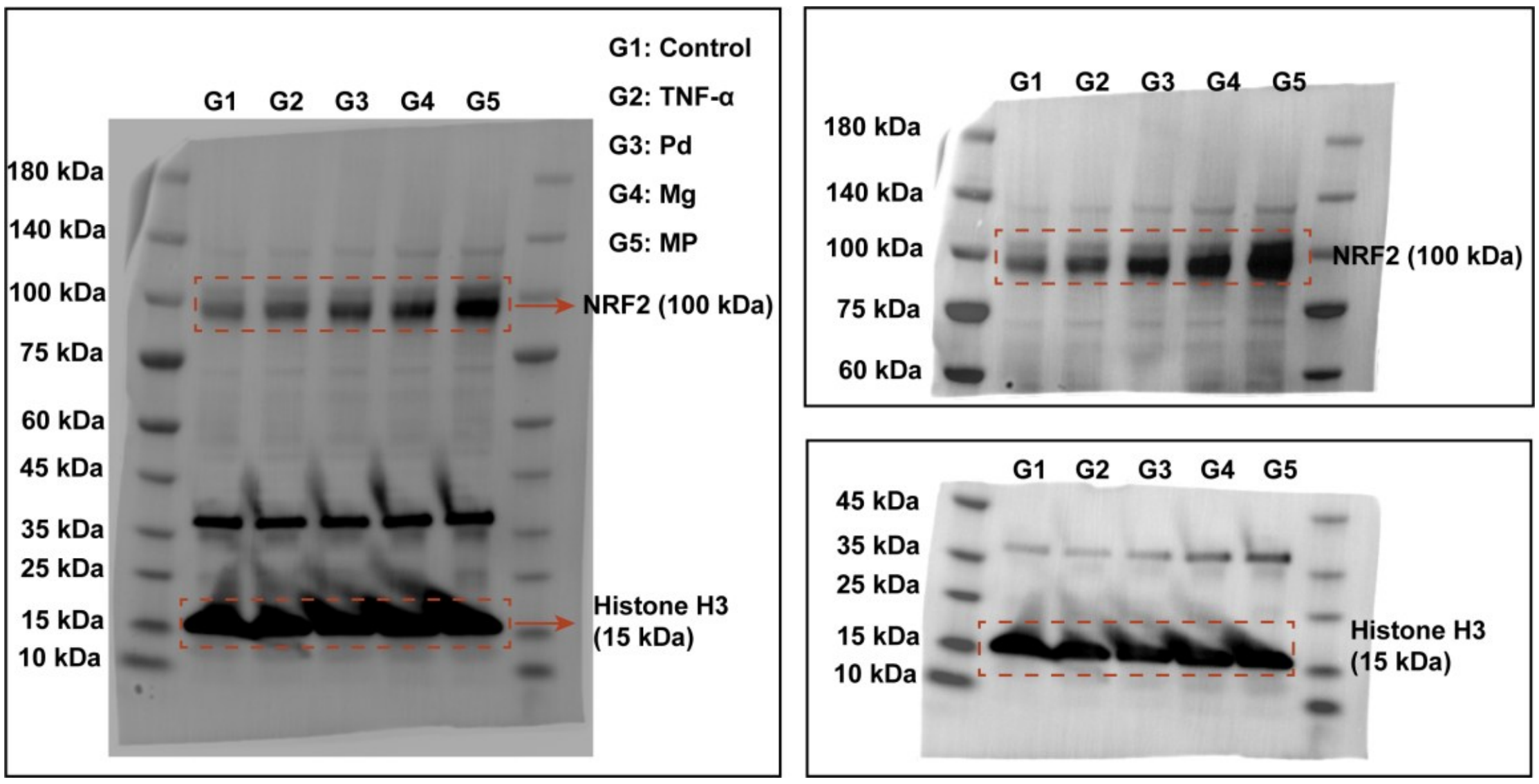

Figure S21. The complete results of western blotting of NRF2 expression in TNF-α induced HaCaT cells after different treatments.

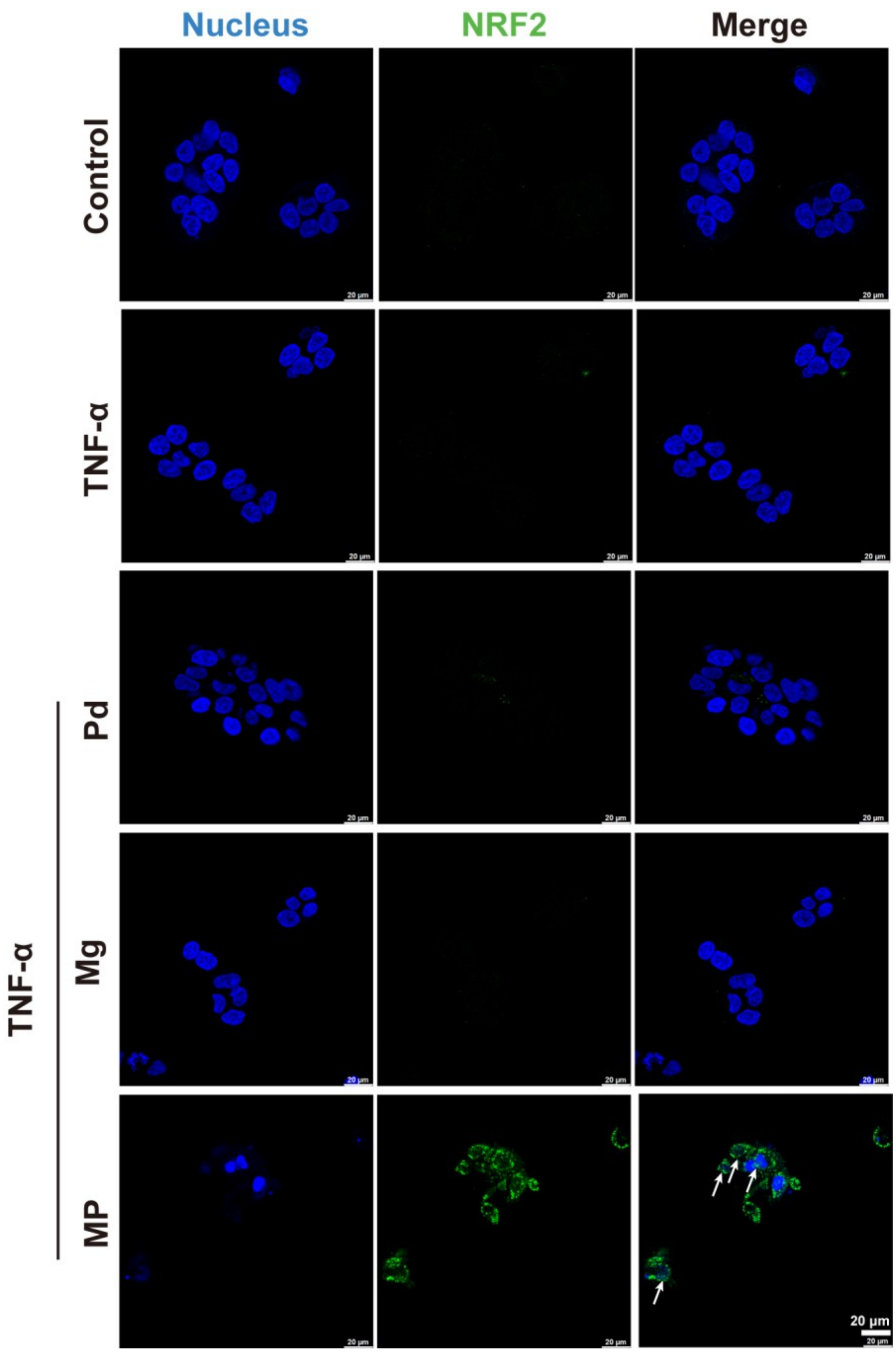

Figure S22. Confocal fluorescence images of nucleus translocation of NRF2 in TNF-α induced HaCaT cells under various treatments (Scale bar: 20 μm). The white arrow represents the cells with nuclear translocation of NRF2.

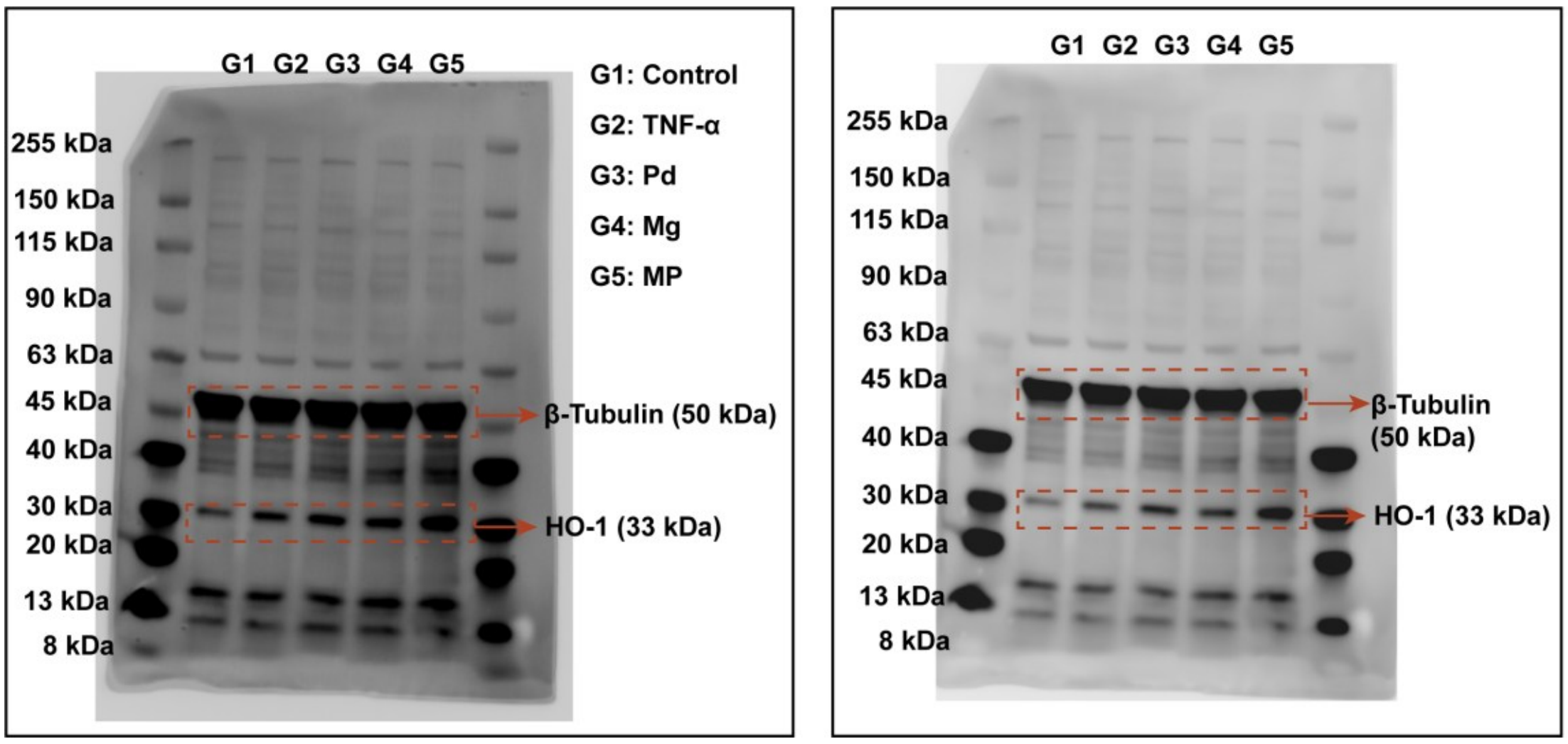

Figure S23. The complete results of western blotting of HO-1 expression in TNF-α induced HaCaT cells after different treatments.

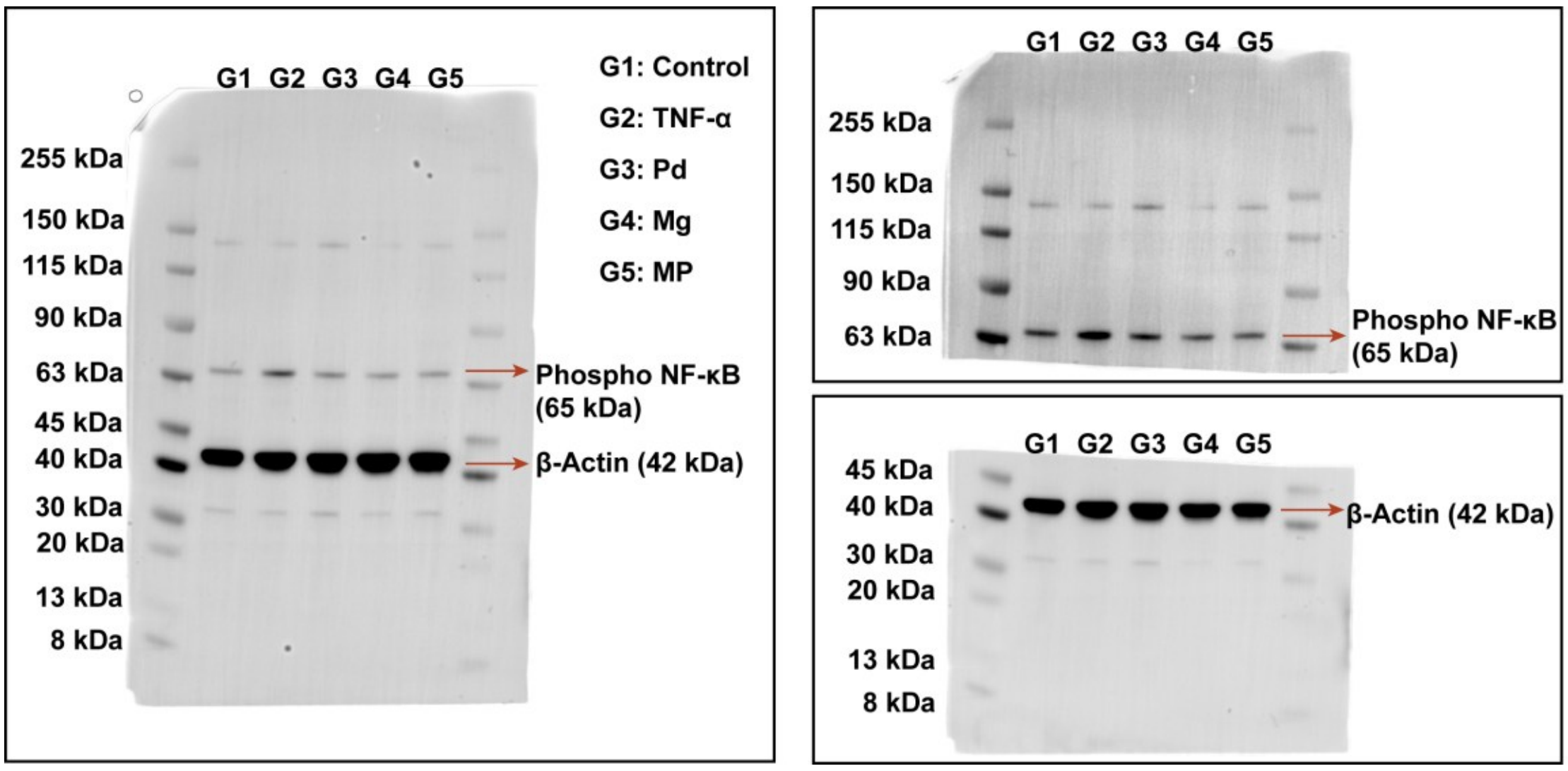

Figure S24. The complete results of western blotting of phosphorylated NF-κB expression in TNF-α induced HaCaT cells after different treatments.

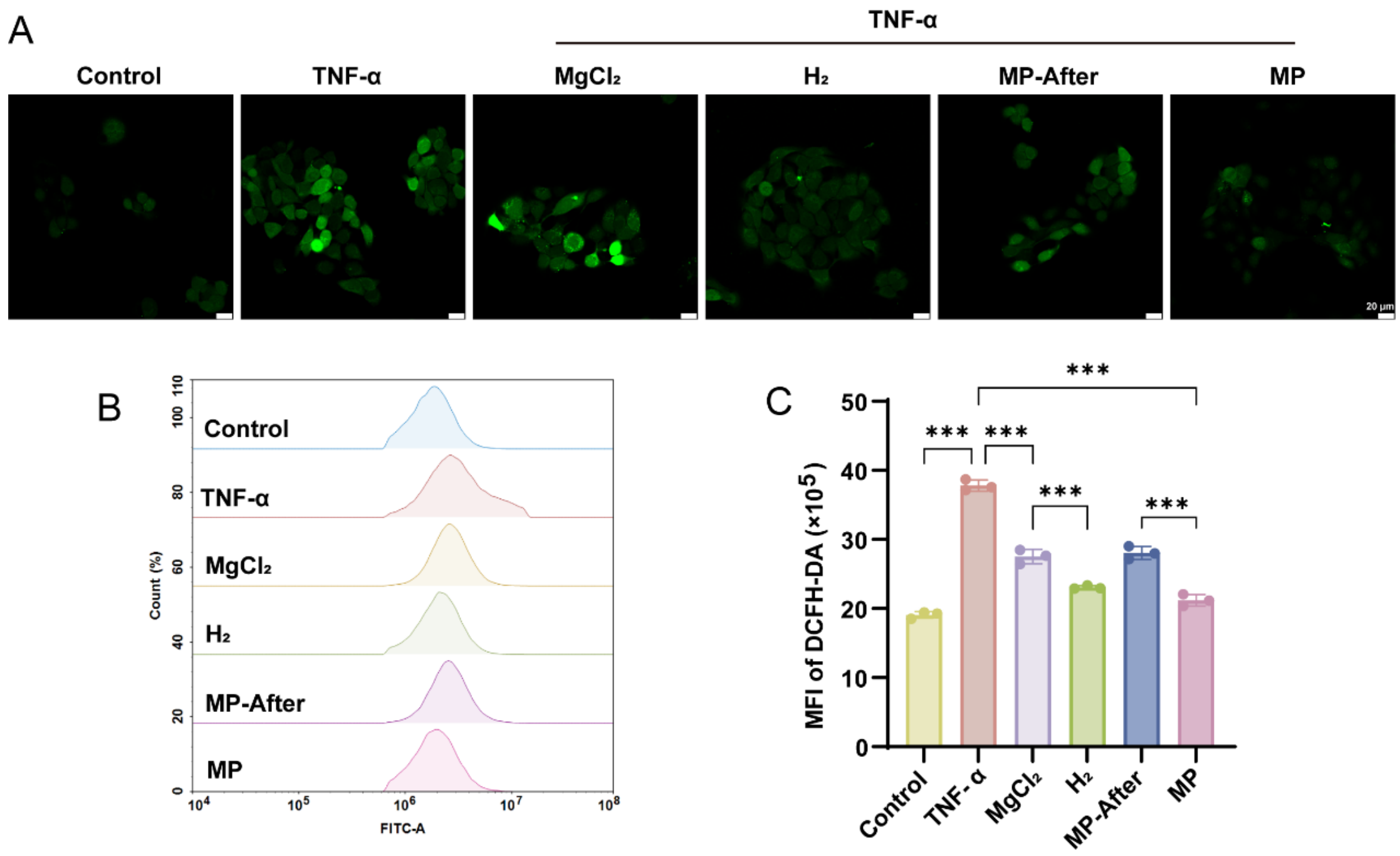

Figure S25. The antioxidant ability of Mg^{2+}, H_2, MP-After, and MP application in TNF-α-induced HaCaT cells. (A) The confocal images of HaCaT cells labeled by DCFH-DA probes (Scale bar: 20 μm). Flow cytometric examination (B) and quantitative analysis (C) of ROS in TNF-α-induced HaCaT cells after different treatments. Data were shown as the mean ± SD. For each group, n = 3 independent samples in (C). *** $p < 0.001$.

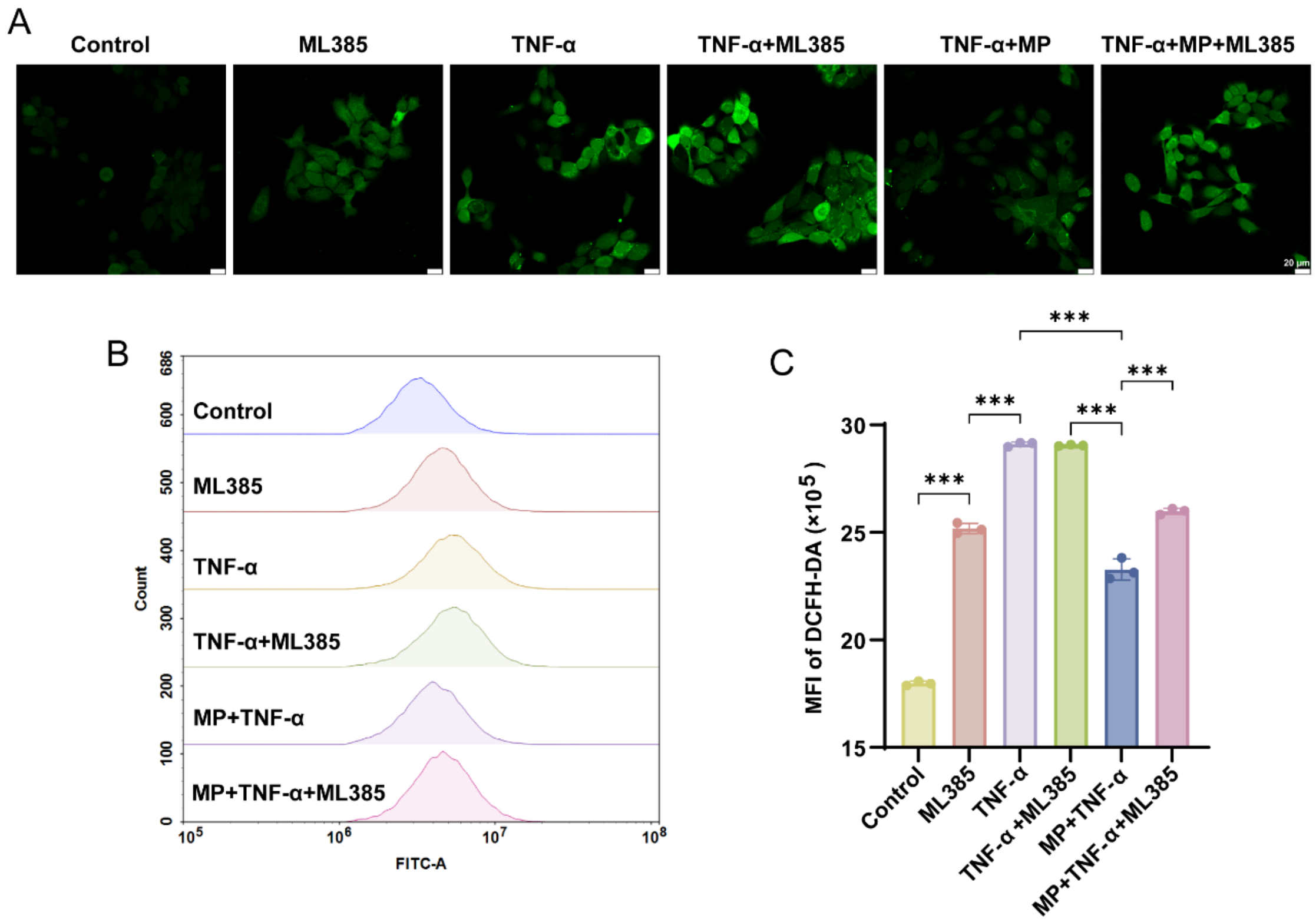

Figure S26. The effect of NRF2 activation on oxidative stress. (A) The confocal images of HaCaT cells labeled by DCFH-DA probes (Scale bar: 20 μm). Flow cytometric examination (B) and quantitative analysis (C) of ROS in HaCaT cells under different treatments. Data were shown as the mean ± SD. For each group, n = 3 independent samples in (C). *** $p < 0.001$.

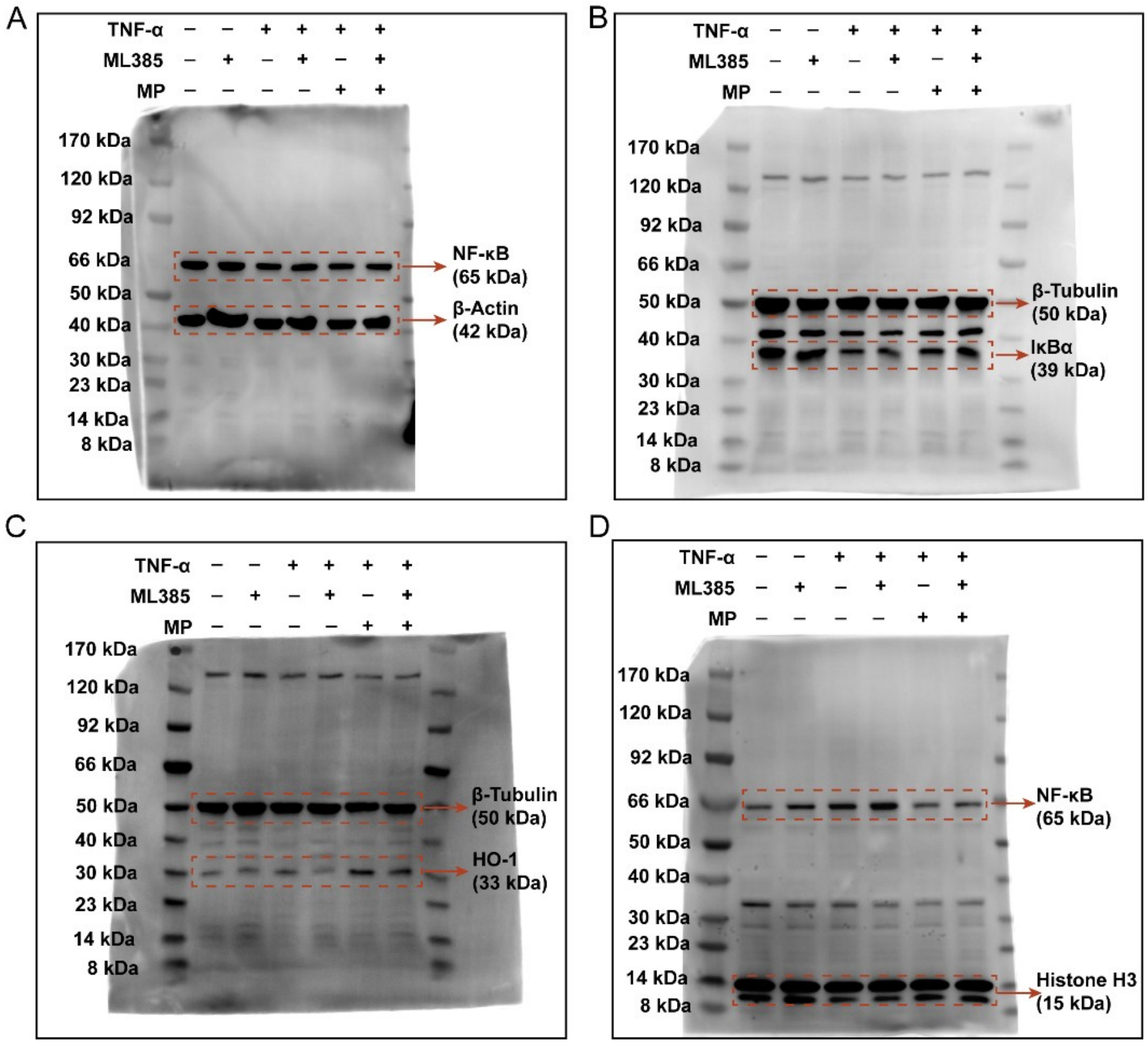

Figure S27. Western blot analysis of total NF-κB (A), IκBα (B), HO-1 (C), and nuclear NF-κB (D) proteins expression in HaCaT cells.

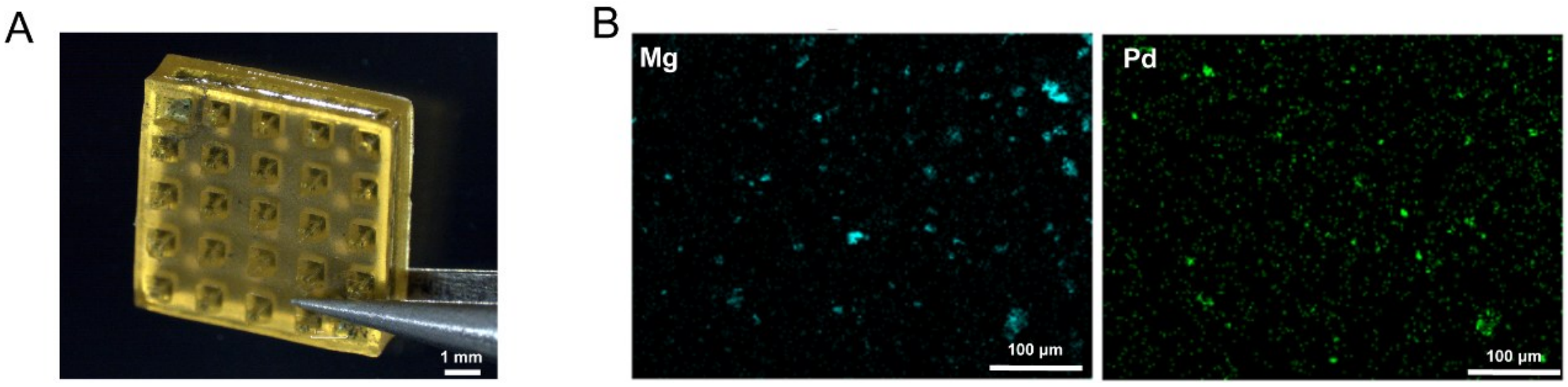

Figure S28. (A) The overview image of MP-MN (Scale bar: 1 mm). (B) EDS mappings of MP-MN (Scale bar: 100 μm).

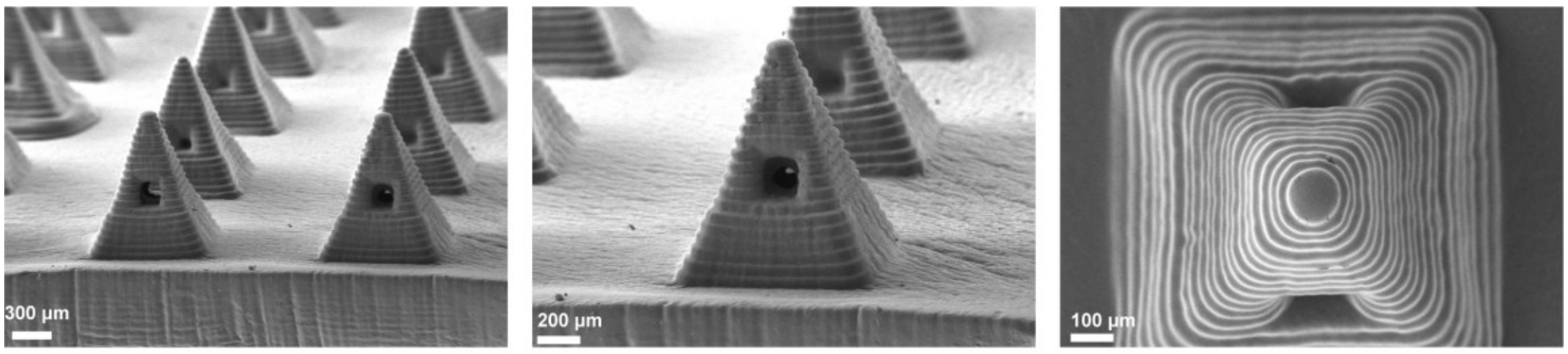

Figure S29. The SEM images of hollow MN from different angles (Scale bar: 300 µm; Scale bar: 200 µm; Scale bar: 100 µm).

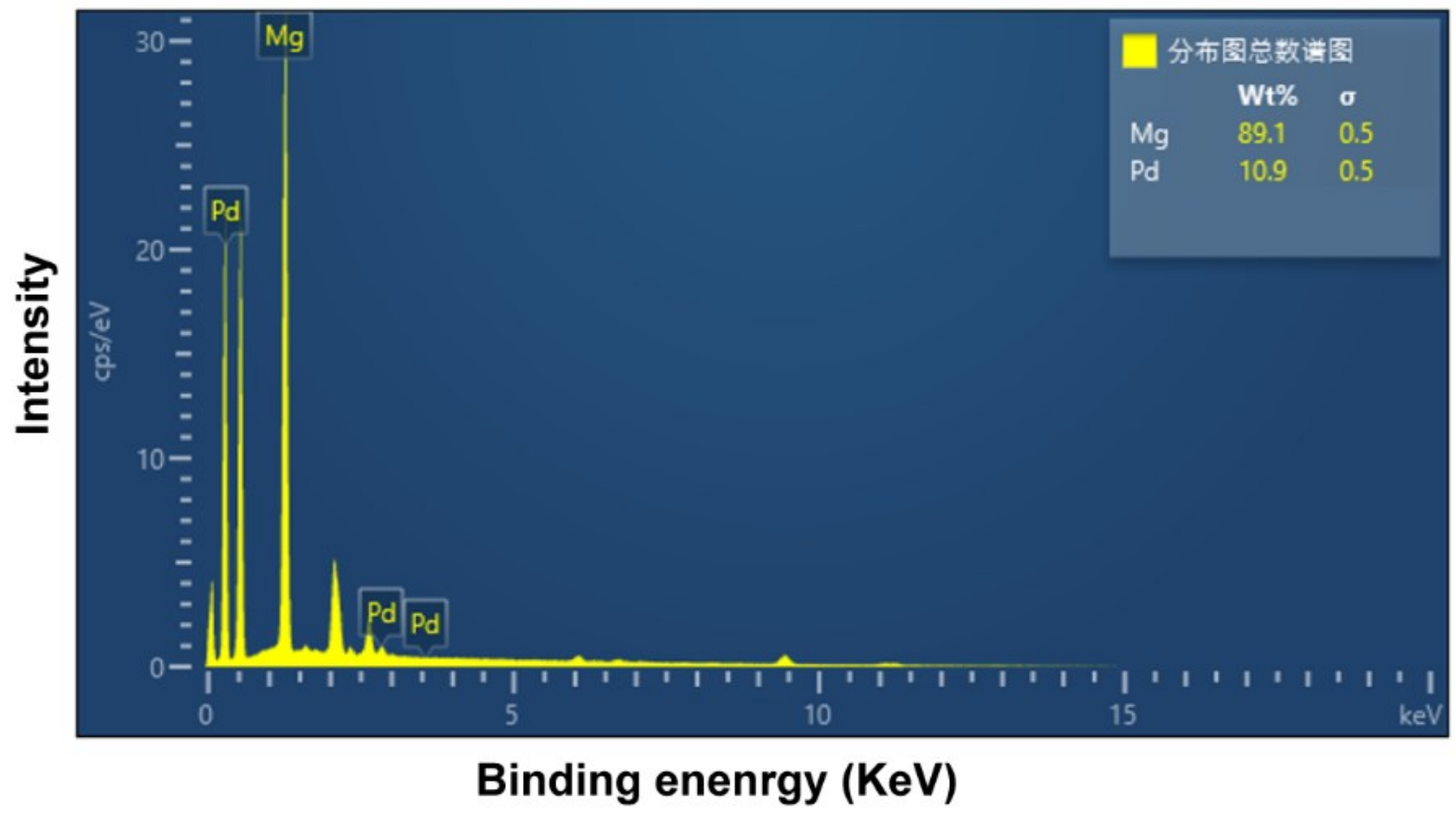

Figure S30. EDS sum Spectrum of MP.

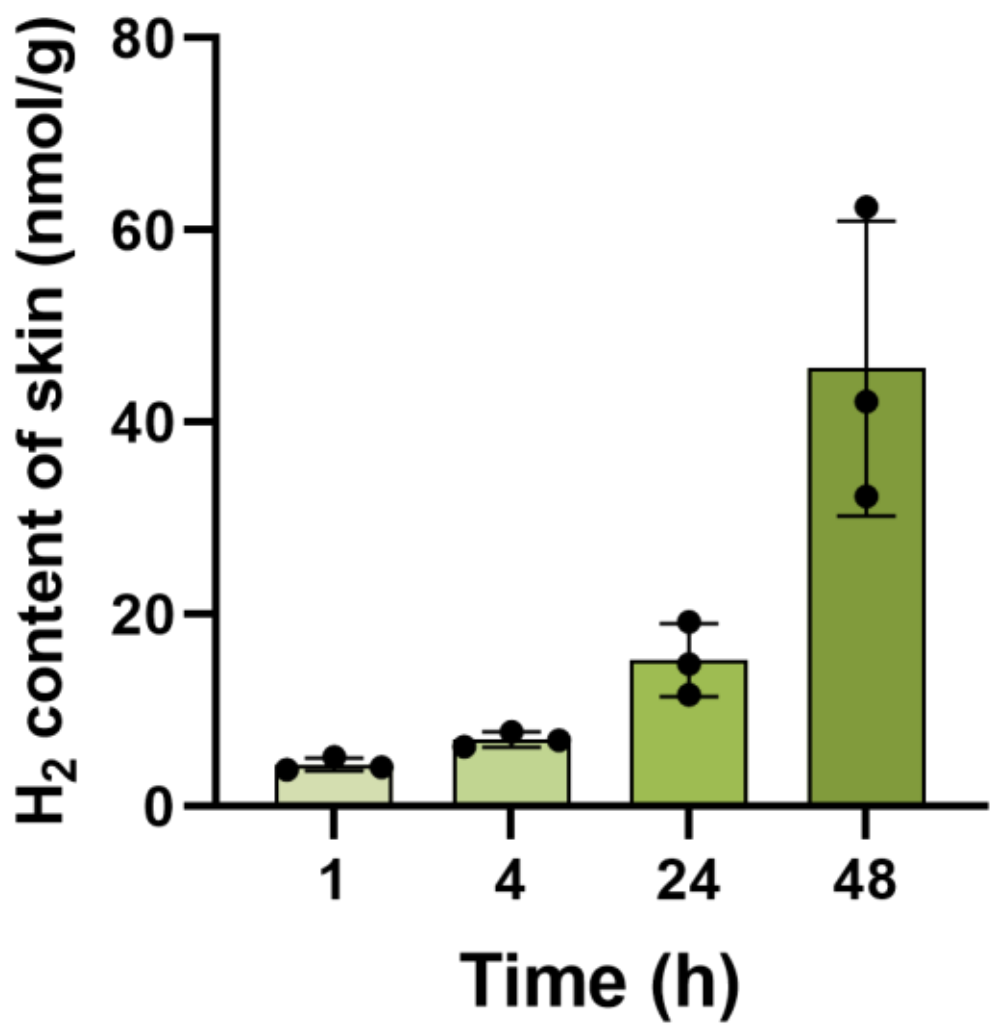

Figure S31. The H_2 content in skin tissue after MP-MN application during 48 h. Data were shown as the mean ± SD. For each time point, n = 3 independent samples.

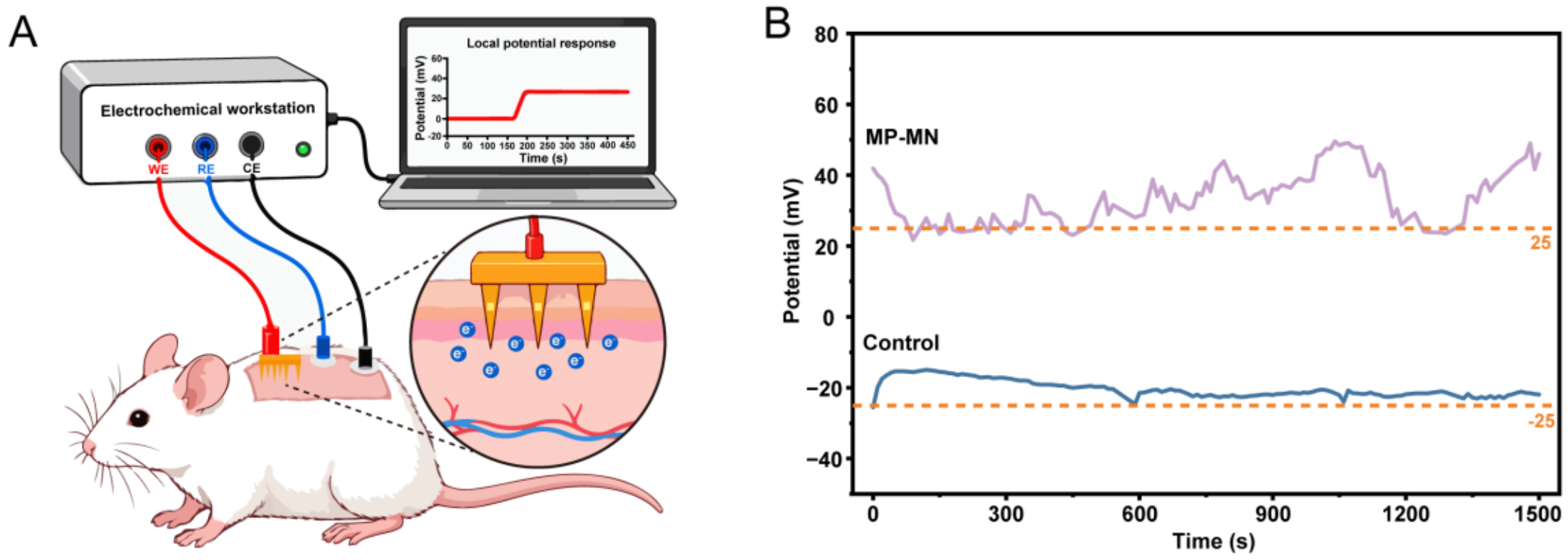

Figure S32. Local potential changes induced by MP-MN in mouse skin. (A) Schematic illustration of local skin potential monitoring. (B) Local potential–time curves in mouse skin without and with MP-MN application.

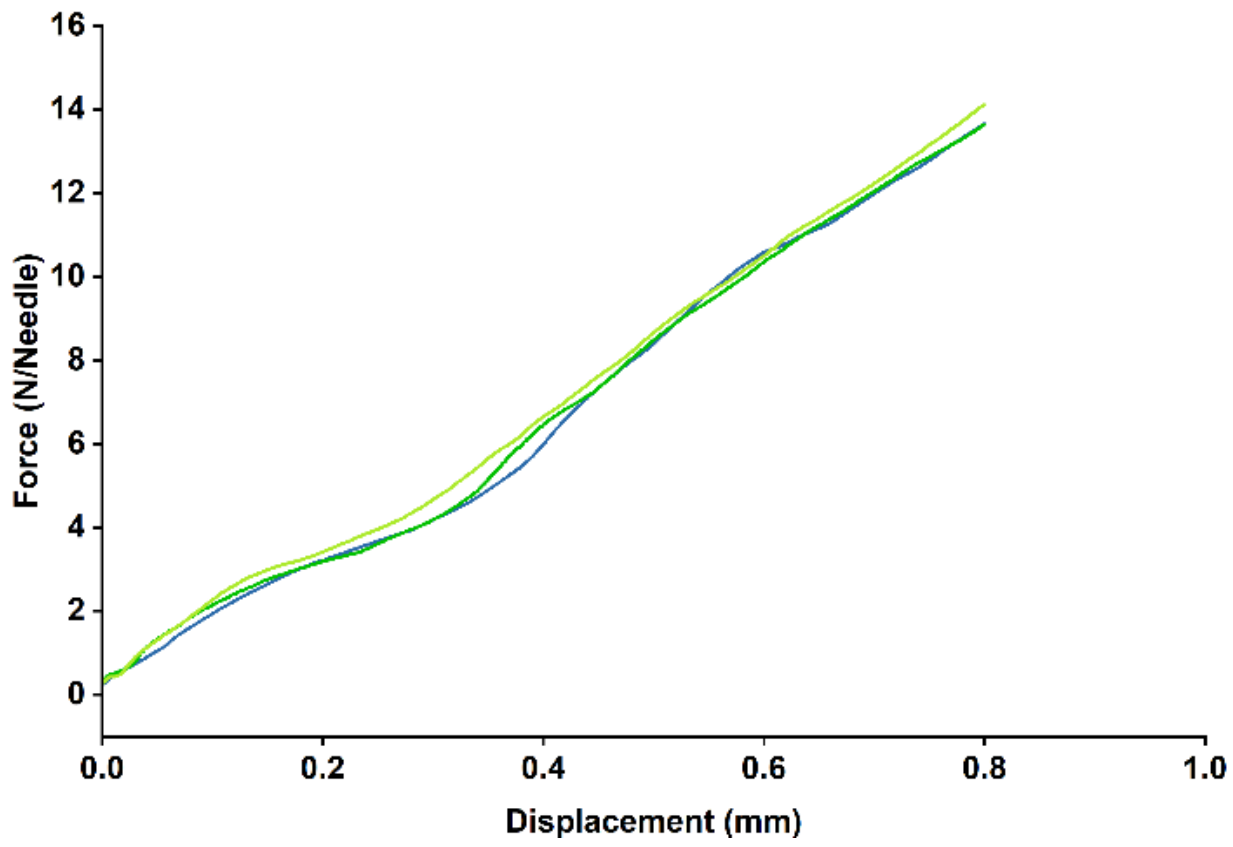

Figure S33. Stress–displacement curve for the single needle of MP-MN (n =3).

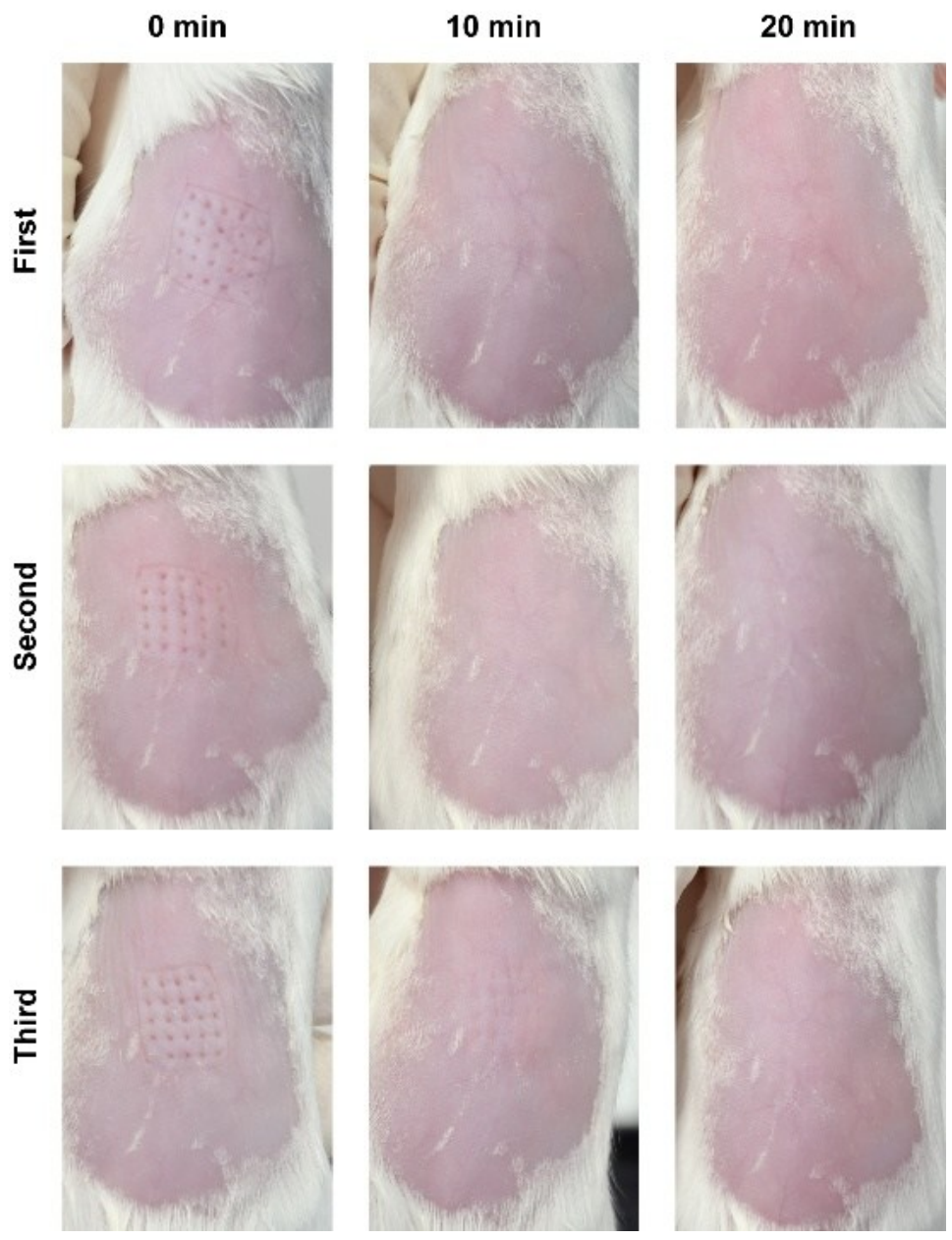

Figure S34. Time-dependent images showing the recovery of skin punctures at various time points after three consecutive applications of MP-MN.

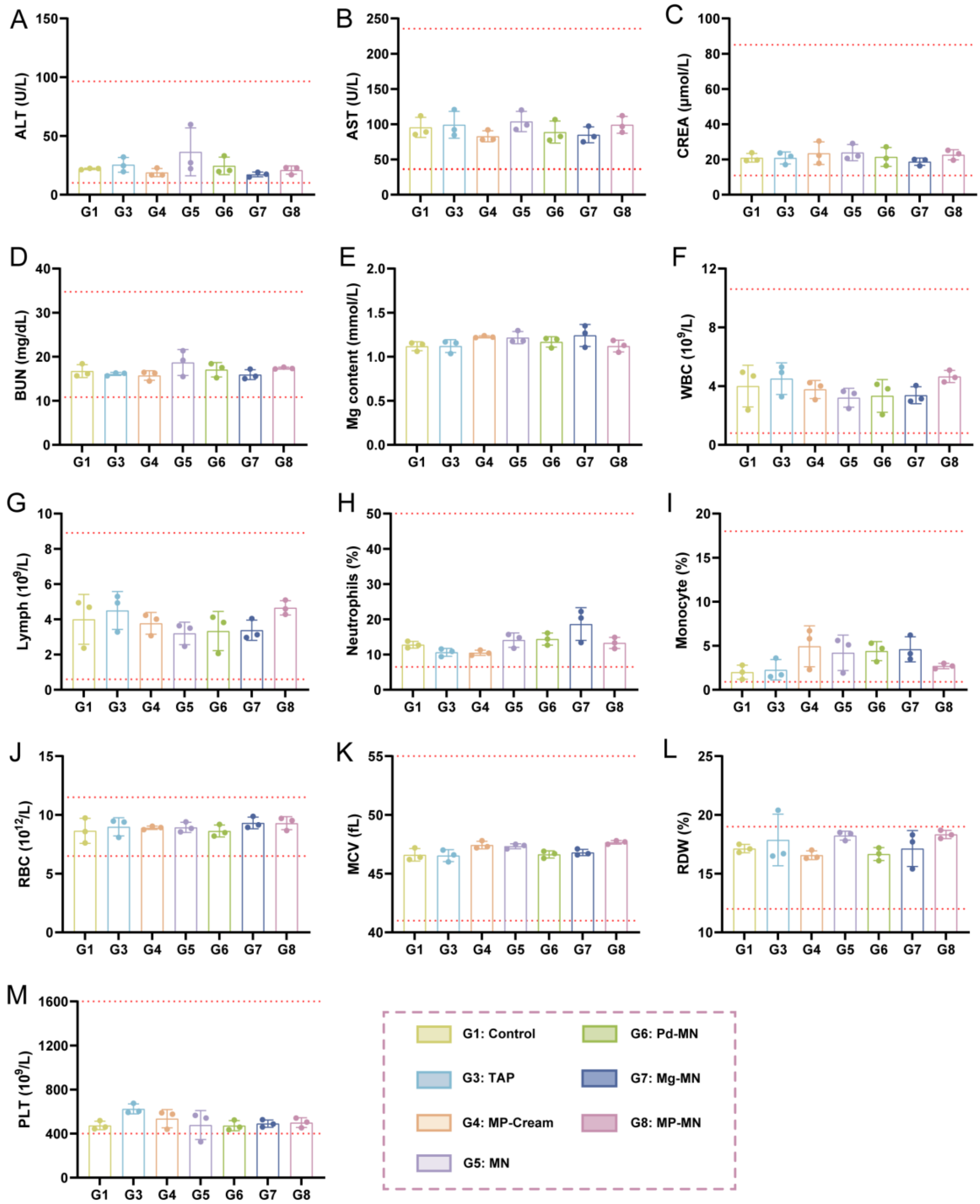
A
ALT (U/L)
B
AST (U/L)
C
CREA (μmol/L)
D
BUN (mg/dL)
E
Mg content (mmol/L)
F
WBC (10^9/L)
G
Lymph (10^9/L)
H
Neutrophils (%)
I
Monocyte (%)
J
RBC (10^{12}/L)
K
MCV (fL)
L
RDW (%)
M
PLT (10^9/L)
G1 G3 G4 G5 G6 G7 G8
G1: Control
G3: TAP
G4: MP-Cream
G5: MN
G6: Pd-MN
G7: Mg-MN
G8: MP-MN

182

Figure S35. The biosafety evaluation of the blood for healthy mice after various treatment. The content of serum biochemical indicators, including (A) alanine aminotransferase (ALT), (B) aspartate aminotransferase (AST), (C) creatinine (CREA), (D) blood urea nitrogen (BUN), and (E) Mg. The levels of blood routine parameters, including (F) white blood cell (WBC), (G) lymphocyte (Lymph), (H) neutrophils, (I) monocyte, (J) red blood cell (RBC), (K) mean corpuscular volume (MCV), (L) red cell distribution width (RDW), (M) platelet (PLT). The red line suggests the normal reference range of parameters in mice. Data are shown as the mean ± SD. For each group, n = 3 independent samples.

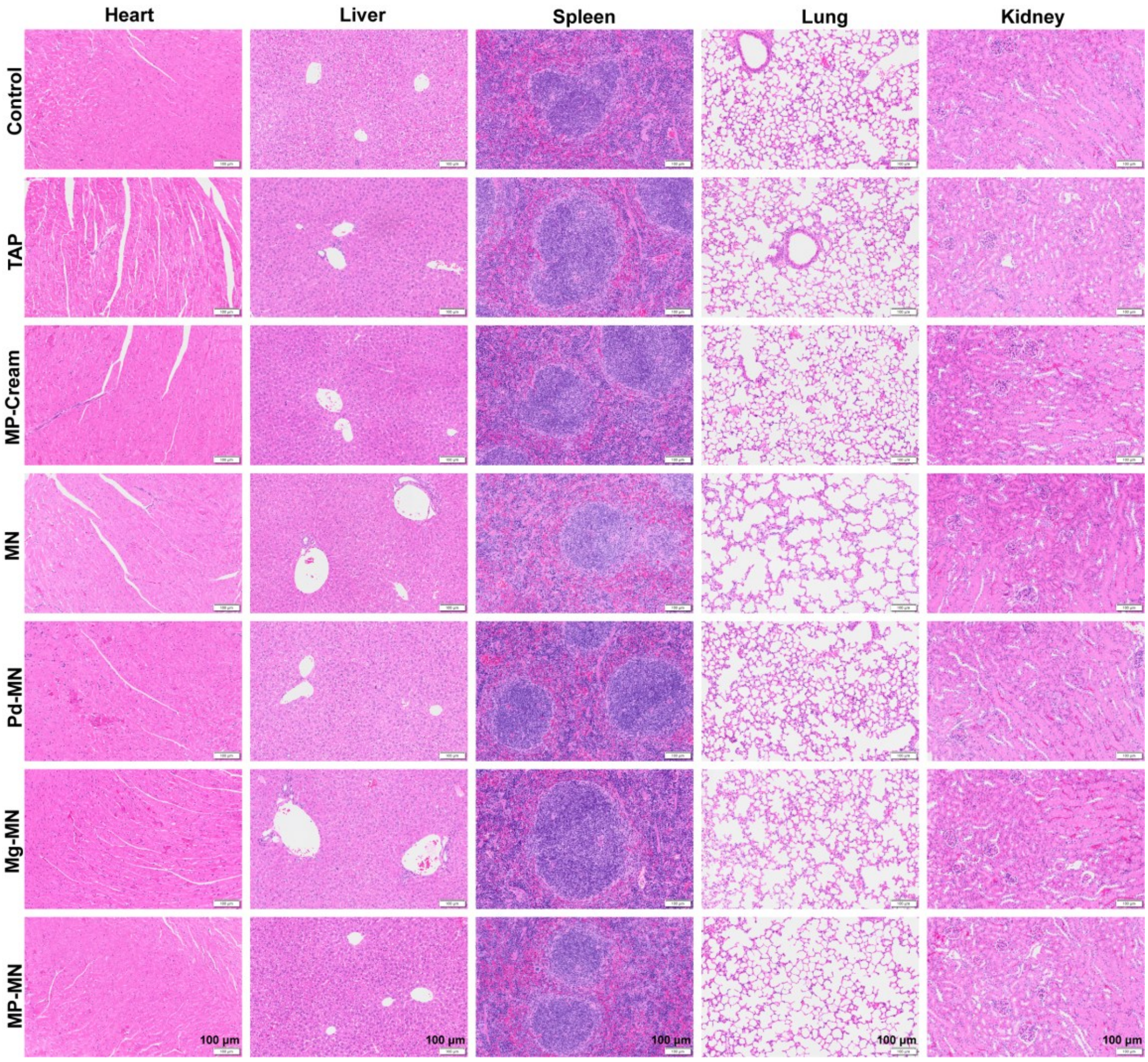

Figure S36. Representative H&E staining images of major organs from each group (Scale bar: 100 μm).

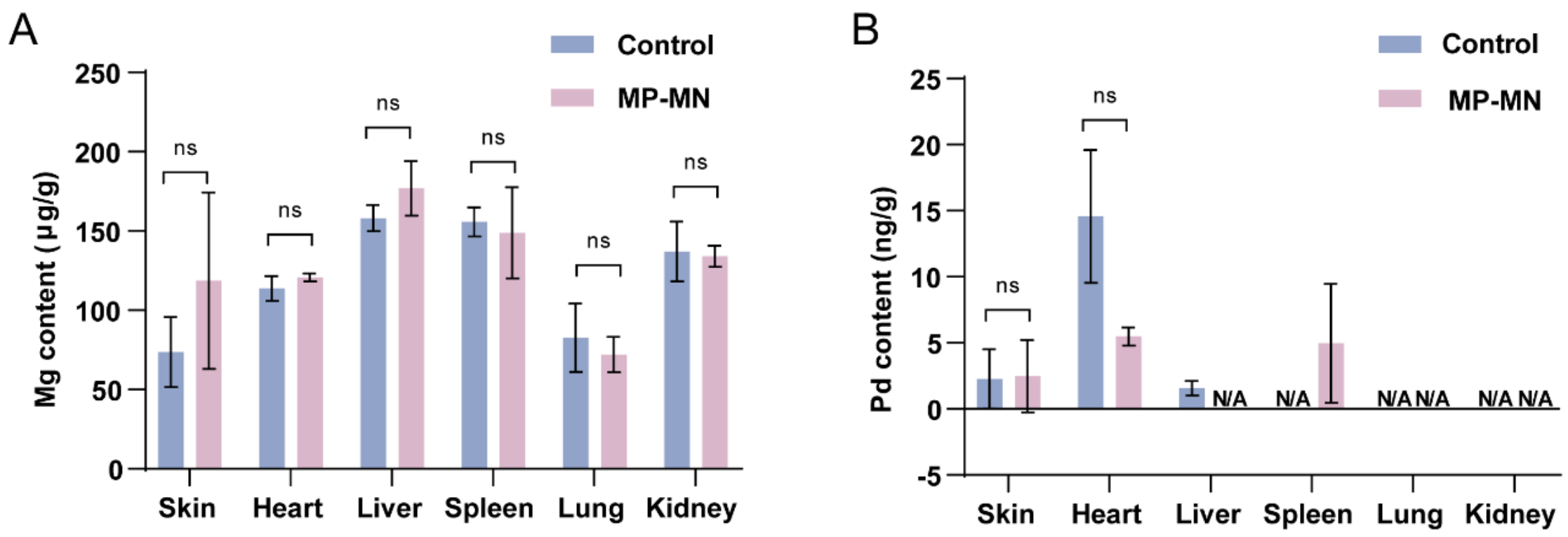

Figure S37. Mg (A) and Pd (B) contents of the skin, hearts, livers, spleens, lungs and kidneys of mice from different treatment groups after 24 h. Data are shown as the mean ± SD. For each group, n = 3 independent samples.

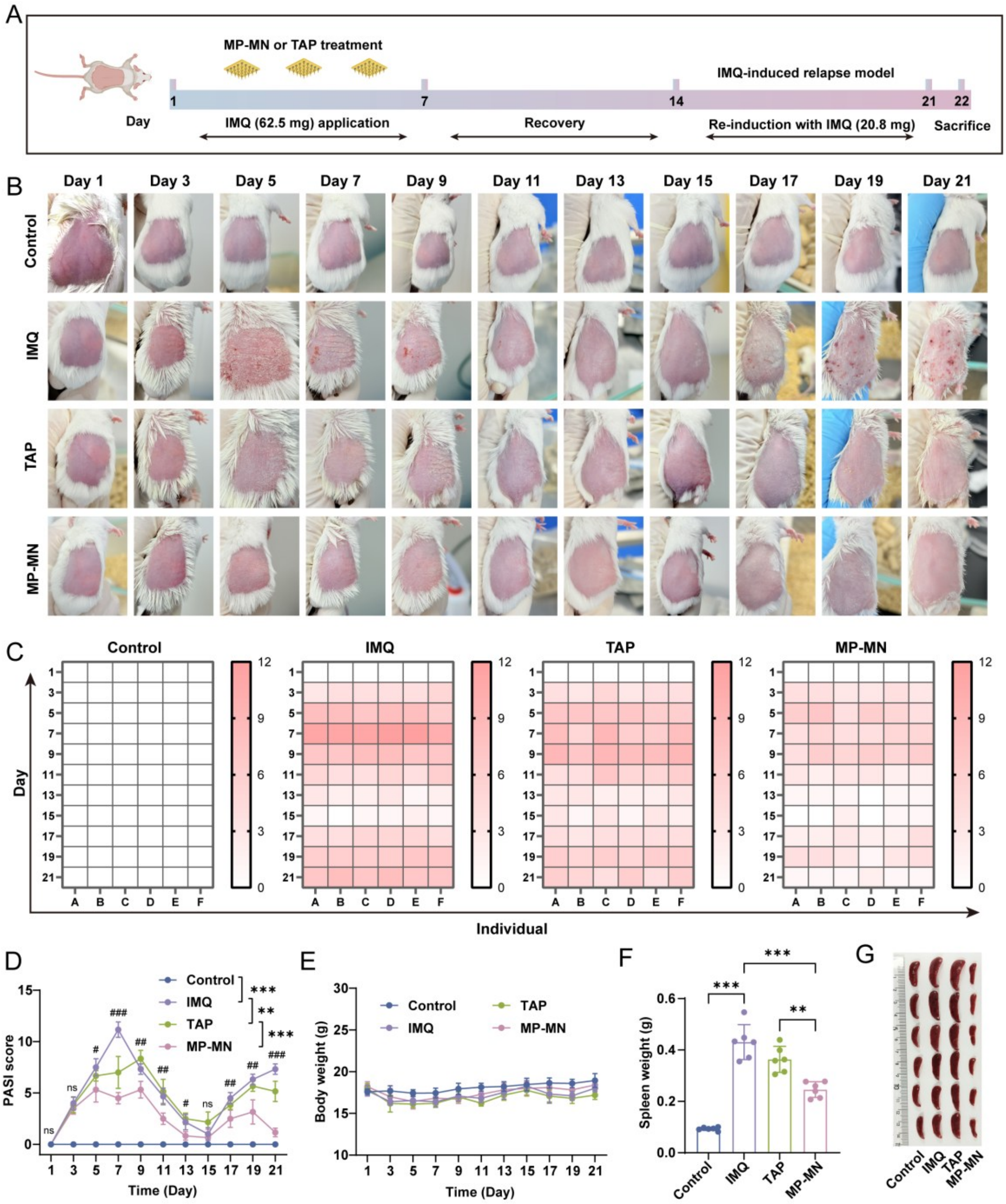
A
MP-MN or TAP treatment
IMQ-induced relapse model
Day
1
7
14
21
22
IMQ (62.5 mg) application
Recovery
Re-induction with IMQ (20.8 mg)
Sacrifice
B
Day 1
Day 3
Day 5
Day 7
Day 9
Day 11
Day 13
Day 15
Day 17
Day 19
Day 21
Control
IMQ
TAP
MP-MN
C
Control
IMQ
TAP
MP-MN
Day
Individual
A B C D E F
D
Control
IMQ
TAP
MP-MN
PASI score
Time (Day)
E
Control
IMQ
TAP
MP-MN
Body weight (g)
Time (Day)
F
Spleen weight (g)
Control
IMQ
TAP
MP-MN
G
Control
IMQ
TAP
MP-MN

Figure S38. The long-term therapeutic effect of MP-MN in a recurrent psoriasis mouse model. (A) Schematic diagram of recurrent psoriasis mouse model establishment and administration protocol. (B) Representative images of mouse dorsal skins of various groups from days 1–21. (C) Heatmap of the total PASI score of each individual during the experiment. (D) PASI score in each group. (E) Body weight trends in each group during the experiment. Spleen weight (F) and size (G) in different groups at the endpoint. Data were shown as the mean ± SD. For each group, n = 6 independent samples in (F); $^{**}p < 0.01$, $^{***}p < 0.001$. (D) $^{**}p < 0.01$, $^{***}p < 0.001$ among all groups on Day 21; $^{\#}p < 0.05$, $^{\#\#}p < 0.01$, and $^{\#\#\#}p < 0.001$ between Model group and MP-MN group.

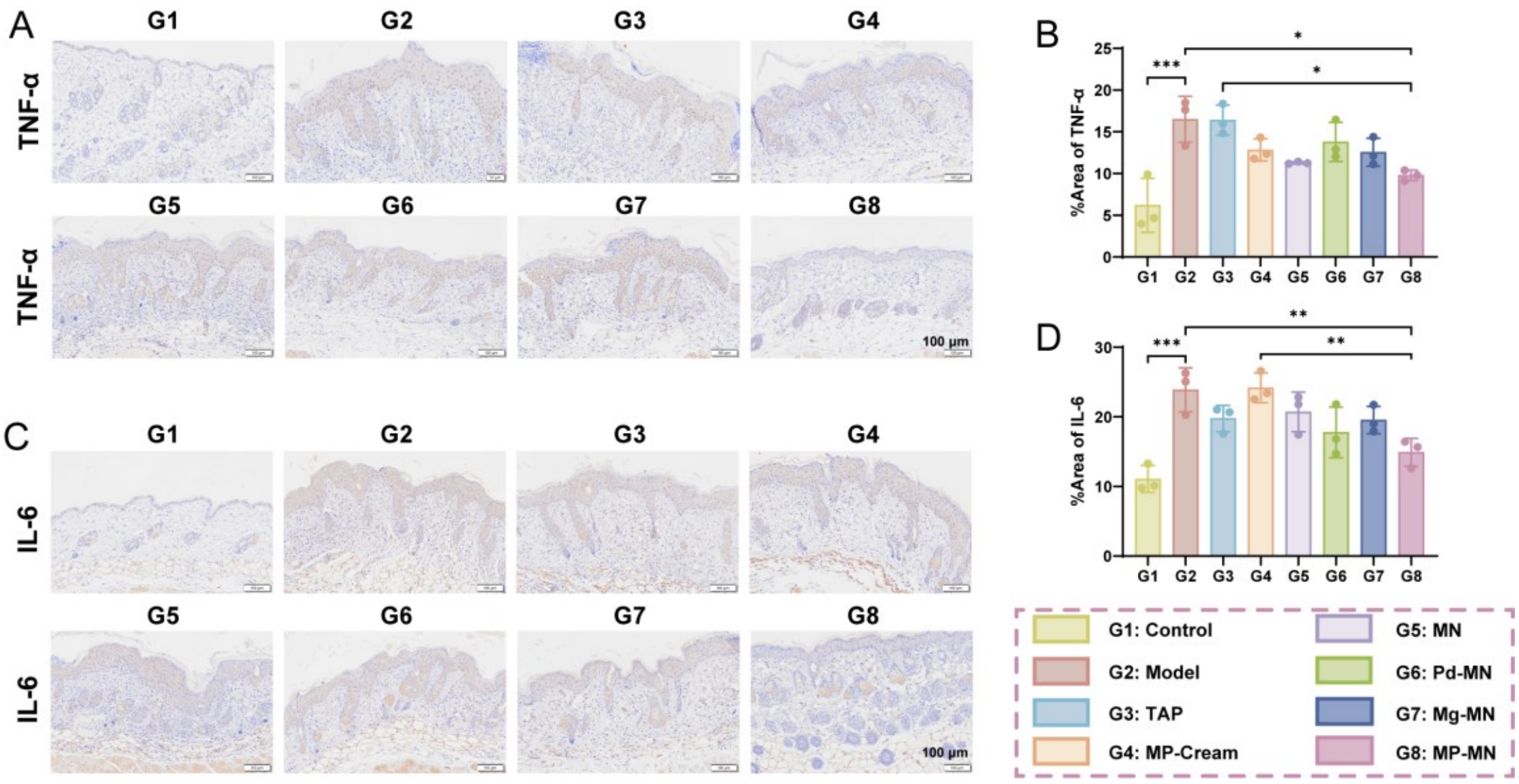

Figure S39. The TNF-α and IL-6 expression of psoriatic skin in mice. (A) Immunohistochemical staining images of TNF-α (Scale bar: 100 μm). (B) Statistical analysis of signal for TNF-α. (C) Immunohistochemical staining images of IL-6 (Scale bar: 100 μm). (D) Statistical analysis of signal for IL-6. Data were shown as the mean ± SD. For each group, n = 3 independent samples in (B, D). * $p < 0.05$, ** $p < 0.01$, *** $p < 0.001$.

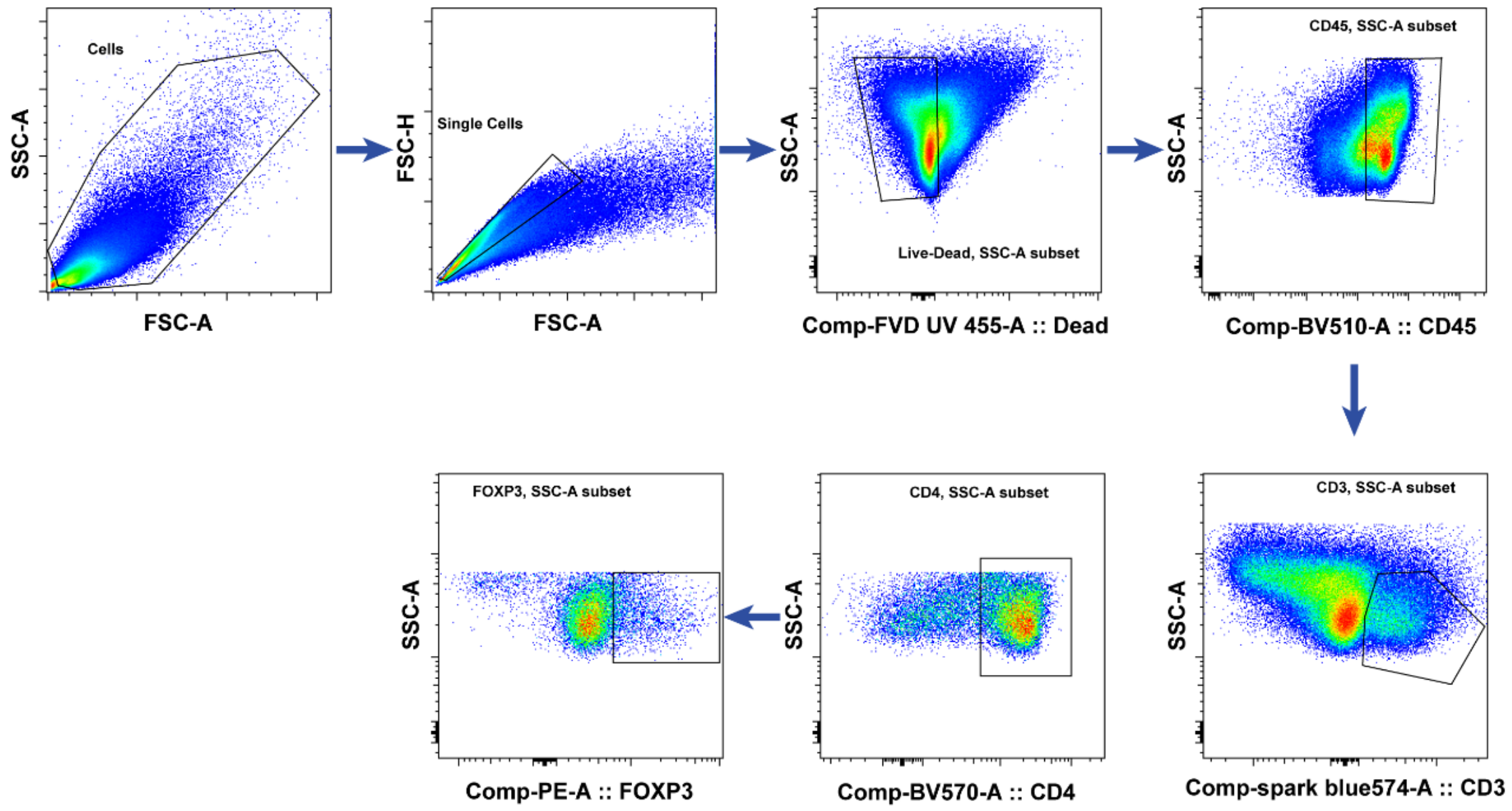

Figure S40. Gating strategy for Treg cells ($FoxP3^+$) within the $CD4^+$ cell population in flow cytometry analysis.

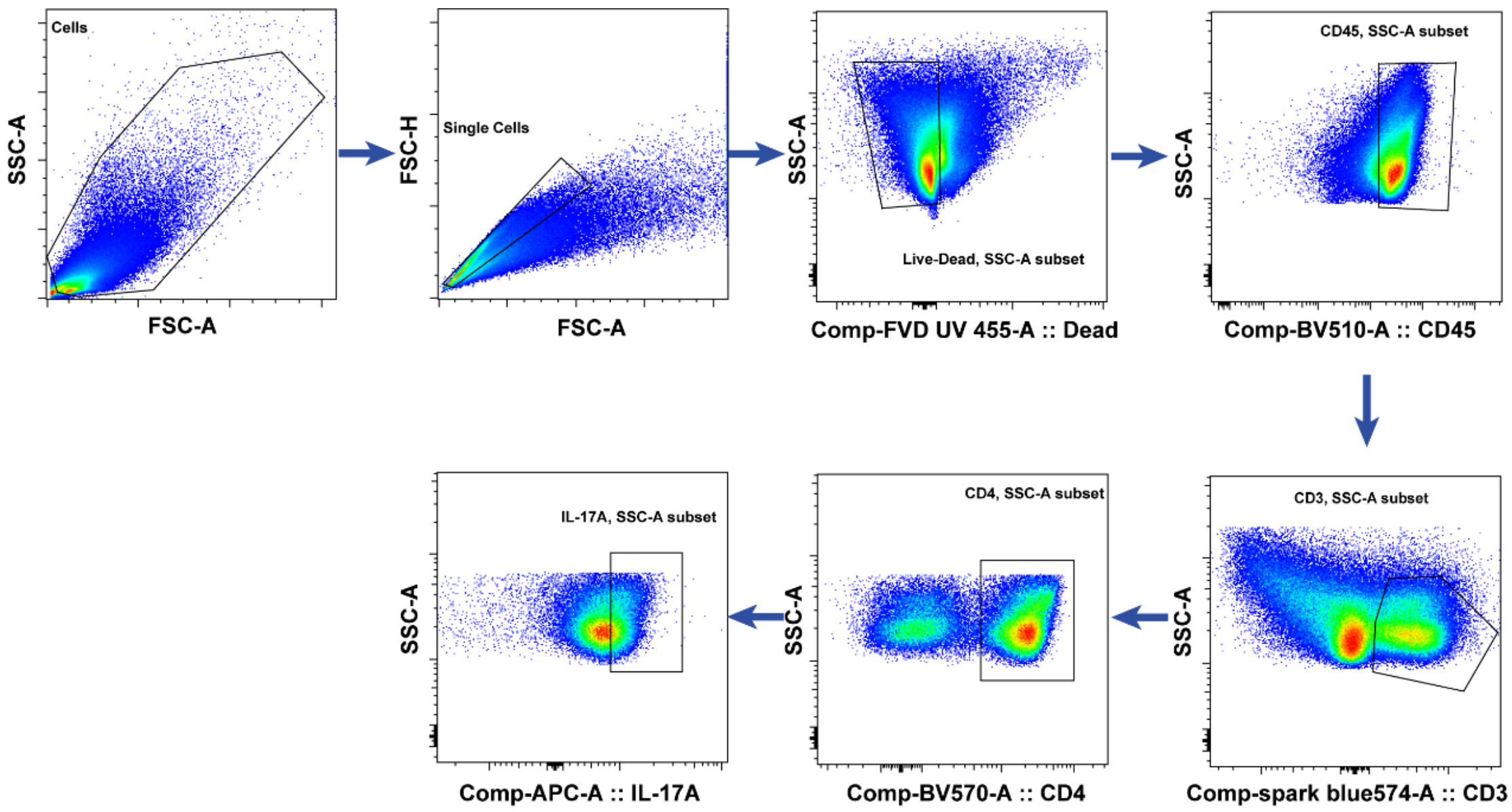

Figure S41. Gating strategy for Th17 cells (IL-17A$^+$) within the CD4$^+$ cell population in flow cytometry analysis.

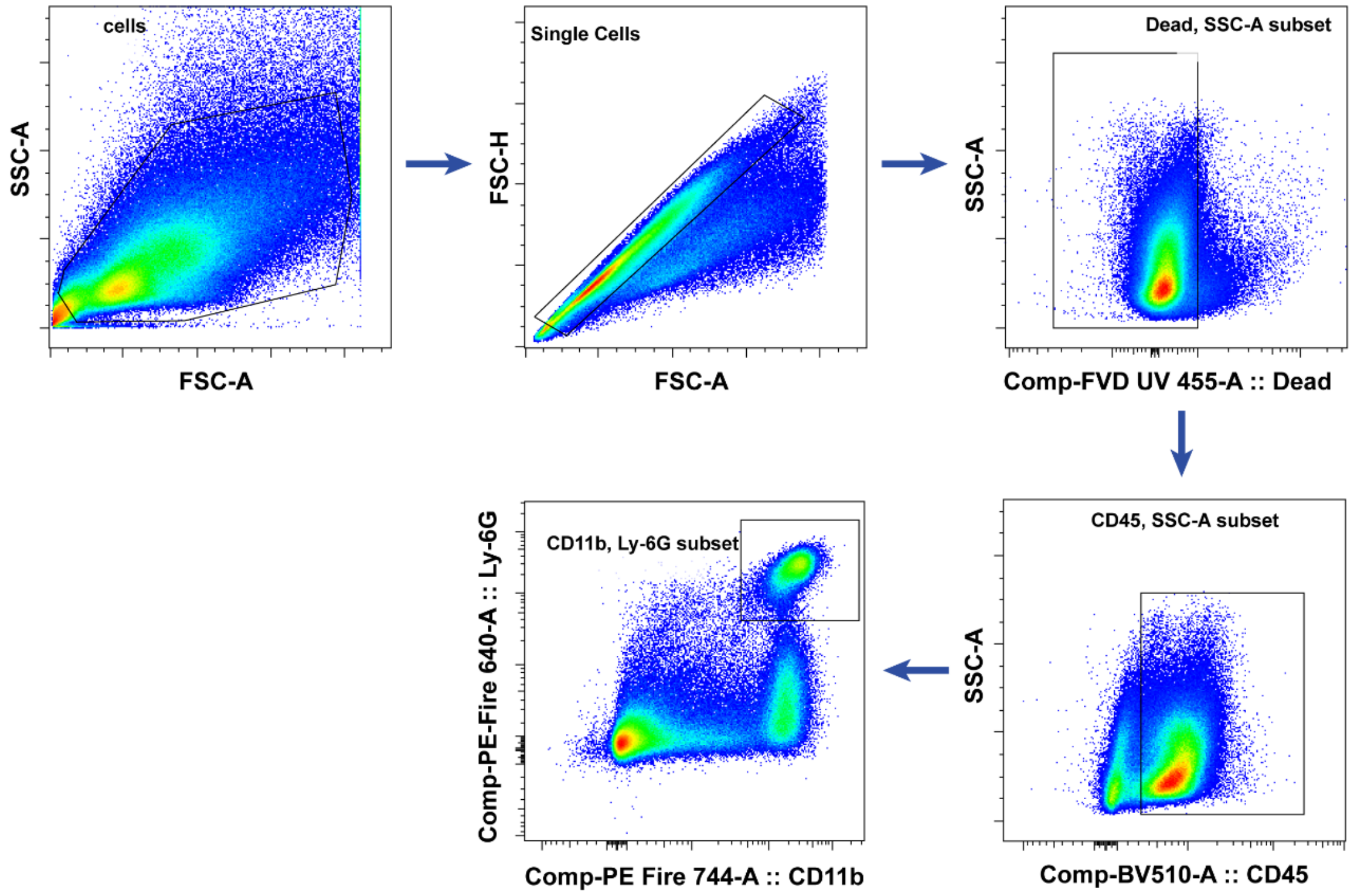

Figure S42. Gating strategy for neutrophils ($CD11b^+$ $Ly\text{-}6G^+$) within the $CD45^+$ cell population in flow cytometry analysis.

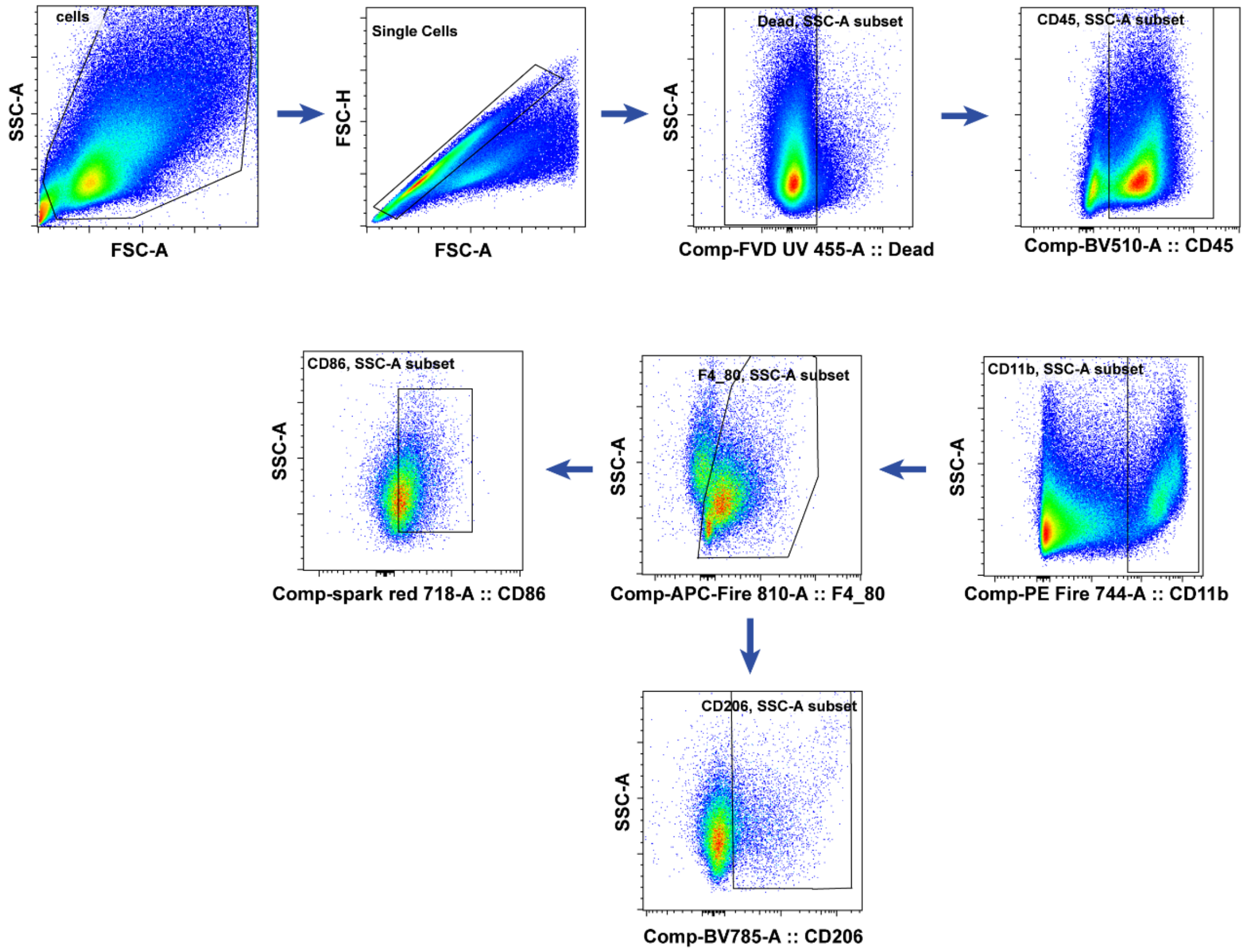

Figure S43. Gating strategy for macrophages within the F4/80^{+} cell population in flow cytometry analysis.

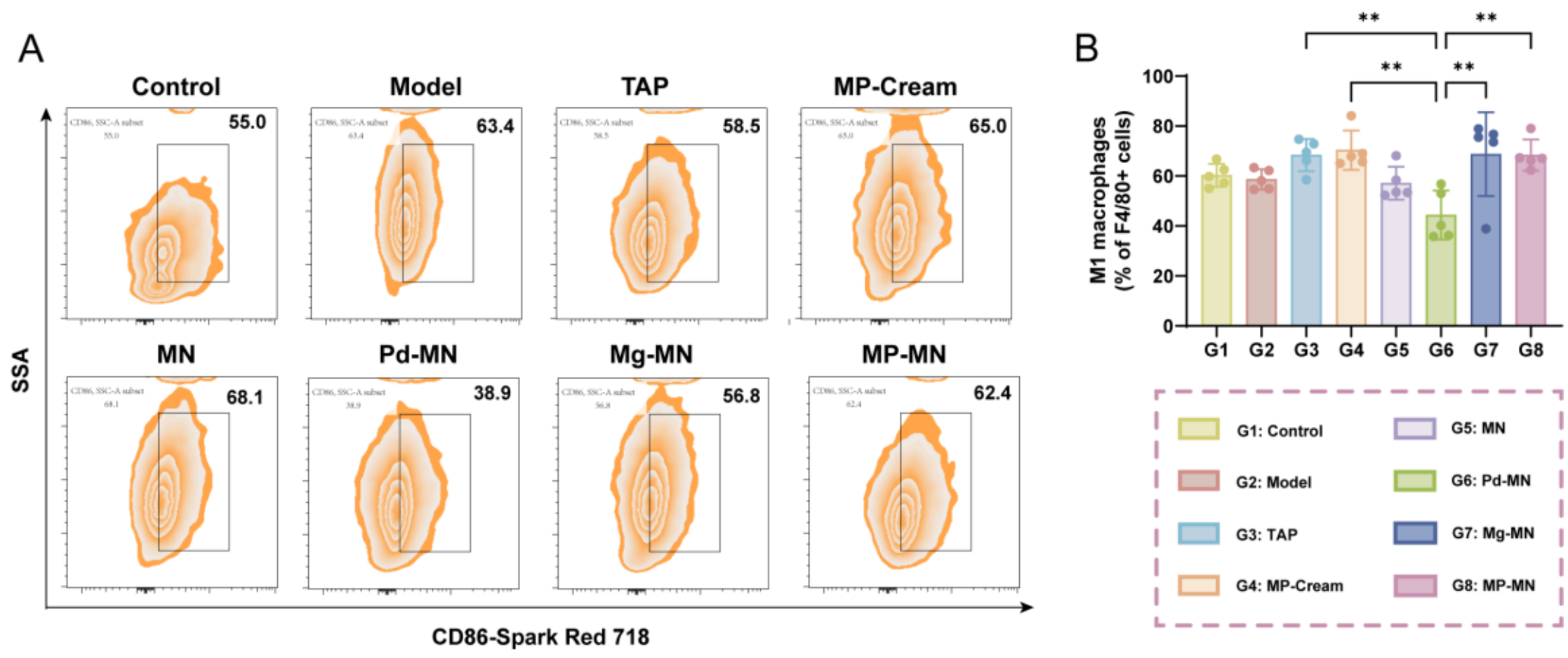

Figure S44. Representative flow cytometry of M1 macrophages (A) and its statistical analysis in various groups (B). Data were shown as the mean ± SD. For each group, n = 5 independent samples in (B). ** $p < 0.01$.

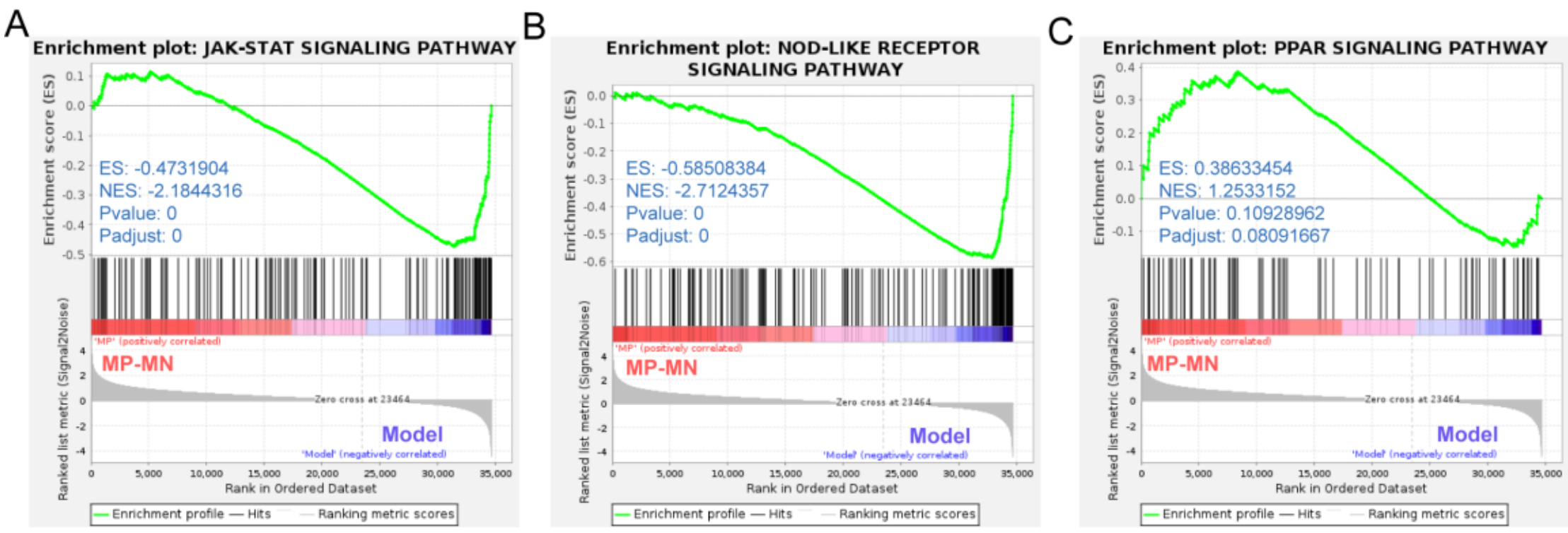

Figure S45. The GSEA analysis between the Model and MP-MN groups. (A) JAK-STAT signaling pathway. (B) NOD-like receptor. Signaling pathway. (C) PPAR signaling pathway. For each group, n = 4 independent samples.

Table S1

Table S1. Quality assessment of MP-MN (n =3).

Parameter	Mean ± SD	CV (%)
Substrate width (mm)	7.383 ± 0.061	0.828
Tip height (mm)	1.111 ± 0.015	1.326
Base length of tips (mm)	0.924 ± 0.010	1.038
Inter-tip spacing (mm)	0.828 ± 0.017	2.108
MP loading (mg/all tips)	2.88 ± 0.27	9.48
MP loading (mg/patch)	11.92 ± 0.80	6.74

Table S2

Table S2. Observations of skin irritation and corrosion on mice skin after three consecutive applications of MP-MN.

Patch	Number of repetitions	Skin corrosion (SCT)	Skin irritation (SIT)
MP-MN	3	No observation	No observation
